# Low-dose TNF-α drives malignant progression and lipid metabolism in glioblastoma through the TRAF2-FASN axis

**DOI:** 10.1038/s41420-026-03087-x

**Published:** 2026-04-09

**Authors:** Maorong Cai, Yang Liu, Xinyu Mao, Yiping Wu, Shoujia Sun, Jing Zhou, Tao Huang, Jiantong Jiao

**Affiliations:** 1 The Affiliated Wuxi People’s Hospital of Nanjing Medical University, Wuxi People’s Hospital, Wuxi Medical Center, Nanjing Medical University, Nanjing, China; 2 Department of Neurosurgery, The Affiliated Wuxi People’s Hospital of Nanjing Medical University, Wuxi People’s Hospital, Wuxi Medical Center, Nanjing Medical University, Wuxi, Jiangsu China; 3 Department of Neurosurgical Intensive Care Unit & Emergency Neurosurgery, Qilu Hospital of Shandong University, Jinan, Shandong China; 4 School of Medicine, Cheeloo College of Medicine, Shandong University, Jinan, Shandong China; 5 Department of Pathology, The Affiliated Wuxi People’s Hospital of Nanjing Medical University, Wuxi People’s Hospital, Wuxi Medical Center, Nanjing Medical University, Wuxi, Jiangsu China; 6 Department of Neurosurgery, Union Hospital, Tongji Medical College, Huazhong University of Science and Technology, Wuhan, Hubei China

**Keywords:** Cancer microenvironment, Cancer metabolism, Drug development, Oncogenes, CNS cancer

## Abstract

Lipid metabolism plays a critical role in the progression of cancers, including glioblastoma (GBM). Tumor necrosis factor-α (TNF-α) exhibits a dual role in the tumor microenvironment: at high concentrations, it induces cell death and exerts anti-tumor effects, while at low doses, it demonstrates pro-tumorigenic activity. This study investigates the regulatory effects of low-dose TNF-α on the malignant behavior and lipid metabolism of GBM. The results show that low-dose TNF-α (10 ng/mL) significantly promotes GBM cell malignant phenotypes and lipid droplet accumulation via the Tumor Necrosis Factor Receptor (TNFR) signaling pathway, a process dependent on its downstream adaptor protein, Tumor necrosis factor receptor-associated factor 2 (TRAF2). Mechanistically, TRAF2 interacts with Fatty acid synthase (FASN) through its TRAF domain and, serving as an E3 ubiquitin ligase, leverages its RING domain to mediate K63-linked polyubiquitination of FASN. This enhances FASN protein stability, promotes lipid synthesis, and ultimately drives tumor progression. Furthermore, through virtual screening, the small-molecule compound Jionoside B1 was identified to target the RING domain of TRAF2, effectively inhibiting K63-linked ubiquitination of FASN and disrupting the TRAF2-FASN interaction and protein accumulation. In both in vitro and in vivo experiments, Jionoside B1 significantly suppressed lipid synthesis and attenuated tumor growth. This study systematically elucidates the mechanism by which low-dose TNF-α regulates lipid metabolism and promotes GBM malignant progression via the TRAF2-FASN axis, providing not only new insights into the “double-edged sword” role of TNF-α in the tumor microenvironment but also a potential novel therapeutic strategy for targeting this pathway in GBM treatment.

## Introduction

Glioblastoma (GBM) is the most aggressive and lethal primary malignant brain tumor, characterized by diffuse infiltration, persistent recurrence, and high resistance to conventional therapies [1, 2]. Despite aggressive multimodal treatment involving maximal safe surgical resection combined with radiotherapy and chemotherapy, patient prognosis remains extremely poor, with a median overall survival of less than 15 months [3, 4]. In recent years, metabolic reprogramming has been recognized as a core hallmark of GBM. The aberrant activation of lipid metabolism, in particular, provides rapidly proliferating tumor cells with essential building blocks for biomembranes, energy, and signaling molecules, representing a potential therapeutic target [5–7]. Fatty Acid Synthase (FASN), the key metabolic enzyme that catalyzes the de novo synthesis of long-chain fatty acids, is significantly overexpressed in GBM and is closely associated with poor prognosis [8]. The oncogenic functions of FASN depend not only on its catalytic activity but are also precisely and synergistically regulated by various post-translational modifications, including ubiquitination, SUMOylation, acetylation, and palmitoylation. These modifications form a multi-layered regulatory network that finely controls FASN’s protein stability, enzymatic activity, subcellular localization, and non-canonical signaling functions, ultimately driving tumorigenesis and progression [9–12]. However, the specific upstream cytokines within the GBM tumor microenvironment (TME) that can regulate FASN remain elusive.

Fatty Acid Synthase (FASN), the key metabolic enzyme that catalyzes the de novo synthesis of long-chain fatty acids [13]. At high concentrations, TNF-α can directly induce tumor cell apoptosis by activating the caspase cascade and mitochondrial apoptotic pathway, disrupt the tumor vascular system to cause ischemic necrosis, and enhance anti-tumor responses from immune cells such as macrophages and T cells, thereby exerting anti-tumor effects [14]. In contrast, under chronic low-dose conditions, TNF-α transforms into a key mediator that promotes tumor progression. It not only sustains the activation of signaling pathways like NF-κB (e.g., via TNIP1-mediated glioma proliferation) and AP-1 (e.g., driving enhancer reprogramming in pancreatic cancer) to facilitate cell survival and proliferation but also maintains the self-renewal of glioma stem cells through the Vasorin-mediated glycolytic pathway. Additionally, it promotes resistance to anti-angiogenic therapy by activating vascular endothelial cells, collectively shaping an immunosuppressive microenvironment and driving malignant tumor progression [15–18]. This tumor-promoting effect raises the question of whether TNF-α may mediate lipid metabolism in GBM by regulating the stability of key metabolic enzymes such as FASN.

The signaling of TNF-α is highly dependent on its downstream adaptor protein, TNF receptor-associated factor 2 (TRAF2). Upon binding of TNF ligands to TNF receptor type 1/type 2 (TNFR1/2, i.e., TNFRSF1A/TNFRSF1B), TRAF2 is recruited to the receptor complex, subsequently activating key signaling pathways such as canonical NF-κB and JNK/p38 [19, 20]. TRAF2 exerts its E3 ubiquitin ligase activity through the N-terminal RING domain, catalyzing K63-linked polyubiquitination of downstream substrates, while its C-terminal TRAF domain primarily serves a scaffolding function [21]. Beyond TNF signaling, studies have also revealed that TRAF2 participates in NOD-like receptor-mediated NF-κB activation, RIG-I-like receptor antiviral responses, and signal transduction of various cytokine receptors [22, 23]. In normal cells, the activity of TRAF2 is tightly constrained by mechanisms involving deubiquitinating enzymes such as A20 and CYLD [24, 25]. Multiple studies indicate that TRAF2 is upregulated in various cancers, including glioma, and can serve as a prognostic biomarker [26]. In tumor cells, elevated TRAF2 protein levels and dysregulated E3 ligase activity are closely associated with persistent NF-κB activation, malignant progression, and poor patient prognosis [27, 28]. Based on this evidence, we propose a key scientific hypothesis: in GBM, low-dose TNF-α activates TRAF2, which then functions as an E3 ubiquitin ligase to catalyze the ubiquitination of FASN, thereby enhancing its stability and ultimately driving tumor progression.

To validate this hypothesis, our study systematically elucidated the mechanism by which low-dose TNF-α regulates lipid metabolism and promotes malignant progression of GBM through the TRAF2-FASN axis. We found that low-dose TNF-α, in a TNFR-dependent manner, induces the interaction between TRAF2 and FASN. TRAF2 subsequently catalyzes K63-linked polyubiquitination of FASN, enhancing its protein stability, promoting lipid synthesis, and ultimately accelerating tumor growth. Through virtual screening, we identified a small-molecule compound, Jionoside B1, which targets the RING domain of TRAF2. This compound effectively inhibits TRAF2-mediated K63-linked polyubiquitination of FASN, thereby reducing FASN stability, and demonstrates significant anti-tumor effects both in vitro and in vivo. This study reveals for the first time the central role of the TNF-α-TRAF2-FASN regulatory axis in GBM lipid metabolism, providing new insights into the inflammatory microenvironment of GBM and offering a potential novel therapeutic strategy for this lethal disease.

## Results

### Low-dose TNF-α promotes malignant progression and lipid metabolism in GBM cells

Since GBM tumor cells themselves typically do not secrete or only secrete very low levels of TNF-α, the TNF-α in their microenvironment primarily originates from infiltrating immune cells [29, 30]. Therefore, the key for GBM cells to perceive TNF-α signaling lies in the expression and function of its surface receptors [31, 32]. To investigate the role of TNF-α in GBM, we first analyzed the expression of its receptors, TNFRSF1A and TNFRSF1B. Analysis of the TCGA database via GEPIA2 showed that the mRNA expression level of TNFRSF1A was significantly upregulated in GBM tumor samples(T) compared to normal tissue(N) (Figure S1A). Further validation using multiple glioma datasets revealed that TNFRSF1A is highly expressed across different glioma grades and subtypes, with the most significant expression observed in glioblastoma (Figure S1B). Survival analysis indicated that high TNFRSF1A expression is associated with shorter overall survival in GBM patients, suggesting its potential role in promoting tumor progression (Figure S1C). To further clarify the expression pattern and clinical significance of TNFRSF1A in glioblastoma, we performed immunohistochemistry on 53 GBM tissue samples and 4 normal brain tissue samples. The results showed strong positive staining for TNFRSF1A in both the cytoplasm and cell membrane of GBM tissues (Figure S1D). Quantitative analysis further confirmed that the expression level of TNFRSF1A was significantly higher in GBM tissues compared to normal brain tissues (Figure S1E). For survival analysis, the 53 patients with complete survival information were divided into two groups based on the median TNFRSF1A expression level in GBM samples. Patients with high TNFRSF1A expression showed a significantly worse clinical prognosis, and this association remained after adjusting for factors such as age and gender (Figure S1F and Supplemental Data 1). Similarly, analysis of TNFRSF1B revealed a consistent trend (Figure S2A–F and Supplemental Data 2). These results indicate that both main receptors for TNF-α are aberrantly overexpressed in GBM, and their expression levels are closely associated with poorer clinical outcomes in patients, collectively suggesting that TNF-α receptor signaling pathways may play an important role in GBM progression.

**Fig. 1:**
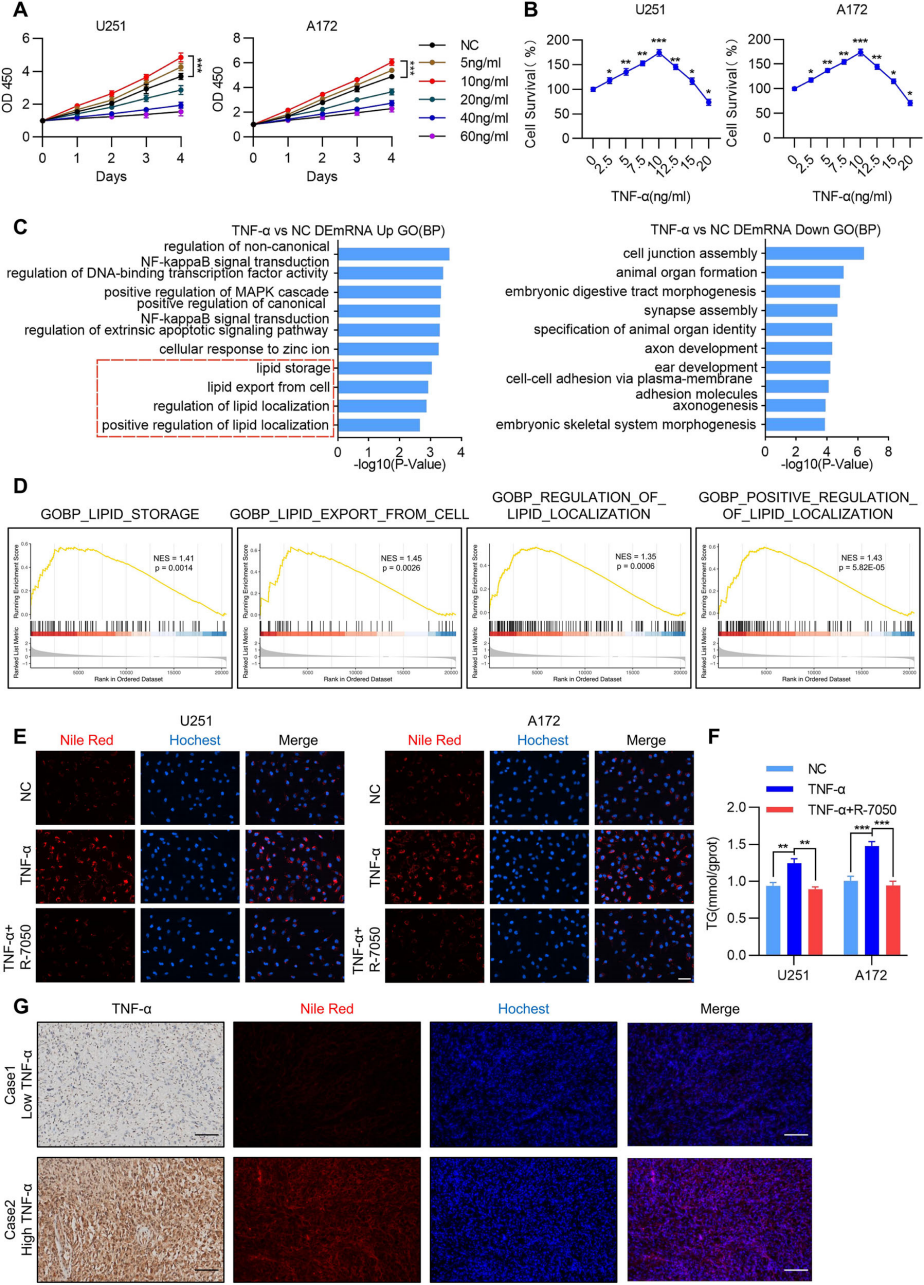
Low-dose TNF-α promotes malignant progression and lipid metabolism in GBM cells. **A** The effects of different concentrations of TNF-α (5, 10, 20, 40, 60 ng/mL) on the proliferation of U251 and A172 cells were determined by CCK-8 assay (*n* = 5, Student’s *t*-test, ***p < 0.001). **B** The cytotoxic effects of TNF-α within the concentration range of 2.5–20 ng/mL on U251 and A172 cells were assessed. Cell viability was measured by CCK-8 assay after 48-hour treatment with the corresponding concentrations of TNF-α(*n* = 5, Student’s *t*-test, *p < 0.05,**p < 0.01, ***p < 0.001). **C** GO enrichment analysis of significantly up- and down-regulated genes (|log_2_FC | ≥ 1, p-adj ≤ 0.05) in U251 cells treated with TNF-α (10 ng/mL) for 24 h. Bar charts show the most significantly enriched GO biological process terms for the up- and down-regulated differentially expressed genes (DEGs) in the TNF-α group versus the control group. **D** Gene Set Enrichment Analysis (GSEA) of TNF-related signaling in the TCGA-GBM database. NES, normalized enrichment score. **E** The effect of the TNFR inhibitor R-7050 on lipid droplet formation in U251 and A172 cells was detected by Nile Red staining after 24 h stimulation with TNF-α (10 ng/mL) (*n* = 5). Scale bar, 50 μm. **F** The effect of the TNFR inhibitor R-7050 on triglyceride (TG) synthesis in U251 and A172 cells was detected using a biochemical assay kit after 24-hour stimulation with TNF-α (10 ng/mL). Quantitative results are presented as a histogram (*n* = 5, Student’s *t*-test, **p < 0.01, ***p < 0.001). **G** Nile Red staining was performed on glioma tissue specimens known to have high or low endogenous TNF-α levels (*n* = 5). Scale bar, 100 μm.

Building on this, we investigated the effects of different doses of TNF-α on the malignant phenotype of GBM cells. CCK-8 assays assessing the impact of various TNF-α concentrations (5, 10, 20, 40, 60 ng/mL) on the proliferation of U251 and A172 cells revealed that 10 ng/mL TNF-α significantly increased cell viability, whereas other concentrations had no such effect or even inhibited proliferation (Fig. 1A). Further dose-response analysis revealed that within the concentration range of 2.5–20 ng/mL, TNF-α exhibited a biphasic effect on cell proliferation, shifting from promotion to inhibition. Specifically, 2.5 ng/mL TNF-α already showed a proliferative effect, which peaked at 10 ng/mL. Beyond this concentration, the promoting effect gradually diminished and eventually turned into inhibition. Based on these findings, 10 ng/mL was selected as the optimal concentration of TNF-α for subsequent experiments (Fig. 1B). Colony formation assays further confirmed that a 24 h treatment with 10 ng/mL TNF-α significantly enhanced the colony-forming ability of U251 and A172 cells (Figure S3A). EdU assays demonstrated that treatment with the same dose of TNF-α for 24 h markedly increased the proportion of EdU-positive cells, indicating that low-dose TNF-α promotes DNA replication and proliferation in GBM cells (Figure S3B). Next, we evaluated the impact of low-dose TNF-α on cell migration and invasion. Transwell assays showed that treatment with 10 ng/mL TNF-α for 24 h significantly enhanced the migration and invasion capabilities of U251 and A172 cells (Figure S3C, D). Similarly, ibidi wound healing assays revealed that TNF-α treatment promoted cell motility and accelerated wound closure (Figure S3E).

To elucidate the molecular mechanisms regulated by low-dose TNF-α, we performed transcriptome analysis of U251 cells treated with 10 ng/mL TNF-α. GO enrichment analysis showed that the upregulated differentially expressed genes in the TNF-α group were primarily enriched in lipid metabolic processes, and TNF-α was positively correlated with lipid storage, lipid export from cell, and regulation of lipid localization (Fig. 1C). As TNF represents a protein superfamily, and TNF-α—the earliest-discovered and key member mainly produced by macrophages that plays a central role in inflammation and immune responses—we further performed GSEA analysis in the TCGA-GBM database to explore the biological processes influenced by the TNF superfamily [33, 34]. The results demonstrated that TNF-associated signaling was closely linked to pathological pathways in glioblastoma such as lipid storage, lipid export from cell, and regulation of lipid localization, which aligns with the earlier sequencing results in U251 cells, further supporting the important role of TNF-α in regulating lipid metabolism (Fig. 1D). These findings suggest that lipid metabolism may mediate the tumor-promoting effects of TNF-α. Nile Red staining revealed that 10 ng/mL TNF-α treatment significantly promoted lipid droplet accumulation in GBM cells, an effect reversed by the TNFR inhibitor R-7050 (Fig. 1E). Biochemical assays showed that TNF-α-induced elevation of intracellular triglyceride levels could also be suppressed by R-7050, indicating the dependence of this process on TNFR signaling (Fig. 1F). Finally, analysis of clinical glioma tissues showed that tumor samples with higher endogenous TNF-α levels exhibited more pronounced lipid droplet accumulation (Fig. 1G). Together, these results demonstrate that low-dose TNF-α promotes GBM cell proliferation, migration, invasion, and lipid metabolism—particularly lipid droplet formation and triglyceride synthesis.

### Low-dose TNF-α regulates the malignant progression and lipid metabolism of GBM through TRAF2

Building upon previous experiments confirming that low-dose TNF-α significantly promotes malignant phenotypes and lipid metabolism in GBM cells through the TNFR signaling axis, we further focused on its key downstream adaptor protein, TRAF2, as our research subject. TRAF2 is the central E3 ubiquitin ligase in the TNFR signaling pathway, capable of directly activating multiple pro-proliferation and pro-survival pathways such as NF-κB and MAPK [35]. However, whether TRAF2 mediates the tumor-promoting effects of TNF-α in glioma, and whether there is a causal link between its potential oncogenic mechanisms and lipid metabolism, remain poorly understood. Therefore, this study, using TRAF2 as an entry point, aims to elucidate its key functions and molecular mechanisms in TNF-α-induced malignant progression and lipid metabolism in GBM.

**Fig. 2:**
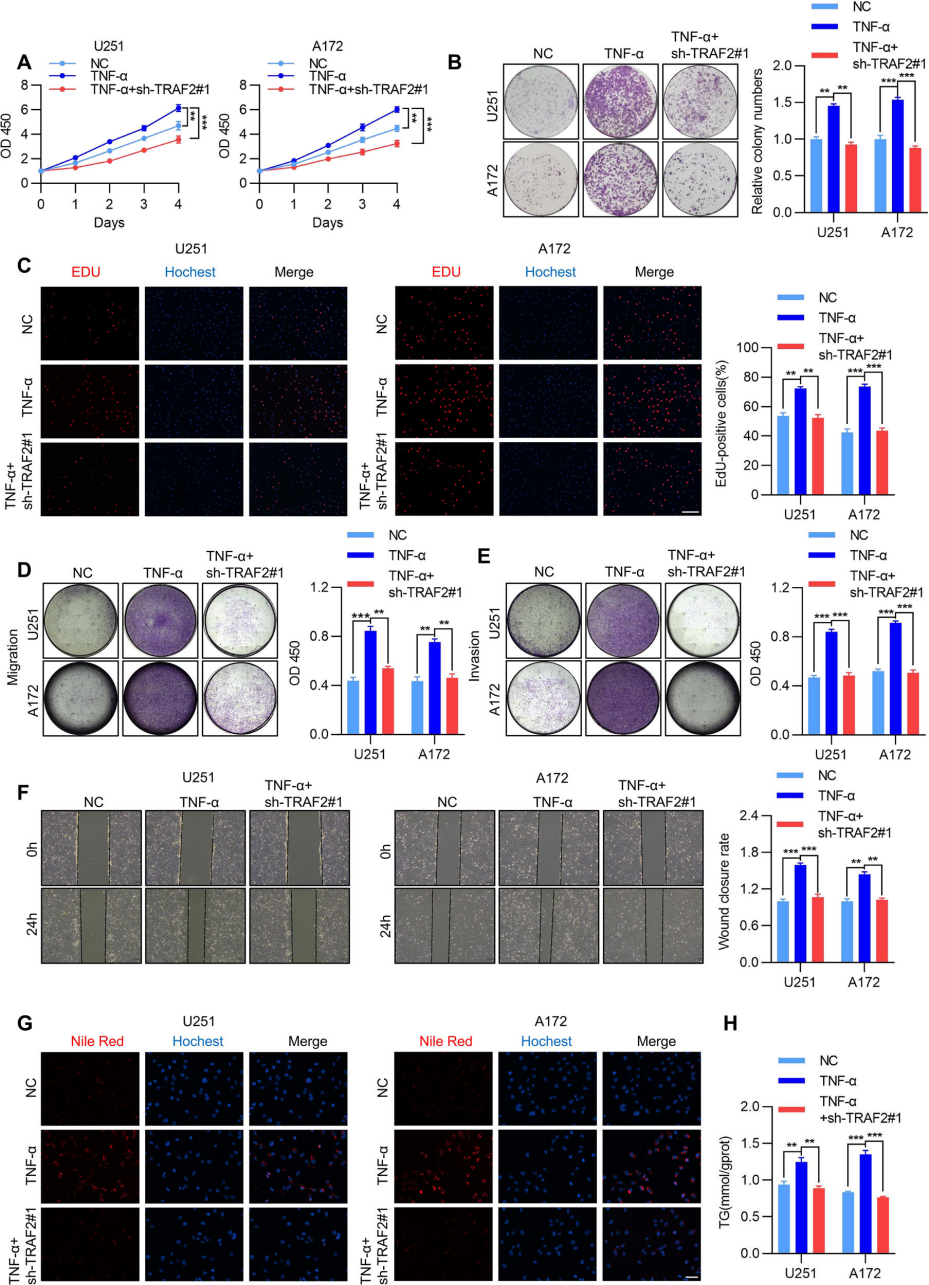
Low-dose TNF-α regulates the malignant progression and lipid metabolism of GBM through TRAF2. **A** The effect of TRAF2 knockdown on the proliferation of U251 and A172 cells after TNF-α stimulation was detected by CCK-8 assay (*n* = 5, Student’s *t*-test, **p < 0.01, ***p < 0.001). **B** The effect of TRAF2 knockdown on the clonogenic ability of U251 and A172 cells after TNF-α stimulation was detected by colony formation assay. Quantitative results are presented as a histogram (*n* = 5, Student’s *t*-test, **p < 0.01, ***p < 0.001). **C** The effect of TRAF2 knockdown on DNA replication and proliferation of U251 and A172 cells after TNF-α stimulation was analyzed by EdU assay, expressed as the percentage of EdU-positive cells (red) relative to the total number of Hoechst-stained cells (blue). Scale bar, 200 µm. Quantitative results are presented as a histogram (*n* = 5, Student’s *t*-test, **p < 0.01, ***p < 0.001). **D**, **E** The effects of TRAF2 knockdown on the migration and invasion abilities of U251 and A172 cells after TNF-α stimulation were evaluated using Transwell assays. Quantitative results are presented as histograms (*n* = 5, Student’s *t*-test, **p < 0.01, ***p < 0.001). **F** The effect of TRAF2 knockdown on the wound closure capacity of U251 and A172 cells after TNF-α stimulation was assessed using the ibidi wound healing assay. Quantitative results are presented as a histogram (*n* = 5, Student’s *t*-test, **p < 0.01, ***p < 0.001). **G** The effect of TRAF2 knockdown on lipid droplet formation ability in U251 and A172 cells after TNF-α stimulation was detected by Nile Red staining (*n* = 5). Scale bar, 50 μm. **H** The effect of TRAF2 knockdown on triglyceride (TG) synthesis capacity in U251 and A172 cells after TNF-α stimulation was measured using a biochemical kit. Quantitative results are presented as a histogram (*n* = 5, Student’s *t*-test, **p < 0.01, ***p < 0.001).

To investigate the role of TRAF2 in low-dose TNF-α-promoted malignant progression of glioblastoma, we constructed TRAF2-knockdown U251 and A172 cell models and performed a series of functional assays under TNF-α (10 ng/mL) stimulation. CCK-8 assays demonstrated that TRAF2 knockdown significantly inhibited the TNF-α-induced proliferation promotion in both U251 and A172 cells (Fig. 2A). Colony formation assays further revealed that TRAF2 knockdown effectively attenuated the enhancing effect of TNF-α on cell clonogenic ability (Fig. 2B). EdU staining analysis showed that TRAF2 knockdown markedly suppressed TNF-α-induced DNA replication and cell proliferation (Fig. 2C). Regarding cell migration and invasion, Transwell assay results indicated that TRAF2 knockdown significantly inhibited the TNF-α-induced enhancement of migratory and invasive capacities in U251 and A172 cells (Fig. 2D, E). The ibidi wound healing assay also confirmed that TRAF2 knockdown significantly reduced the rate of wound closure upon TNF-α stimulation (Fig. 2F). To further investigate the role of TRAF2 in TNF-α-regulated lipid metabolism, we performed Nile Red staining, which showed that TRAF2 knockdown significantly suppressed TNF-α-induced lipid droplet accumulation (Fig. 2G). Measurement of intracellular triglyceride (TG) levels using a biochemical assay kit revealed that TRAF2 knockdown markedly reversed the TNF-α-induced promotion of TG synthesis (Fig. 2H). These results collectively indicate that TRAF2 is a key signaling molecule mediating the low-dose TNF-α-induced promotion of GBM cell proliferation, migration, invasion, and lipid metabolism.

### TRAF2 is an oncogene that regulates lipid metabolism in GBM

To elucidate the role of TRAF2 in GBM, we first examined its expression levels in GBM cells. Western blot and RT-qPCR analyses revealed that compared to normal human astrocytes (NHA) and the low-grade glioma (LGG) cell line Sw1783, TRAF2 expression was significantly upregulated in multiple GBM cell lines (U87, A172, U251), with the highest levels observed in A172 and U251 cells (Figure S4A). Given the high expression of TRAF2 in A172 and U251 cells, this study selected these two cell lines as the experimental models. To further investigate the biological functions of TRAF2, we established stable TRAF2-knockdown and TRAF2-overexpressing cell lines in U251 and A172 cells. Western blot and RT-qPCR results confirmed that shRNA effectively reduced both TRAF2 protein and mRNA levels, while the overexpression vector significantly increased its expression (Figure S4B, C).

**Fig. 3:**
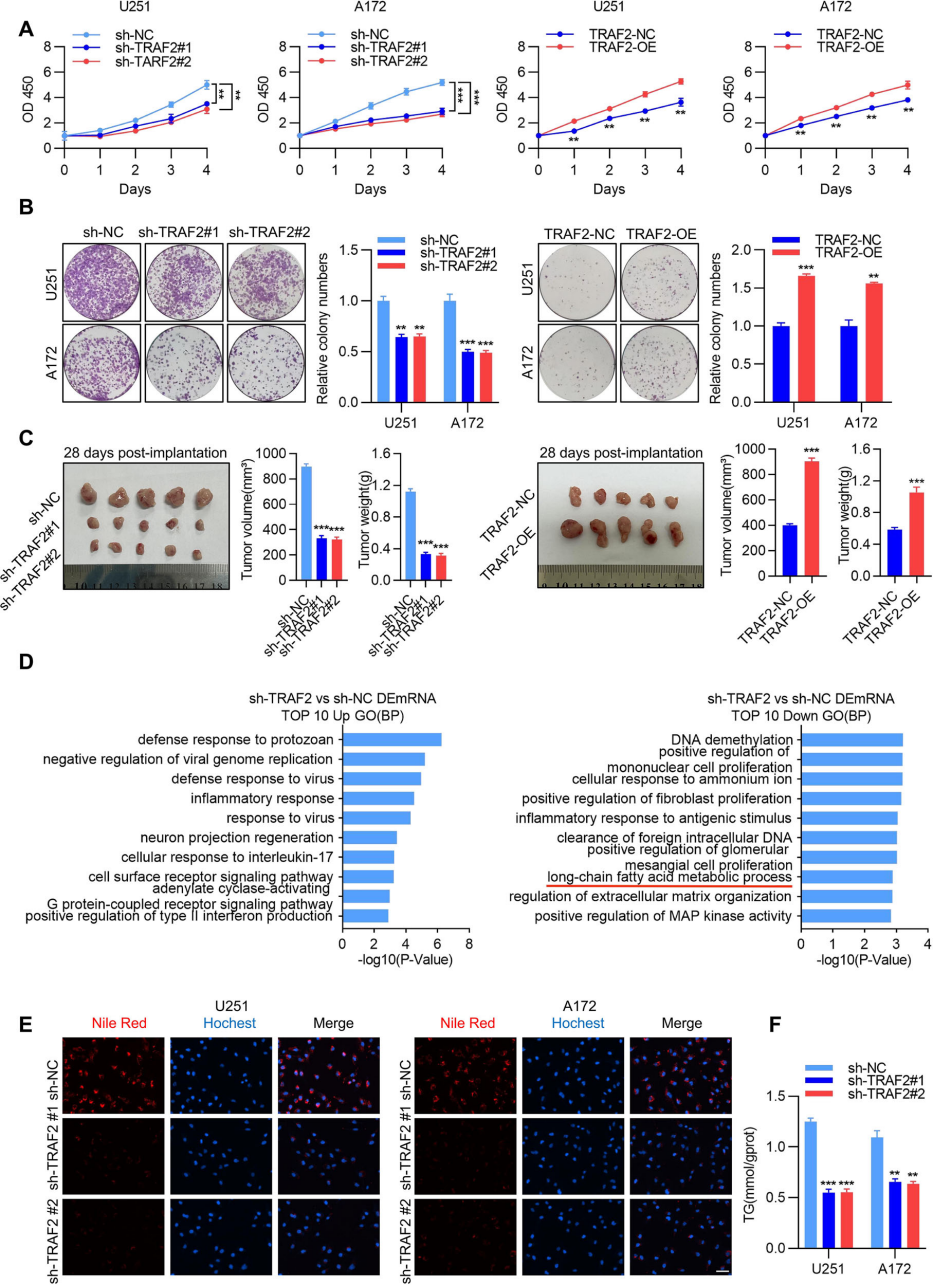
TRAF2 is an oncogene that regulates lipid metabolism in GBM. **A** The effects of TRAF2 knockdown or overexpression on the proliferation of U251 and A172 cells were measured by CCK-8 assay, respectively. Quantitative results are presented as histograms (*n* = 5, Student’s *t*-test, **p < 0.01, ***p < 0.001). **B** The effects of TRAF2 knockdown or overexpression on the colony formation ability of U251 and A172 cells were determined by colony formation assay, respectively. Quantitative results are presented as histograms (*n* = 5, Student’s *t*-test, **p < 0.01, ***p < 0.001). **C** U251 cells with stable TRAF2 knockdown (sh-TRAF2#1, sh-TRAF2#2), TRAF2 overexpression (TRAF2-OE), and their corresponding controls (sh-NC, TRAF2-NC) were subcutaneously inoculated into nude mice. Mice were sacrificed 28 days post-inoculation, tumors were photographed, and their volume and weight were measured. Quantitative results are presented as histograms (*n* = 5, Student’s *t*-test, **p < 0.01, ***p < 0.001). **D** GO enrichment analysis was performed on significantly up- and down-regulated genes (|log_2_FC | > 1, q-value < 0.05) in TRAF2-knockdown U251 cells. The bar charts display the top 10 most significantly enriched GO biological process terms based on the significantly altered genes. **E** The effect of TRAF2 knockdown on lipid droplet formation in U251 and A172 cells was detected by Nile Red staining after TRAF2 knockdown (*n* = 5). Scale bar, 50 μm. **F** The effect of TRAF2 knockdown on triglyceride (TG) synthesis capacity in U251 and A172 cells was measured using a biochemical kit after TRAF2 knockdown. Quantitative results are presented as a histogram (*n* = 5, Student’s *t*-test, **p < 0.01, ***p < 0.001).

Subsequently, using CCK-8 assays, colony formation assays, and EdU staining, we found that TRAF2 knockdown significantly inhibited the proliferative capacity, clonogenic ability, and DNA replication activity of U251 and A172 cells. Conversely, TRAF2 overexpression markedly promoted these malignant phenotypes (Fig. 3A, B and Figure S5A). Simultaneously, TRAF2 also significantly influenced the migratory and invasive behaviors of GBM cells. TRAF2 knockdown substantially weakened cell migration and invasion capabilities and delayed the wound closure process (ibidi assay), whereas its overexpression significantly enhanced these properties (Figure S5B–D). Furthermore, in vivo experiments confirmed that, compared to the control group, TRAF2 knockdown significantly inhibited the growth of subcutaneous xenograft tumors in nude mice, as evidenced by significantly reduced tumor volume and weight. In contrast, TRAF2 overexpression resulted in larger and heavier tumors (Fig. 3C).

Based on the previous finding that low-dose TNF-α promotes lipid metabolism, we hypothesized that TRAF2 might be involved in regulating this process. Transcriptome sequencing and GO enrichment analysis of TRAF2-knockdown U251 cells revealed that the differentially expressed genes were significantly enriched in the long-chain fatty acid metabolic process, which was notably downregulated upon TRAF2 knockdown, suggesting that TRAF2 might promote tumorigenesis by regulating lipid metabolism (Fig. 3D). To further validate this hypothesis, we employed Nile Red staining to detect lipid droplet accumulation and found that TRAF2 knockdown significantly reduced lipid droplet content in both U251 and A172 cells (Fig. 3E). Concurrently, measurement of triglyceride (TG) levels using a biochemical assay kit confirmed that TRAF2 knockdown significantly inhibited intracellular TG synthesis (Fig. 3F). In summary, these results demonstrate that TRAF2 not only acts as an oncogene regulating the proliferation, migration, and invasion of GBM cells but also promotes tumorigenesis and progression by modulating long-chain fatty acid metabolism and lipid droplet accumulation.

### TRAF2 interacts with FASN and upregulates its expression

**Fig. 4:**
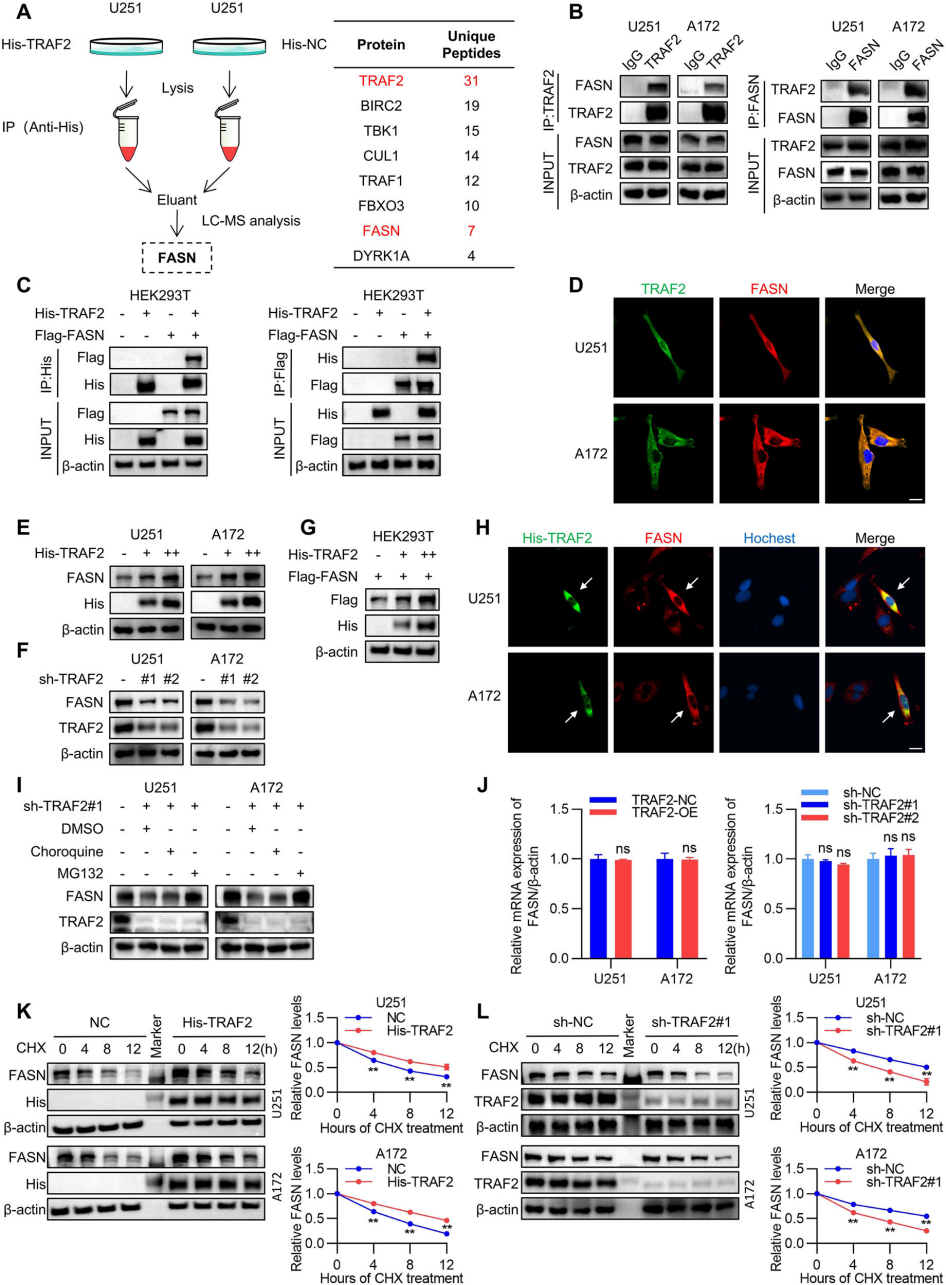
TRAF2 interacts with FASN and upregulates its expression. **A** Flowchart of mass spectrometry analysis to identify proteins interacting with TRAF2 in U251 cells. **B** The interaction between endogenous TRAF2 and FASN was verified in U251 and A172 cells by co-immunoprecipitation (Co-IP) assay. **C** Co-immunoprecipitation (Co-IP) assay was performed to detect the interaction between TRAF2 and FASN in HEK293T cells co-transfected with His-TRAF2 and Flag-FASN. **D** IF staining analysis confirmed the cytoplasmic co-localization of TRAF2 and FASN proteins in U251 and A172 cells. Endogenous TRAF2 and FASN were stained with anti-TRAF2 (green) and anti-FASN (red) antibodies, respectively. Scale bar, 20 μm. **E** Western blot analysis of FASN protein expression in U251 and A172 cells overexpressing TRAF2. **F** Western blot analysis of FASN protein expression in U251 and A172 cells with TRAF2 knockdown. **G** FASN protein expression was assessed by Western blot in HEK293T cells transfected with Flag-FASN and increasing doses of His-TRAF2 plasmid. **H** U251 and A172 cells were transiently transfected to express His-TRAF2. Immunofluorescence analysis was performed using anti-His-TRAF2 (green) and anti-FASN (red) antibodies. Nuclei were stained with Hoechst (blue). White arrows indicate His-TRAF2-positive cells. Scale bar, 30 μm. **I** Western blot analysis of lysates from U251 and A172 cells with TRAF2 knockdown treated with DMSO, MG132 (10 μM), or chloroquine (25 μM). **J** RT-qPCR analysis of FASN mRNA expression levels in U251 and A172 cells with TRAF2 overexpression or knockdown (*n* = 5, Student’s *t*-test, ns not significant). **K** Cycloheximide (CHX, 100 μg/ml) was used to treat U251 and A172 cells overexpressing TRAF2 to block new protein synthesis and measure the protein half-life of FASN. Protein stability was analyzed using ImageJ (*n* = 5, Student’s *t*-test, **p < 0.01). **L** Cycloheximide (CHX, 100 μg/ml) was used to treat U251 and A172 cells with TRAF2 knockdown to block new protein synthesis and measure the protein half-life of FASN. Protein stability was analyzed using ImageJ (*n* = 5, Student’s *t*-test, **p < 0.01).

To elucidate the molecular mechanism by which TRAF2 regulates long-chain fatty acid metabolism, we performed LC-MS/MS screening in U251 cells stably expressing His-TRAF2 and successfully identified Fatty Acid Synthase (FASN) as a potential interacting target protein of TRAF2 (Fig. 4A and Supplementary Table 5). Subsequently, co-immunoprecipitation (Co-IP) assays confirmed the endogenous interaction between TRAF2 and FASN in both U251 and A172 cells (Fig. 4B). This interaction was further validated under exogenous expression conditions in HEK293T cells co-transfected with His-TRAF2 and Flag-FASN (Fig. 4C). Immunofluorescence staining demonstrated the cytoplasmic co-localization of TRAF2 and FASN in U251 and A172 cells (Fig. 4D). To identify the structural basis underlying the TRAF2-FASN interaction, we constructed a series of truncated proteins based on the functional domains of TRAF2 (RING, Zinc Fingers, and TRAF domain) (Figure S6A). Domain mapping experiments confirmed that the TRAF domain is the critical region mediating its interaction with FASN (Figure S6B, C).

To elucidate the regulatory role of TRAF2 on FASN expression, we found that overexpression of TRAF2 specifically upregulated FASN protein levels in U251 and A172 cells (Fig. 4E), while knockdown of TRAF2 reduced FASN protein expression (Fig. 4F). In HEK293T cells, exogenous TRAF2 promoted FASN protein expression in a dose-dependent manner (Fig. 4G). To further clarify whether the regulatory relationship between the two exhibits feedback properties, we examined changes in TRAF2 protein levels under conditions of FASN overexpression or knockdown. The results showed that neither overexpression nor knockdown of FASN significantly altered TRAF2 protein levels in U251 and A172 cells (Figure S6D, E), indicating that FASN does not feedback-regulate TRAF2 abundance, and the regulatory interaction in this model is unidirectional. Immunofluorescence analysis further demonstrated a significant enhancement of FASN signal in the cytoplasm of U251 and A172 cells after His-TRAF2 overexpression (Fig. 4H). Mechanistically, the decrease in FASN protein caused by TRAF2 knockdown could be reversed by the proteasome inhibitor MG132, but not by the lysosome inhibitor chloroquine (Fig. 4I), suggesting that TRAF2 regulates FASN stability via the proteasomal pathway. Notably, neither TRAF2 overexpression nor knockdown significantly affected FASN mRNA transcript levels (Fig. 4J). To further validate the effect of TRAF2 on FASN stability, CHX chase assays showed that TRAF2 overexpression significantly extended the half-life of FASN protein (Fig. 4K), whereas TRAF2 knockdown accelerated its degradation (Fig. 4L). These results indicate that TRAF2, through direct interaction with FASN, inhibits its degradation via the proteasomal pathway, thereby upregulating FASN protein expression at the post-translational level.

### TRAF2 mediates K63-linked ubiquitination of FASN

**Fig. 5:**
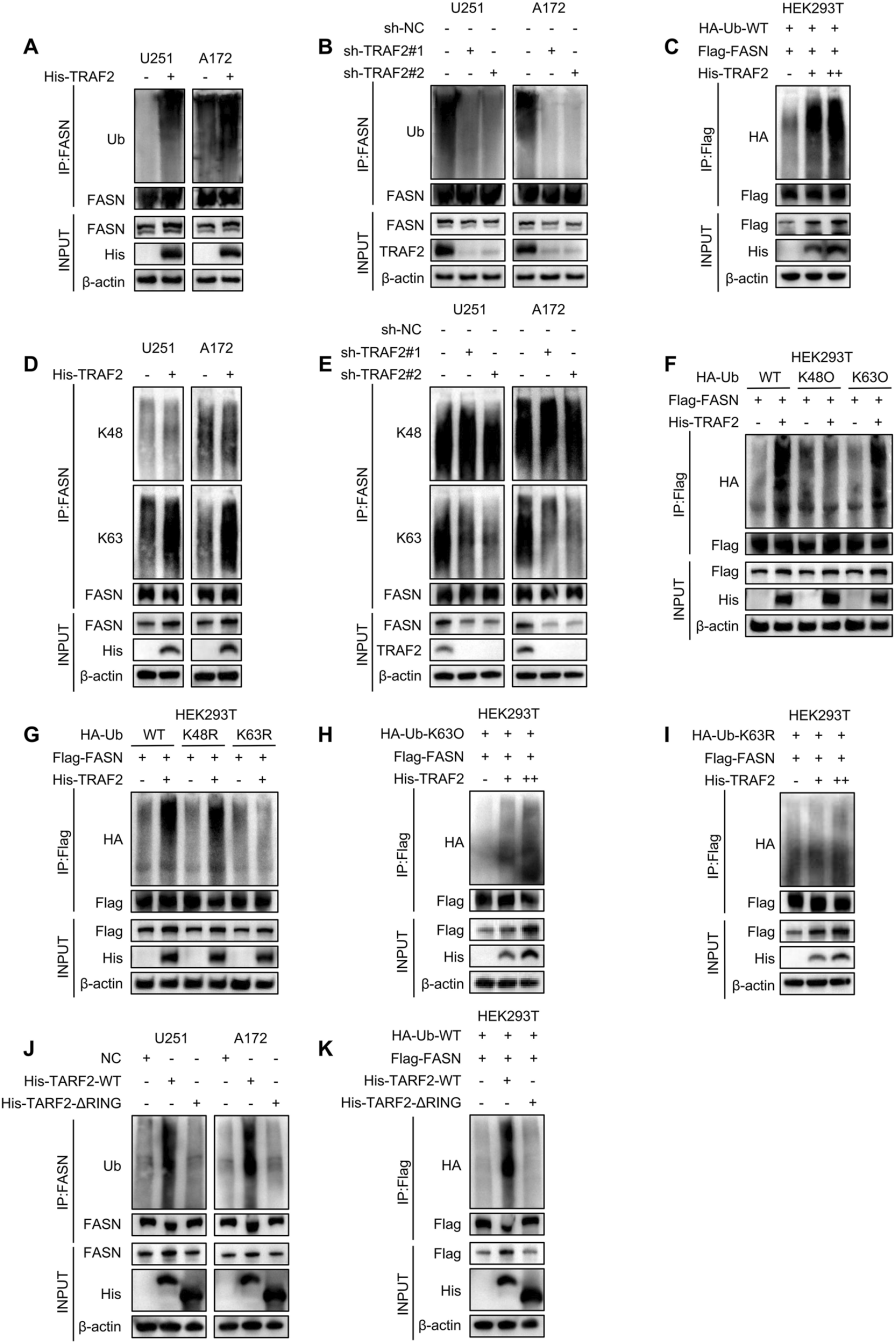
TRAF2 mediates K63-linked ubiquitination of FASN. **A** The amount of ubiquitin co-immunoprecipitated with FASN was detected in U251 and A172 cells overexpressing TRAF2. **B** The amount of ubiquitin co-immunoprecipitated with FASN was detected in U251 and A172 cells with TRAF2 knockdown. **C** HEK293T cells were transfected with HA-Ub, FLAG-FASN, and increasing doses of HIS-TRAF2 plasmids, followed by immunoprecipitation with an anti-FLAG antibody to detect the ubiquitination level of FASN. **D** The levels of K48- and K63-linked ubiquitination co-immunoprecipitated with FASN were detected in U251 and A172 cells overexpressing TRAF2. **E** The levels of K48- and K63-linked ubiquitination co-immunoprecipitated with FASN were detected in U251 and A172 cells with TRAF2 knockdown. **F** HEK293T cells were transfected with FLAG-FASN and HA-Ub (WT, K48-only, or K63-only) plasmids, with or without HIS-TRAF2, followed by immunoprecipitation with an anti-FLAG antibody to detect the ubiquitination level of FASN. **G** HEK293T cells were transfected with FLAG-FASN and HA-Ub (WT, K48R, or K63R) plasmids, with or without HIS-TRAF2, followed by immunoprecipitation with an anti-FLAG antibody to detect the ubiquitination level of FASN. **H** HEK293T cells were transfected with HA-Ub (K63-only), FLAG-FASN, and increasing doses of HIS-TRAF2 plasmids, followed by immunoprecipitation with an anti-FLAG antibody to detect the K63-linked ubiquitination level of FASN. **I** HEK293T cells were transfected with HA-Ub (K63R), FLAG-FASN, and increasing doses of HIS-TRAF2 plasmids, followed by immunoprecipitation with an anti-FLAG antibody to detect the ubiquitination level of FASN. **J** Full-length TRAF2 or its ΔRING mutant plasmids were co-transfected into U251 and A172 cells, followed by immunoprecipitation with an anti-FASN antibody to detect the ubiquitination level of FASN. **K** Analysis of the TRAF2 domain responsible for FASN ubiquitination. HA-Ub, FLAG-FASN, and full-length or truncated HIS-TRAF2 were co-transfected into HEK293T cells, followed by immunoprecipitation with an anti-FLAG antibody to detect the ubiquitination level of FASN.

TRAF2, functioning as an E3 ubiquitin ligase, can catalyze K63- or K48-linked polyubiquitination of substrate proteins [21, 36–39]. To elucidate the molecular mechanism by which TRAF2 regulates FASN protein stability, we investigated whether TRAF2 modulates FASN through ubiquitination. In TRAF2-overexpressing U251 and A172 cells, the level of ubiquitin co-immunoprecipitated with FASN was significantly increased (Fig. 5A). Conversely, in TRAF2-knockdown cells, the association between FASN and ubiquitin was markedly reduced (Fig. 5B). In HEK293T cells, exogenous expression of TRAF2 enhanced FASN polyubiquitination in a dose-dependent manner (Fig. 5C). To further identify the linkage type of ubiquitination, we found that TRAF2 overexpression specifically enhanced K63-linked ubiquitination of FASN, while it had no significant effect on K48-linked ubiquitination (Fig. 5D). Correspondingly, TRAF2 knockdown primarily suppressed K63-linked ubiquitination of FASN (Fig. 5E). To verify this specificity, we co-transfected HEK293T cells with either wild-type or mutant ubiquitin vectors. The results showed that TRAF2 promoted FASN ubiquitination only when the K63 linkage-specific ubiquitin (Ub-K63-only) was used, with no effect observed with the K48 linkage-specific ubiquitin (Ub-K48-only) (Fig. 5F). When the lysine 63 residue in ubiquitin was mutated (Ub-K63R), the enhancing effect of TRAF2 on FASN ubiquitination was completely abolished. In contrast, under the background of a lysine 48 mutation (Ub-K48R), TRAF2 still significantly enhanced FASN ubiquitination, indicating that TRAF2 primarily mediates K63-linked polyubiquitination of FASN (Fig. 5G). Dose-dependent experiments further confirmed that TRAF2 enhanced FASN ubiquitination in a dose-dependent manner only when K63 linkage-specific ubiquitin was present, with no such effect observed in the presence of the K63-mutant ubiquitin (Fig. 5H, I). Previous studies have revealed that TRAF2 utilizes its RING domain to mediate ubiquitination of substrate proteins [21, 37]. To identify the critical domain of TRAF2 mediating this process, we compared the function of full-length TRAF2 with a ΔRING mutant. The results demonstrated that full-length TRAF2 significantly induced FASN ubiquitination, whereas the RING domain-deleted mutant completely lost this ability (Fig. 5J). Further experiments with truncated constructs confirmed that the RING domain of TRAF2 is essential for promoting FASN ubiquitination (Fig. 5K). These results collectively indicate that TRAF2, via its RING domain, specifically mediates K63-linked polyubiquitination of FASN, thereby stabilizing the FASN protein.

### TNF-α regulates FASN through TRAF2

**Fig. 6:**
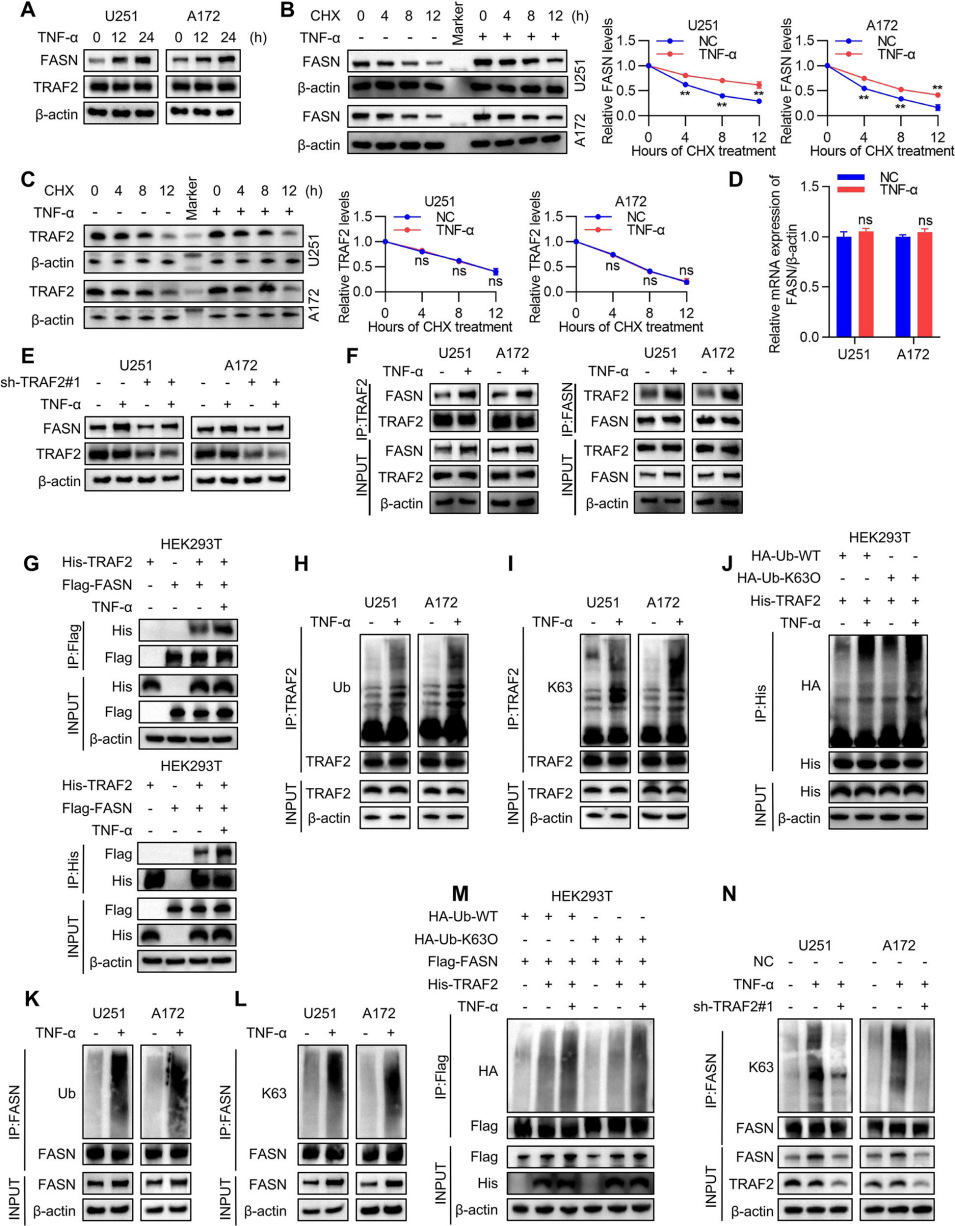
TNF-α regulates FASN through TRAF2. **A** U251 and A172 cells were treated with TNF-α (10 ng/mL) for indicated durations, and endogenous FASN and TRAF2 protein levels were analyzed by Western blot. **B** U251 and A172 cells stimulated with TNF-α (10 ng/mL) were treated with cycloheximide (CHX, 100 μg/mL) to block new protein synthesis. The protein half-life of FASN was measured and protein stability was analyzed using ImageJ (*n* = 5, Student’s *t*-test, **p < 0.01). **C** U251 and A172 cells stimulated with TNF-α (10 ng/mL) were treated with cycloheximide (CHX, 100 μg/mL) to block new protein synthesis. The protein half-life of TRAF2 was measured and protein stability was analyzed using ImageJ (*n* = 5, Student’s *t*-test, ns: not significant). **D** RT-qPCR analysis of FASN mRNA expression levels in U251 and A172 cells treated with TNF-α (10 ng/mL) for 24 h (*n* = 5, Student’s *t*-test, ns: not significant). **E** Endogenous FASN and TRAF2 protein levels were detected by Western blot in TRAF2-knockdown U251 and A172 cells re-exposed to TNF-α (10 ng/mL) stimulation. **F** Co-immunoprecipitation analysis of TRAF2 and FASN in U251 and A172 cells after stimulation with TNF-α (10 ng/mL) for indicated durations. **G** HEK293T cells co-transfected with HIS-TRAF2 and FLAG-FASN plasmids were treated with TNF-α (10 ng/mL) for indicated durations. Co-immunoprecipitation (Co-IP) was performed to assess the effect of TNF-α on the TRAF2-FASN interaction. **H**, **I** Levels of total ubiquitination and K63-linked polyubiquitination co-immunoprecipitated with TRAF2 were detected in U251 and A172 cells stimulated with TNF-α (10 ng/mL). **J** HEK293T cells co-transfected with HIS-TRAF2 and HA-Ub (WT or K63-only) plasmids were treated with or without TNF-α (10 ng/mL) stimulation. Immunoprecipitation was performed using an anti-HIS antibody to detect TRAF2 ubiquitination levels. **K**, **L** Levels of total ubiquitination and K63-linked polyubiquitination co-immunoprecipitated with FASN were detected in U251 and A172 cells stimulated with TNF-α (10 ng/mL). **M** HEK293T cells co-transfected with FLAG-FASN, HA-Ub (WT or K63-only), and HIS-TRAF2 plasmids were treated with or without TNF-α (10 ng/mL) stimulation. Immunoprecipitation was performed using an anti-FLAG antibody to detect FASN ubiquitination levels. **N** TRAF2 knockdown abolished the TNF-α-induced upregulation of endogenous FASN ubiquitination and protein levels in U251 and A172 cells.

To determine whether TNF-α regulates FASN through TRAF2, we first examined the effect of TNF-α stimulation on FASN expression in GBM cells. Western blot results showed that TNF-α significantly upregulated FASN protein levels in a time-dependent manner in both U251 and A172 cells, while it had no significant effect on TRAF2 expression (Fig. 6A). To further investigate whether TNF-α affects protein stability, we performed a chase experiment using the protein synthesis inhibitor cycloheximide (CHX). The results demonstrated that TNF-α treatment significantly extended the half-life of FASN protein but did not affect TRAF2 stability (Fig. 6B, C). RT-qPCR analysis revealed no significant effect of TNF-α on FASN mRNA levels, suggesting that its regulation occurs at the post-transcriptional level (Fig. 6D). To further validate the key role of TRAF2 in the TNF-α signaling pathway, we found that TRAF2 knockdown completely blocked the TNF-α-induced upregulation of FASN, indicating that TRAF2 is a critical mediator of TNF-α-regulated FASN expression (Fig. 6E).

Next, we examined the effect of TNF-α on the interaction between TRAF2 and FASN by co-immunoprecipitation (Co-IP). In U251 and A172 cells, TNF-α stimulation enhanced the endogenous binding between TRAF2 and FASN (Fig. 6F). Similarly, in HEK293T cells co-expressing His-TRAF2 and Flag-FASN exogenously, TNF-α treatment also promoted the interaction between the two proteins, indicating that TNF-α strengthens the formation of the TRAF2-FASN complex (Fig. 6G).

Within the TNFR1 signaling complex, TRAF2 acts as a core scaffold protein. Its K63-linked polyubiquitination is a crucial step for recruiting and activating downstream signaling molecules such as cIAP1/2 and RIPK1. This process, in turn, catalyzes the assembly of K63-linked ubiquitin chain networks, cooperatively triggering the cascade activation of NF-κB and MAPK pathways [40, 41]. To further investigate its regulatory mechanism on FASN in TNF-α signaling, we examined the effect of TNF-α on the ubiquitination of both TRAF2 and FASN. In U251 and A172 cells, TNF-α treatment significantly enhanced the total ubiquitination and K63-linked polyubiquitination levels of TRAF2 (Fig. 6H, I). Overexpression of TRAF2 along with different ubiquitin mutants in HEK293T cells further confirmed that TNF-α specifically induces K63-linked ubiquitination of TRAF2 (Fig. 6J). Similarly, TNF-α also markedly increased the total ubiquitination and K63-linked ubiquitination levels of FASN (Fig. 6K, L). Furthermore, in the HEK293T system co-expressing TRAF2 and FASN, TRAF2 overexpression promoted K63-linked ubiquitination of FASN, and TNF-α stimulation further enhanced this effect (Fig. 6M). Finally, to clarify the necessity of TRAF2 in TNF-α-induced FASN ubiquitination and protein expression, we applied TNF-α treatment in TRAF2-knockdown cells. Co-IP and Western blot results showed that TNF-α stimulation significantly enhanced the ubiquitination of endogenous FASN and increased its protein level; however, upon TRAF2 depletion, TNF-α failed to induce the upregulation of FASN ubiquitination and expression (Fig. 6N). These results collectively demonstrate that TRAF2 is an essential factor for TNF-α signaling to regulate FASN ubiquitination modification and protein stability.

### Clinical association between TRAF2 and FASN

**Fig. 7:**
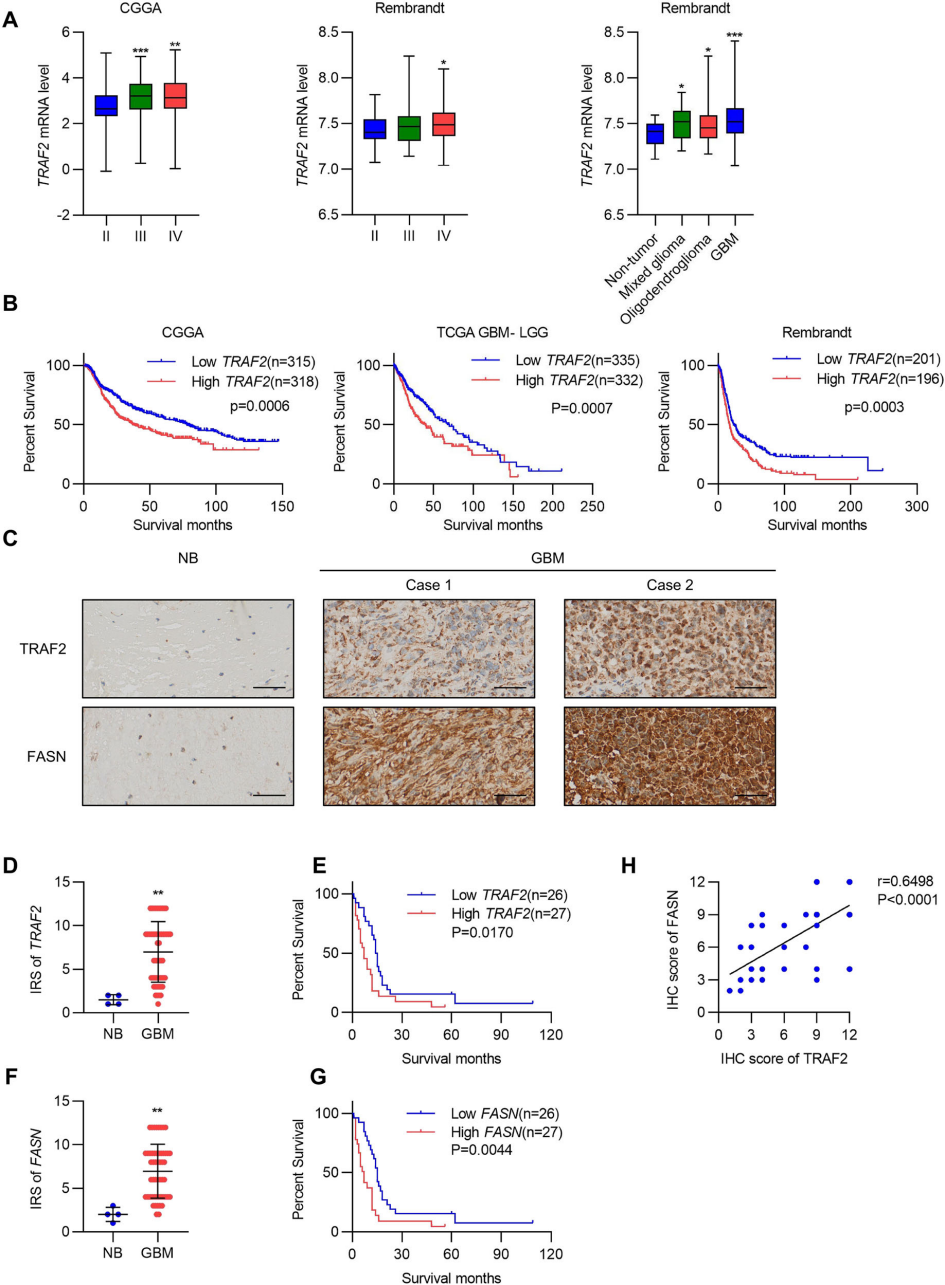
Clinical association between TRAF2 and FASN. **A** TRAF2 expression levels in glioma tissues from the CGGA and Rembrandt databases, including different WHO grades and different histological types (two-tailed Student’s *t*-test, *p < 0.05, **p < 0.01, ***p < 0.001). **B** Overall survival analysis based on TRAF2 mRNA expression levels in the specified GBM dataset (Kaplan-Meier survival test). **C** IHC staining of normal brain (NB) and primary glioblastoma (GBM) samples. Scale bar, 50 μm. **D** Compared with normal brain tissues, TRAF2 protein expression was generally elevated in GBM tissues (**p < 0.01, unpaired *t*-test). **E** Overall survival analysis based on TRAF2 expression levels in GBM patients (Kaplan-Meier survival analysis, Log-rank χ² = 5.695, *p* = 0.0170). Patients were grouped according to the median immunohistochemical reactivity score of TRAF2 in GBM tissues. **F** Compared with normal brain tissues, FASN protein expression was generally elevated in GBM tissues (**p < 0.01, unpaired *t*-test). **G** Overall survival analysis based on FASN expression levels in GBM patients (Kaplan-Meier survival analysis, Log-rank χ² = 8.101, *p* = 0.0044). Patients were grouped according to the median immunohistochemical reactivity score of FASN in GBM tissues. **H** To investigate the potential regulatory role of TRAF2 on FASN, the correlation between them was analyzed (*p*-value and *r*-value are indicated in the figure; Spearman correlation analysis was used).

To validate the clinical significance of the TRAF2-FASN signaling axis in glioma progression, we first analyzed the expression pattern of TRAF2 using public databases. Data from the CGGA and Rembrandt databases revealed that TRAF2 expression was significantly elevated in both low-grade gliomas and glioblastoma (GBM) compared to normal tissue, and its expression level progressively increased with advancing World Health Organization (WHO) tumor grade (Fig. 7A). Survival analysis further demonstrated that GBM patients with high TRAF2 expression had significantly shorter overall survival (Fig. 7B), suggesting TRAF2 serves as a negative prognostic indicator in GBM patients.

Through immunohistochemical (IHC) analysis of clinical tissue samples, we directly observed the expression of TRAF2 protein in GBM tissues (Fig. 7C). Quantitative analysis confirmed that TRAF2 protein expression levels were generally elevated in GBM tissues compared to normal brain tissues (Fig. 7D). Survival analysis based on TRAF2 protein expression in GBM patients also showed that high TRAF2 expression was significantly associated with poorer overall survival in patients, regardless of age and gender (Fig. 7E and Supplemental Data 3). Further analysis revealed that FASN protein expression in GBM tissues exhibited a similar trend, showing a significant increase compared to normal brain tissues (Fig. 7F), and high FASN expression similarly predicted shorter patient survival, regardless of age and gender (Fig. 7G and Supplemental Data 4). To investigate the potential regulatory relationship between TRAF2 and FASN, we analyzed their correlation in glioma tissues. The results showed a significant positive correlation between the expression levels of TRAF2 and FASN (Spearman r = 0.6498, *P* < 0.001) (Fig. 7H). Together, these clinical data indicate that TRAF2 and FASN expression are correlated in glioma, and the high expression of both is closely associated with poor patient prognosis, providing strong evidence for the clinical significance of the TNFα-TRAF2-FASN signaling axis in the development and progression of glioma.

### Small-molecule compounds block the TRAF2-FASN regulatory axis and inhibit the malignant phenotypes and lipid metabolism in GBM

**Fig. 8:**
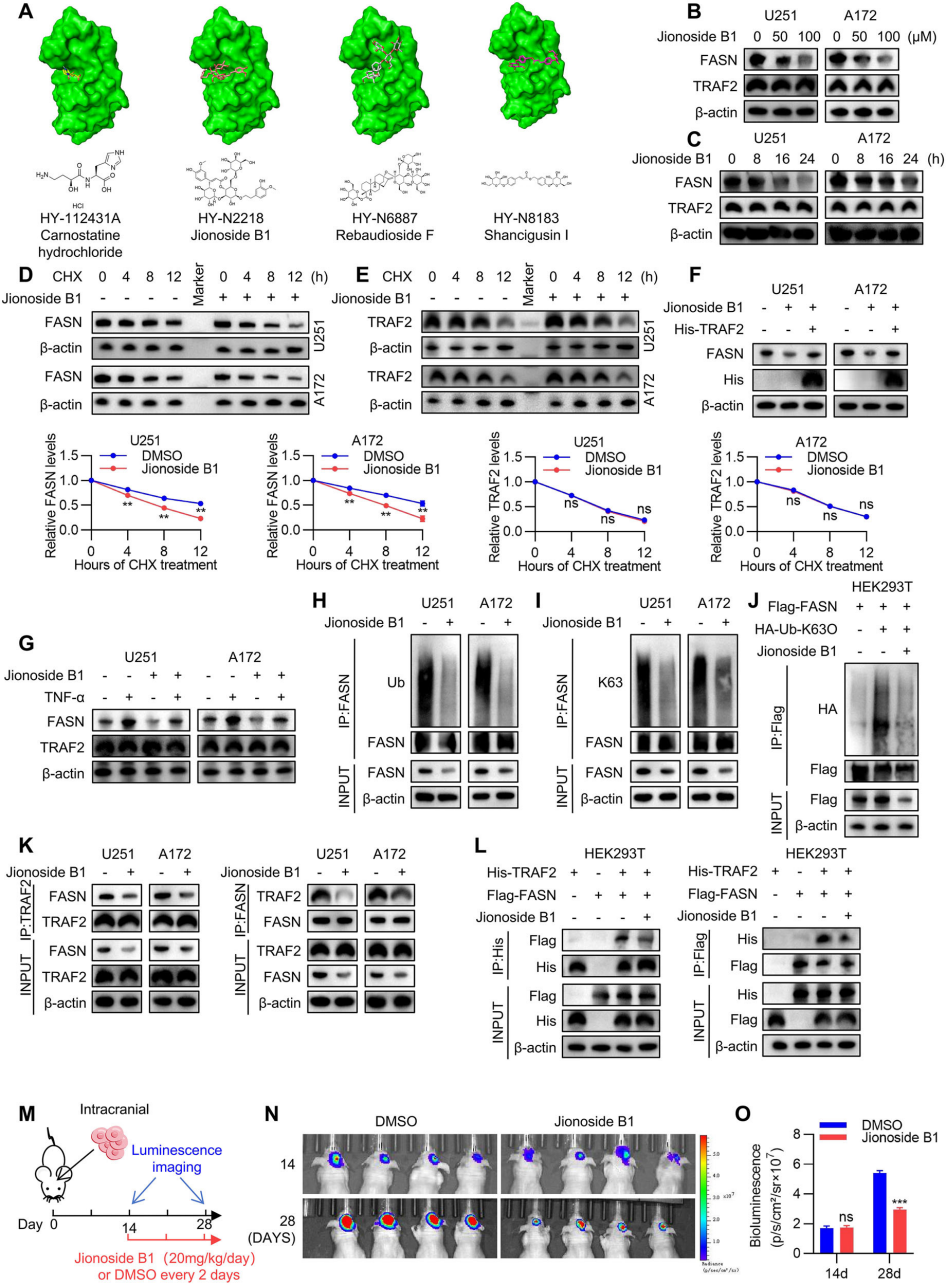
Small-molecule compounds block the TRAF2-FASN regulatory axis and inhibit the malignant phenotypes and lipid metabolism in GBM. **A** Simulated binding conformations of candidate small-molecule compounds obtained through virtual screening targeting the RING domain essential for TRAF2 catalytic activity. **B** Endogenous FASN and TRAF2 protein levels were analyzed by Western blot in U251 and A172 cells treated with gradient concentrations of Jionoside B1 for 24 h. **C** Endogenous FASN and TRAF2 protein levels were detected by Western blot in U251 and A172 cells treated with Jionoside B1 (100 μM) for indicated durations. **D** U251 and A172 cells stimulated with Jionoside B1 (100 μM) were treated with cycloheximide (CHX, 100 μg/mL) to block new protein synthesis. The protein half-life of FASN was measured and protein stability was analyzed using ImageJ (*n* = 5, Student’s *t*-test, **p < 0.01). **E** U251 and A172 cells stimulated with Jionoside B1 (100 μM) were treated with cycloheximide (CHX, 100 μg/mL) to block new protein synthesis. The protein half-life of TRAF2 was measured and protein stability was analyzed using ImageJ (*n* = 5, Student’s *t*-test, ns: not significant). **F**. Endogenous FASN protein levels were detected by Western blot in TRAF2-overexpressing U251 and A172 cells re-exposed to Jionoside B1 (100 μM) stimulation. **G** U251 and A172 cells were treated with TNF-α (10 ng/mL) alone or in combination with Jionoside B1 (100 μM) for 24 hours. **H**, **I** Levels of total ubiquitination and K63-linked polyubiquitination co-immunoprecipitated with FASN were detected in U251 and A172 cells stimulated with Jionoside B1 (100 μM). **J**. HEK293T cells co-transfected with FLAG-FASN and HA-Ub (K63-only) plasmids were treated with or without Jionoside B1 (100 μM) stimulation. Immunoprecipitation was performed using an anti-FLAG antibody to detect K63-linked ubiquitination of FASN. **K** Co-immunoprecipitation analysis of TRAF2 and FASN in U251 and A172 cells after stimulation with Jionoside B1 (100 μM) for indicated durations. **L** HEK293T cells co-transfected with HIS-TRAF2 and FLAG-FASN plasmids were treated with Jionoside B1 (100 μM) for indicated durations. Co-immunoprecipitation (Co-IP) was performed to assess the effect of Jionoside B1 on the TRAF2-FASN interaction. **M** Schematic diagram of in vivo evaluation of Jionoside B1’s tumor growth inhibition. Luciferase-expressing U251 cells were implanted into the brains of nude mice, and intraperitoneal injections of DMSO or Jionoside B1 (20 mg/kg/day) were administered every 2 days starting from day 14 post-surgery. Tumor volume was monitored by in vivo imaging on days 14 and 28 post-surgery. **N** In vivo imaging of orthotopic intracranial tumors. **O** Statistical results of tumor volumes (*n* = 4, Student’s *t*-test, ***p < 0.001).

Based on previous findings that TRAF2 exhibits oncoprotein properties and its RING domain is crucial for its tumor-promoting functions, we hypothesized that targeting this domain could provide a new therapeutic strategy for glioma. Through virtual screening of small-molecule compounds targeting the TRAF2 RING domain, we comprehensively evaluated binding affinity, ligand efficiency, and structural novelty, ultimately selecting four candidate compounds for validation (Fig. 8A). Among them, CCK-8 cytotoxicity assays showed that only Jionoside B1 dose-dependently inhibited the viability of U251 and A172 cells (Figure S7A), indicating its specific anti-tumor activity. To further evaluate the anti-tumor effects of Jionoside B1, we systematically analyzed its impact on the malignant phenotypes of glioma cells through colony formation assays, EdU assays, Transwell assays, and ibidi wound healing assays. The results demonstrated that Jionoside B1 significantly inhibited colony formation ability, DNA replication and proliferation, migration and invasion capabilities, as well as wound closure capacity (Figure S7B–F). At the metabolic level, Nile Red staining and triglyceride measurements showed that Jionoside B1 effectively blocked lipid droplet accumulation and triglyceride synthesis (Figure S7G, H), confirming its ability to inhibit tumor cell lipid metabolism.

Subsequently, we further investigated the mechanism of action of Jionoside B1. Western blot analysis revealed that this compound downregulated FASN protein levels in dose- and time-dependent manners, without affecting TRAF2 expression (Fig. 8B, C). CHX chase assays confirmed that Jionoside B1 significantly shortened the half-life of FASN protein but had no effect on TRAF2 stability (Fig. 8D, E). Rescue experiments showed that TRAF2 overexpression reversed the Jionoside B1-induced downregulation of FASN, and the compound effectively blocked TNF-α-induced FASN upregulation, suggesting it functions by targeting the TRAF2-FASN axis (Fig. 8F, G). At the molecular mechanism level, co-immunoprecipitation assays demonstrated that Jionoside B1 significantly inhibited both total ubiquitination and K63-linked polyubiquitination levels of FASN (Fig. 8H, I). In HEK293T cells, the compound similarly reduced K63-linked ubiquitination of FASN (Fig. 8J). Further studies found that Jionoside B1 effectively blocked both endogenous and exogenous interactions between TRAF2 and FASN (Fig. 8K, L). Finally, we evaluated the in vivo efficacy of Jionoside B1 using an orthotopic xenograft model (Fig. 8M). In vivo imaging showed that compared to the DMSO control group, the Jionoside B1-treated group exhibited significantly weakened tumor fluorescence signals, and tumor volume statistics confirmed that this compound effectively inhibited intracranial tumor growth (Fig. 8N, O). These results indicate that Jionoside B1, by targeting the TRAF2 RING domain, inhibits K63-linked polyubiquitination of FASN, disrupts the TRAF2-FASN interaction and FASN protein stability, thereby blocking fatty acid synthesis and tumor malignant progression, providing preclinical evidence for targeting the TRAF2-FASN axis in glioma therapy.

## Discussion

Although the role of TNF-α in inflammatory responses and its dual functions in tumor biology—both inhibiting and promoting tumor growth—have been widely recognized, the specific regulatory mechanisms through which it modulates lipid metabolism in glioblastoma (GBM) remain to be fully elucidated [42, 43]. Similarly, while TRAF2, as a key adaptor protein in TNFR signaling, is well-established for its role in activating NF-κB and MAPK pathways, whether this molecule directly participates in the regulation of lipid metabolism lacks systematic research evidence. Significant dysregulation of lipid metabolism is commonly observed in various cancers, including GBM, to support rapid tumor cell proliferation and survival; however, the upstream signaling mechanisms driving this phenomenon are still incompletely understood [44].

Addressing the questions above, this study for the first time uncovers a novel signaling axis connecting inflammatory signaling with lipid metabolism. We found that low-dose TNF-α promotes the proliferation, migration, invasion, and lipid accumulation in GBM cells. More importantly, we revealed a novel signaling axis: TNF-α, through its downstream effector TRAF2, directly regulates the stability of the FASN protein. We demonstrated that TRAF2, functioning as an E3 ubiquitin ligase, interacts with FASN and catalyzes its K63-linked polyubiquitination. This specific ubiquitination modification stabilizes the FASN protein, slows its degradation, and consequently leads to its accumulation. Ultimately, this process enhances de novo lipogenesis, manifested as increased lipid droplet formation and elevated triglyceride synthesis, thereby driving the malignant progression of GBM.

Our findings reveal multi-layered regulatory mechanisms within this pathway. First, we confirmed that the TNF-α-TRAF2-FASN axis operates at the post-translational level, as altering TRAF2 expression affects FASN protein levels but does not change its mRNA expression. Second, we identified the critical functional domains: the TRAF domain of TRAF2 is essential for its interaction with FASN, while its RING domain is indispensable for E3 ligase activity and K63-linked ubiquitination. Third, the clinical relevance of this axis is demonstrated by the positive correlation between TRAF2 and FASN protein expression in human GBM tissues, where high TRAF2 expression is closely associated with higher tumor grade and poor patient prognosis.

A significant highlight of this study lies in revealing a non-canonical mechanism regulating FASN stability. Although FASN is known to be regulated by transcription factors such as SREBP-1c and through allosteric modulation by metabolites, recent research has increasingly demonstrated that post-translational modifications, particularly ubiquitination, play a central role in the precise regulation of FASN stability [45–47]. This area is emerging as a new focus in metabolic regulation research. Our study adds TRAF2 to the list of E3 ligases known to regulate FASN stability, such as TRIM21 and the deubiquitinating enzyme USP14 [48–50]. Notably, recent studies have also identified TRIM65 as another crucial E3 ubiquitin ligase that directly binds to FASN and enhances its stability through K63-linked polyubiquitination, thereby promoting lipogenesis and tumorigenesis [51]. The coordinated action of pro-inflammatory cytokines (e.g., TNF-α) with the canonical signaling adaptor TRAF2 on this metabolic enzyme further illuminates the complex interplay between inflammatory signaling and aberrant metabolic activities in cancer cells.

At the therapeutic translation level, we discovered that the small-molecule inhibitor Jionoside B1, which selectively targets the RING domain of TRAF2, holds significant importance. Jionoside B1 effectively inhibits K63-linked ubiquitination of FASN, disrupts the TRAF2-FASN interaction, and accelerates FASN degradation, thereby suppressing lipogenesis, inhibiting malignant phenotypes in vitro, and impeding tumor growth in an orthotopic GBM mouse model. This provides strong proof-of-concept for targeting the TRAF2-FASN axis as a therapeutic strategy. The potential implications of targeting this axis extend beyond direct tumor growth inhibition. Given that palmitic acid derived from FASN is a crucial substrate for protein palmitoylation—a modification essential for the membrane localization and signaling of oncogenic receptors like EGFR—and considering that TRAF2 itself can promote the K63-linked ubiquitination of DYRK1A, facilitating its Golgi translocation and subsequent phosphorylation of Sprouty 2 (which inhibits EGFR degradation and enhances its downstream signaling), inhibiting the TNF-α-TRAF2-FASN axis may simultaneously undermine both palmitate-dependent receptor modification and the TRAF2-DYRK1A-Sprouty 2 signaling module [21, 52, 53]. This dual action would disrupt the stability and activity of the EGFR network at multiple levels, further dismantling pro-tumorigenic signaling pathways. Furthermore, as lipid metabolism is crucial for maintaining cancer stem cell stemness, targeting this pathway may also deplete the GBM stem cell population, which is often responsible for therapy resistance and tumor recurrence [54–56].

This study also has several limitations that should be acknowledged. First, although the concentration of TNF-α (10 ng/mL) used in vitro is commonly adopted in the field, its correspondence to actual pathological levels in the tumor microenvironment of GBM patient tissues remains unclear [17]. Future studies should quantify local TNF-α levels in clinical samples using methods such as mass spectrometry or high-sensitivity immunoassays, thereby providing more direct clinical justification for the selection of in vitro concentrations and further clarifying the concentration threshold of TNF-α-driven biological effects under pathological conditions. Second, while the study revealed that TNF-α enhances TRAF2-mediated K63-linked ubiquitination and stabilization of FASN without altering TRAF2 expression, it remains unknown whether TNF-α further fine-tunes the E3 ligase activity of TRAF2 through post-translational modifications such as phosphorylation. Elucidating this issue would contribute to a more comprehensive understanding of the regulatory network linking inflammatory signaling to abnormal metabolic activation [40, 57]. Additionally, the experiments relied primarily on immunodeficient mouse models, which lack a functional immune system and cannot fully recapitulate the complex tumor-immune microenvironment in humans. Future studies should employ humanized mouse models to more accurately evaluate the function of TNF-α under pathophysiological conditions. Finally, although the small-molecule candidate Jionoside B1 targeting the TRAF2-FASN axis was identified through virtual screening, its selectivity and in vivo pharmacokinetic profile have not been systematically evaluated, warranting further investigation to support its potential for clinical translation. Nevertheless, this study is the first to systematically elucidate the mechanism by which low-dose TNF-α promotes malignant progression in glioblastoma through the TRAF2-FASN axis by regulating lipid metabolism, thereby providing both a candidate compound and an important theoretical foundation for developing this pathway as a therapeutic target.

**Fig. 9:**
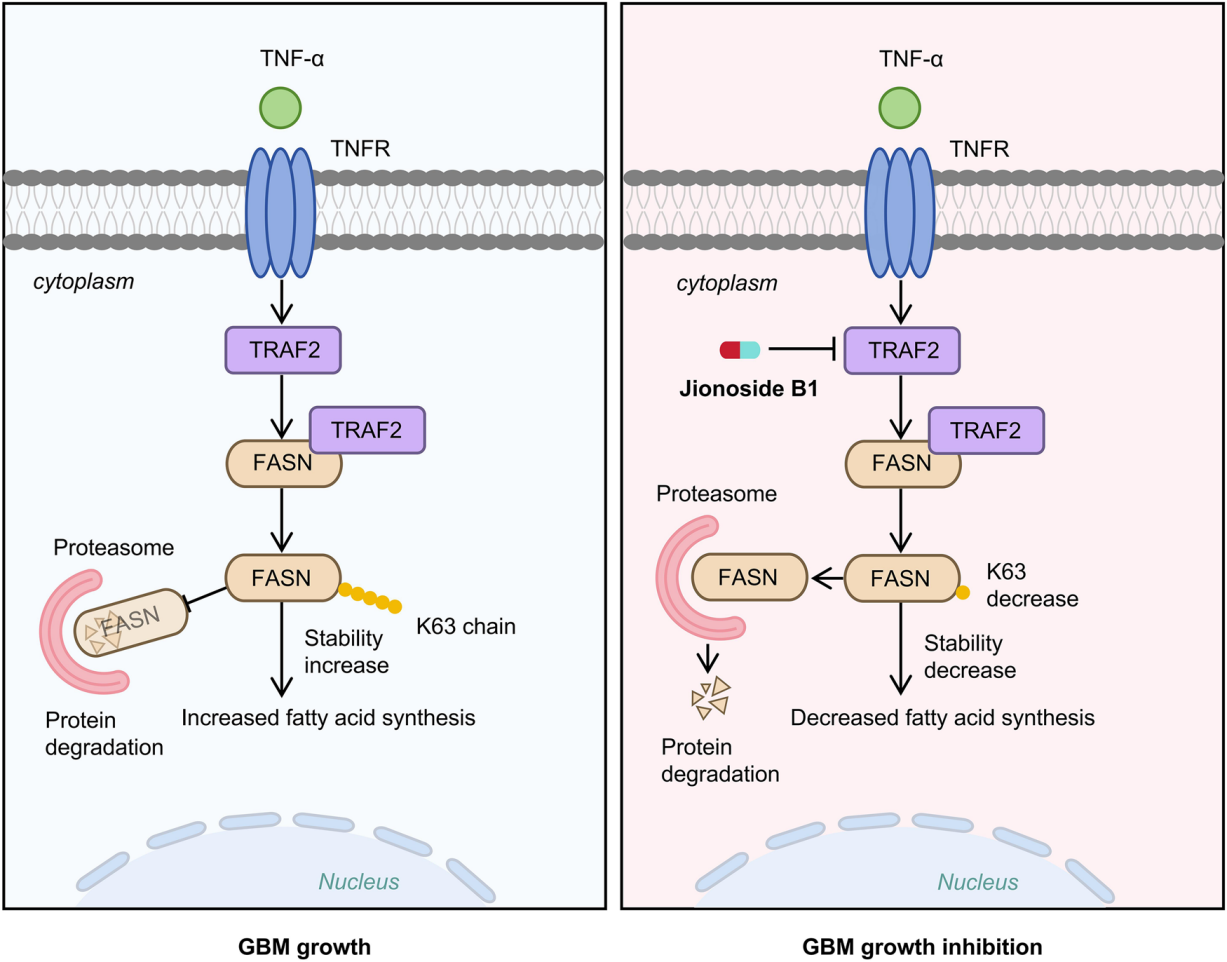
Schematic diagram of the TNF-α-TRAF2-FASN regulatory axis in glioblastoma. A proposed working model of the TNF-α-TRAF2-FASN regulatory axis. Following TNF-α binding to GBM cells, FASN stability is enhanced in a TNF-α-dependent manner, which induces the interaction between TRAF2 and FASN. Subsequently, TRAF2 catalyzes K63-linked polyubiquitination of FASN, leading to increased protein stability and promoting tumor proliferation and growth. Jionoside B1 targets the RING domain of TRAF2, inhibits TRAF2-mediated K63-linked polyubiquitination of FASN, thereby reducing FASN stability and ultimately suppressing glioblastoma proliferation and growth.

In summary, our findings unveil a previously unrecognized mechanism in GBM (Fig. 9), whereby microenvironmental TNF-α activates TRAF2 via TNFR. TRAF2 subsequently binds to FASN and stabilizes the FASN protein through K63-linked ubiquitination, thereby promoting lipogenesis and tumor progression. Jionoside B1 disrupts this axis by inhibiting the E3 ligase activity of TRAF2, leading to FASN destabilization and tumor suppression. We redefine TRAF2 not only as a signaling adaptor but also as a key regulator that directly modulates lipid synthesis by stabilizing FASN. The newly identified TRAF2-FASN link elucidates a crucial connection between inflammatory signaling and cancer lipogenesis. Given the dismal prognosis of GBM and the limitations of current therapies, developing novel agents (such as Jionoside B1) capable of disrupting the TRAF2-FASN interaction or inhibiting TRAF2’s E3 ligase activity holds promise for providing new targeted therapeutic strategies against this lethal disease.

## Methods and Materials

### Immnuohistochemical (IHC) staining

A total of 53 clinical glioma tissue specimens were included in this study, all collected from patients who underwent surgical resection in the Department of Neurosurgery at Wuxi People’s Hospital Affiliated with Nanjing Medical University. Additionally, normal brain tissues (NBT) from four patients with traumatic brain injury were collected as the control group. Written informed consent was obtained from all patients before sample collection, and the study protocol was approved by the Ethics Committee of Wuxi People’s Hospital Affiliated with Nanjing Medical University (Approval No.: KY21058). The clinical characteristics and inclusion/exclusion criteria of this cohort are detailed in Supplementary Table 1.

Briefly, tissue sections were incubated with primary antibodies (Supplementary Table 2) according to the manufacturer’s instructions (Abcam, Cambridge, UK) and detected using the Mouse and Rabbit Specific HRP/DAB (ABC) Detection IHC Kit. Nuclei were lightly counterstained with crystal violet. Specificity of IHC staining was confirmed using normal mouse or rabbit IgG. Expression levels were evaluated by the following formula: Expression level = percentage of positive cells score × staining intensity score. The percentage score was defined as: 0 (0%), 1 (1–25%), 2 (26–50%), 3 (51–75%), and 4 (76–100%). The intensity score was defined as: 0 (none), 1 (weak), 2 (moderate), and 3 (strong). All tissue sections were examined and scored simultaneously by two blinded pathologists. The average immunohistochemical reactivity score was considered the final score.

### Cell Culture and Reagents

The human glioma cell lines (A172, U87, U251) and the human embryonic kidney cell line 293 T were obtained from the China Center for Type Culture Collection (CCTCC, Shanghai, China). The Sw1783 cell line and Normal Human Astrocytes (NHA) were purchased from Lonza (Clonetics, Walkersville, MD, USA). All cells were routinely cultured in Dulbecco’s Modified Eagle Medium (DMEM) supplemented with 10% fetal bovine serum (FBS; CellBio, Australia) at 37 °C in a 5% CO_2_ atmosphere using a HCP-80E incubator. All cell lines were authenticated by short tandem repeat (STR) profiling and confirmed to be free of mycoplasma contamination. Stable transfectants were constructed via lentiviral vector transduction, and puromycin (MCE) selection was applied 24–48 h post-transfection.

### Online cancer database analysis

The TCGA-GBM database from GEPIA2 was used for comparative analysis of TNFRSF1A and TNFRSF1B mRNA expression levels between GBM and healthy brain tissues. Clinical analysis data for TNFRSF1A, TNFRSF1B, and TRAF2 were obtained from The Cancer Genome Atlas (TCGA), the Chinese Glioma Genome Atlas (CGGA), the Repository of Molecular Brain Neoplasia Data (REMBRANDT), and the Gravendeel database. Gene expression profiles and corresponding clinical information from TCGA, CGGA, REMBRANDT, and Gravendeel were all acquired and processed through the GlioVis data portal (http://gliovis.bioinfo.cnio.es).

To investigate the biological functions of TNF(Tumor Necrosis Factor) in glioblastoma, the following analyses were conducted in this study: First, RNA sequencing data from TCGA_GBM were obtained via the GlioVis data portal (https://gliovis.shinyapps.io/GlioVis/). Subsequently, samples were divided into high-expression and low-expression groups based on the median TNF mRNA expression level, and differential expression gene analysis was performed between the two groups. Finally, the identified differentially expressed genes were submitted for Gene Set Enrichment Analysis (GSEA), using predefined gene sets from the Molecular Signatures Database (https://www.gsea-msigdb.org/gsea).

### Vector Construction and Transduction

Full-length cDNA sequences of human TRAF2 and FASN were obtained by PCR amplification and verified by sequencing. The His-tagged lentiviral vector GV712 (provided by Shanghai GeneChem) was used to construct the HIS-TRAF2 lentivirus. The FASN cDNA was inserted into the FLAG-tagged vector CV702 to generate the FLAG-FASN expression plasmid. Using wild-type TRAF2 as a template, truncated mutants of its RING, Zinc Fingers, and TRAF domains were constructed by the same company. Plasmids for hemagglutinin (HA)-tagged wild-type ubiquitin (WT) and its lysine mutants (K48, K63) were also purchased from Shanghai GeneChem. The shRNA target sequences for knocking down TRAF2 and FASN are listed in Supplementary Table 3.

Transient transfection experiments were performed using Lipofectamine 3000 (Invitrogen) according to the manufacturer’s instructions. Stable transfection cell lines were obtained by selection with puromycin (MCE).

### RNA Extraction and Reverse Transcription Quantitative PCR

Total RNA was extracted from glioma cells using TRIzol reagent (Invitrogen) according to the manufacturer’s instructions. Subsequently, the RNA was reverse transcribed into cDNA using the PrimeScript RT reagent kit (Vazyme). Using the resulting cDNA as a template, quantitative real-time PCR (qPCR) was performed with Real SYBR Mix (Vazyme, China) on a LightCycler 480 II system (Roche Applied Science). All primers were synthesized by Sangon Biotech (Shanghai) Co., Ltd., and the sequence information is provided in Supplementary Table 4. The relative expression levels of the target genes were calculated using the 2^*–*ΔΔCT^ method with β-actin as the endogenous control.

### Western Blot Analysis

Cells were lysed using RIPA lysis buffer (Cell Signaling Technology) containing protease inhibitors (Cwbio) to extract total protein. The protein lysates were mixed with SDS-PAGE Loading Buffer (Cwbio) and boiled for denaturation. Equal amounts of protein were loaded for SDS-PAGE gel electrophoresis. Following electrophoresis, proteins were transferred onto Immobilon™ PVDF membranes (Millipore) at a constant current of 400 mA. The membranes were blocked with rapid blocking buffer (Beyotime) and then incubated with primary antibodies at 4 °C overnight. After washing with TBST the next day, the membranes were incubated with corresponding secondary antibodies at room temperature. Following another wash, immunoreactive bands were visualized using a chemiluminescent substrate (Millipore) and imaged with a Tanon system. Detailed antibody information is provided in Supplementary Table 2, and uncropped original blot images are shown in Original western blots.

### Reagents

The recombinant human TNF-α protein (#16769) was purchased from Cell Signaling Technology. Cycloheximide (HY-12320), MG-132 (HY-13259), Chloroquine (HY-17589A), R-7050 (HY-110203), Shancigusin I (HY-N8183), Rebaudioside F (HY-N6887), Jionoside B1 (HY-N2218), and Carnostatine hydrochloride (HY-112431A) were obtained from MedChemExpress.

### Cell growth assay and colony formation assay

Cell growth was detected using the Cell Counting Kit-8 (Biosharp, BS350C) according to the manufacturer’s instructions. Absorbance was measured with a microplate reader (Thermo Scientific) for quantitative analysis of cell growth. Cell proliferation ability was evaluated using the EdU incorporation assay following the protocol of the EdU detection kit (Beyotime, C0075L). Images were captured using an Invitrogen EVOS FL Auto microscope (Life Technologies) and processed with ImageJ software for image composition and cell counting. The proliferation level was determined by calculating the ratio of EdU-positive cells to Hoechst-positive cells.

For the colony formation assay, 1000 corresponding cells were seeded into 6-well plates (Corning, 3516) and cultured in medium containing 10% fetal bovine serum (FBS) for two weeks. The colonies were then fixed with 4% paraformaldehyde (Biosharp, BL538A) for 15 min, stained with 0.1% crystal violet (Beyotime, C0121) for 30 min, and counted for analysis.

### Invasion, Migration, and Wound-Healing Assay

Cell migration and invasion assays were performed using 24-well Transwell chambers (pore size: 8 μm; Corning Costar; 3422). For the invasion assay, the upper chambers were pre-coated with 100 μl of Matrigel matrix (Corning) diluted at a 1:9 ratio, while the migration assay required no coating. Tumor cells (2 × 10⁴) were resuspended in 200 μl of serum-free medium and seeded into the upper chamber, while the lower chamber was filled with 600 μl of DMEM containing 10% FBS. After 36 h of incubation at 37 °C, the cells were fixed with 4% paraformaldehyde (Biosharp, BL538A) and stained with 0.1% crystal violet (Beyotime, C0121). Images were captured using an Invitrogen EVOS FL Auto microscope (Life Technologies). Following dissolution of the stained cells with 3% glacial acetic acid, the numbers of migrated and invaded cells were quantitatively analyzed by measuring the absorbance of the solution at 450 nm.

For the wound healing assay, Ibidi Culture-Inserts (500 μm gap, Ibidi, Germany) were used. Cells were seeded in 6-well plates pre-equipped with the inserts. Once the cells reached approximately 90% confluence, the inserts were removed to create a uniform wound area. After washing with PBS, the medium was replaced with serum-free DMEM for further culture. Wound images were captured at 0 h and 24 h, and the wound closure was quantitatively analyzed using ImageJ software.

### Double Immunofluorescence Staining

Cells were seeded in 24-well plates containing glass coverslips and cultured. Plasmid transfection was performed using Lipofectamine 3000 reagent according to the manufacturer’s protocol. Twenty-four hours post-transfection, cells were fixed with 4% paraformaldehyde (Biosharp, BL538A) for 15 min. After three washes with PBS, cells were permeabilized with Immunostaining Strong Permeabilization Buffer (Beyotime, P0097) for 15 min at room temperature, followed by another three PBS washes. Subsequently, cells were blocked with Immunostaining Blocking Buffer (Beyotime, P0102) for 1 h at room temperature and then incubated with primary antibodies overnight at 4 °C. Following PBS washes, cells were incubated in the dark with Alexa Fluor® 555 (A32727, Invitrogen) and Alexa Fluor® 488 (A32731, Invitrogen)-conjugated secondary antibodies for 1 h at room temperature. After final PBS washes, coverslips were mounted onto glass slides using anti-fade mounting medium containing Hoechst 33342 (Beyotime, P0133) and sealed with nail polish. Images were finally acquired using an Invitrogen EVOS FL Auto microscope (Life Technologies).

### Immunoprecipitation assays

Total protein was extracted from GBM and 293 T cells following the method described above. After high-speed centrifugation, the supernatant was collected and incubated with appropriate antibodies overnight at 4 °C, followed by further incubation with Protein A/G magnetic beads (MedChemExpress, HY-K0202) for 12 h at 4 °C. The immunocomplexes were thoroughly washed five times with Western and IP cell lysis buffer (Beyotime, P0013), and the bound proteins were eluted by boiling in SDS-PAGE loading buffer. Finally, the proteins were separated by SDS-PAGE and analyzed by western blotting. Detailed information on the antibodies used can be found in Supplementary Table 2.

### Mass Spectrometry (MS Analysis of TRAF2 Interacting Proteins)

U251 cells transfected with His-TRAF2 were lysed and the supernatant was collected. The supernatant was incubated with His antibody overnight at 4 °C, followed by further incubation with Protein A/G magnetic beads (MedChemExpress, HY-K0202) for 12 h at 4 °C. The immunocomplexes were thoroughly washed five times with western blotting and IP lysis buffer (Beyotime, P0013), and the bound proteins were eluted by boiling. Subsequently, the samples were subjected to MS analysis by Novogene (Beijing, China). Detailed information of the TRAF2 mass spectrometry is provided in Supplementary Table 5.

### RNA-Sequencing (RNA-seq) Analyses

Total RNA from the U251-TNFα treatment group and control group (*n* = 3) was extracted using the TRIzol method (Invitrogen). The construction of transcriptome sequencing libraries, sequencing, and bioinformatic analysis for these samples were conducted by Shanghai Genechem Co., Ltd (Shanghai, China).

Meanwhile, total RNA from the U251-TRAF2 knockdown group and its corresponding control group (*n* = 3) was also extracted using TRIzol reagent (Invitrogen). The RNA-seq library preparation, sequencing, and subsequent bioinformatic analysis for this set of samples were performed by OE Biotech Co., Ltd (Shanghai, China).

### Fatty Acid Metabolism Detection

The formation of lipid droplets in cells was assessed using Nile Red staining, performed according to the instructions of the Lipid Droplets Red Fluorescence Assay Kit with Nile Red (Beyotime, C2051S). Fluorescence images were captured using an Invitrogen EVOS FL Auto microscope (Life Technologies).

Intracellular triglyceride levels were measured using a Triglyceride Assay Kit (NanJing JianCheng Bioengineering Institute, A110-1-1), with all steps strictly following the manufacturer’s protocol. Finally, optical signals at specified wavelengths were detected using a microplate reader (Thermo Scientific).

### Tumour xenografts in nude mice

Four-week-old male nude mice were purchased from the Shanghai Institute of Life Sciences, Chinese Academy of Sciences, and housed under specific pathogen-free (SPF) conditions. After a one-week acclimatization period, the mice were randomly assigned to different experimental groups. In the intracranial tumor model, U251 cells stably expressing luciferase (5 × 10⁵ cells per mouse) were stereotaxically implanted into the right striatum of each mouse (*n* = 4 per group). Starting from day 14 post-surgery, intraperitoneal injections of either DMSO or Jionoside B1 (20 mg/kg/day) were administered every two days. Tumor growth was monitored using the IVIS Spectrum in vivo imaging system on days 14 and 28 post-surgery, and the fluorescence signals were quantified with Living Image software. In the subcutaneous tumor model, 5 × 10⁶ U251 cells (transfected with TRAF2-NC, TRAF2-OE, sh-NC, or sh-TRAF2, respectively) were subcutaneously inoculated into the flank of each nude mouse (*n* = 5 per group). 28 days after inoculation, the mice were euthanized, and tumor tissues were harvested for measurement of volume and weight. All animal experimental procedures were strictly performed in accordance with the National Institutes of Health Guide for the Care and Use of Laboratory Animals. The animal experiments were conducted by two technicians who were blinded to the treatment conditions of the mice. The experimental protocol was approved by the Animal Ethics Committee of Nanjing Medical University (Approval No: DL2025057).

### Small Molecule Virtual Screening

The three-dimensional structure of Human TRAF2 was retrieved from the PDB database (PDB ID: 3KNV). Hydrogen atoms were added, metal ions and water molecules were removed using the Protein Preparation Wizard module, and the structure was optimized with the OPLS2005 force field to an RMSD of 0.30 Å. A grid file was generated with a 20 Å × 20 Å × 20 Å box centered on the key amino acid H51. The HY-L001p MCE Bioactive Compound Library Plus (containing 25,000 compounds) was processed using the LigPrep module to add hydrogens and optimize energy, resulting in 3D structures. A three-step progressive docking approach was then performed using the Glide module, involving High-Throughput Virtual Screening (HTVS), Standard Precision (SP), and Extra Precision (XP) modes. In each step, the top 30% of scored compounds were retained. Based on target binding affinity and structural characteristics, the top 200 compounds from the HY-L001p MCE Bioactive Compound Library Plus were selected as the final output. The structures, docking scores, and supplementary information of these top 200 compounds are provided in Supplementary Table 6.

### Statistical Analysis

All experiments were independently repeated at least three times, and data are presented as mean ± standard deviation. Statistical analysis was performed using SPSS 20.0 software (IBM Corp., Boston, MA, USA), with all data meeting the assumptions of normal distribution and homogeneity of variance. Comparisons between two groups were conducted using the independent samples t-test, while comparisons among multiple groups were performed using one-way analysis of variance (ANOVA), followed by Tukey’s post hoc test for multiple comparisons. Survival analysis utilized the Kaplan-Meier method, and comparisons were conducted via the log-rank test. Overall survival (OS) is the duration from diagnosis to death from any cause. Spearman’s rank correlation was used to evaluate group correlations. A *P*-value < 0.05 was considered statistically significant. GraphPad Prism 9.5 software (GraphPad Software, Boston, MA, USA) was used to generate graphs and perform statistical analyses.

## Supplementary information

Supplementary Figure1-7

Supplemental Data 1-4

Supplementary Table 1

Supplementary Table 2

Supplementary Table 3

Supplementary Table 4

Supplementary Table5

Supplementary Table6

Original Western blots

## Data Availability

RNA-seq data generated for this study are available in the Gene Expression Omnibus database (No. GSE318064, GSE318134). All data accessed from external sources and prior publications have been referenced in the text and corresponding figure legends. Additional data are available upon request. The data that support the findings of this study are available from the corresponding author upon reasonable request.
