# Supplementary material for: Low-dose TNF-α drives malignant progression and lipid metabolism in glioblastoma through the TRAF2-FASN axis
Source: Cell Death Discov. 2026 Apr 9;12:242. doi: 10.1038/s41420-026-03087-x (PMC13187350; doi:10.1038/s41420-026-03087-x)
Supplement: Supplementary file 1 — Supplementary Figure1-7 [file 41420_2026_3087_MOESM1_ESM.docx]

**Supplementary Figures**


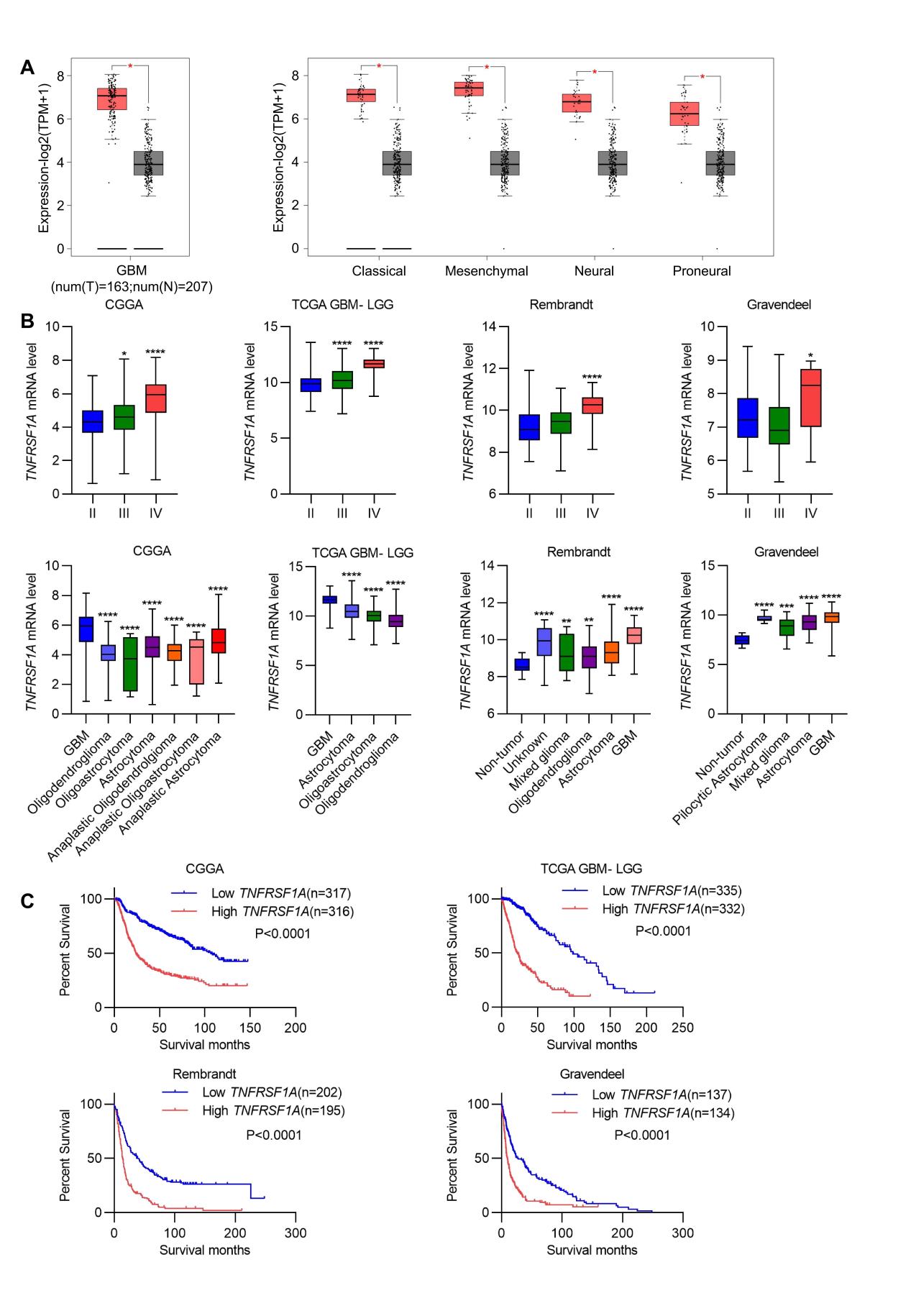


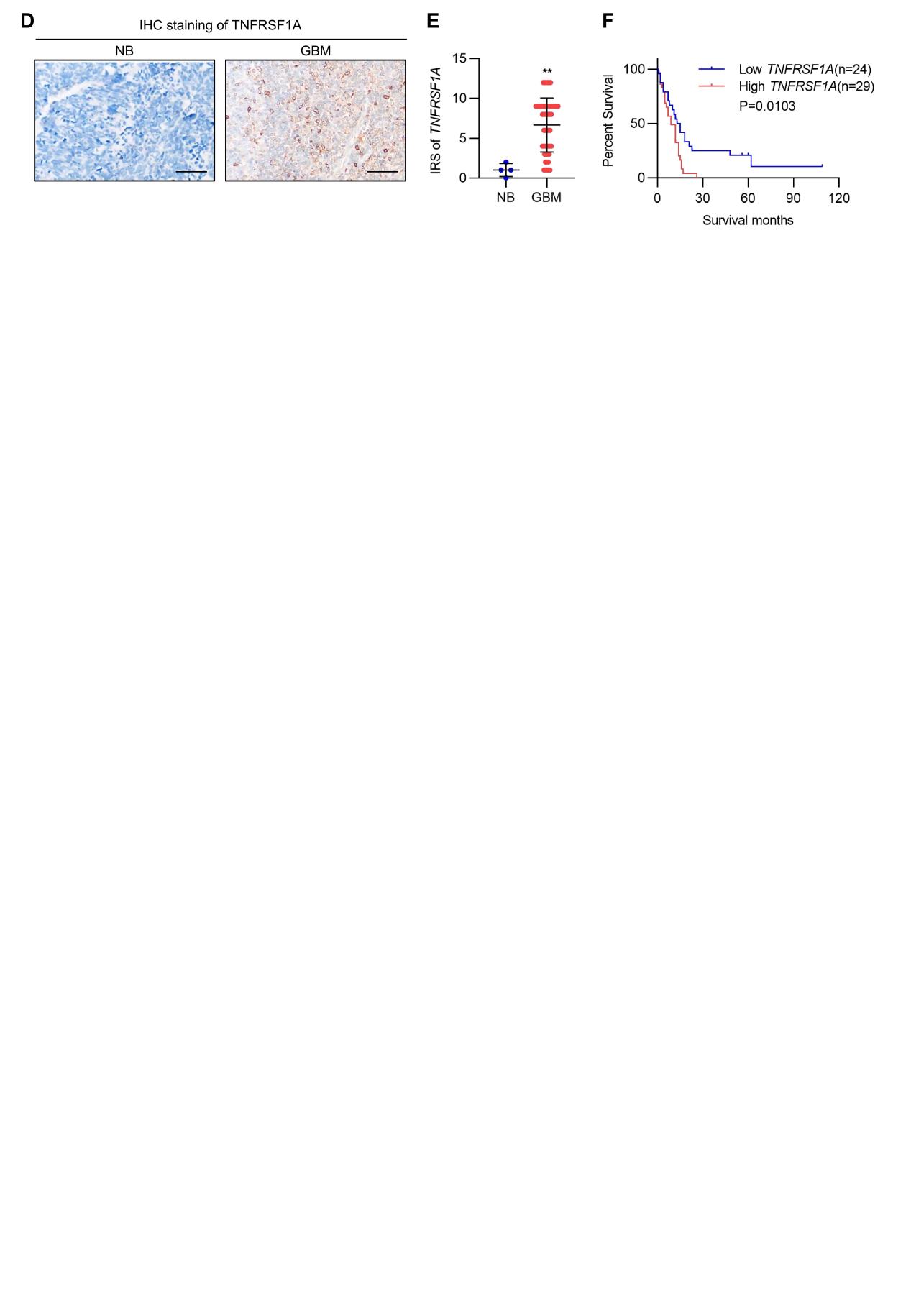


**Figure S1. TNFRSF1A is highly expressed in GBM and its expression correlates with tumorigenesis.**

**A.** Analysis of the TCGA database using GEPIA2 revealed significant differences in TNFRSF1A mRNA expression levels between glioblastoma samples (pink) and normal tissues (gray), with variations also observed across different GBM subtypes.

**B.** Multiple designated glioma datasets showed that TNFRSF1A is highly expressed across various glioma grades and GBM subtypes (two-tailed Student’s t-test, *p<0.05, **p<0.01, ***p<0.001, ****p<0.0001).

**C.** Overall survival analysis based on TNFRSF1A mRNA expression levels in a specified GBM dataset (Kaplan-Meier survival test).

**D.** Representative immunohistochemical staining images of TNFRSF1A in patient-derived normal brain (NB) and glioblastoma (GBM) tissues. Scale bar, 100 µm.

**E.** Compared with normal brain tissues, TNFRSF1A expression was significantly elevated in GBM tissues (**p<0.01, unpaired t-test).

**F.** Overall survival analysis based on TNFRSF1A expression levels in GBM patients (Kaplan-Meier survival analysis, Log-rank χ² = 6.578, p = 0.0103). Patients were grouped according to the median immunohistochemical reactivity score of TNFRSF1A in GBM tissues.


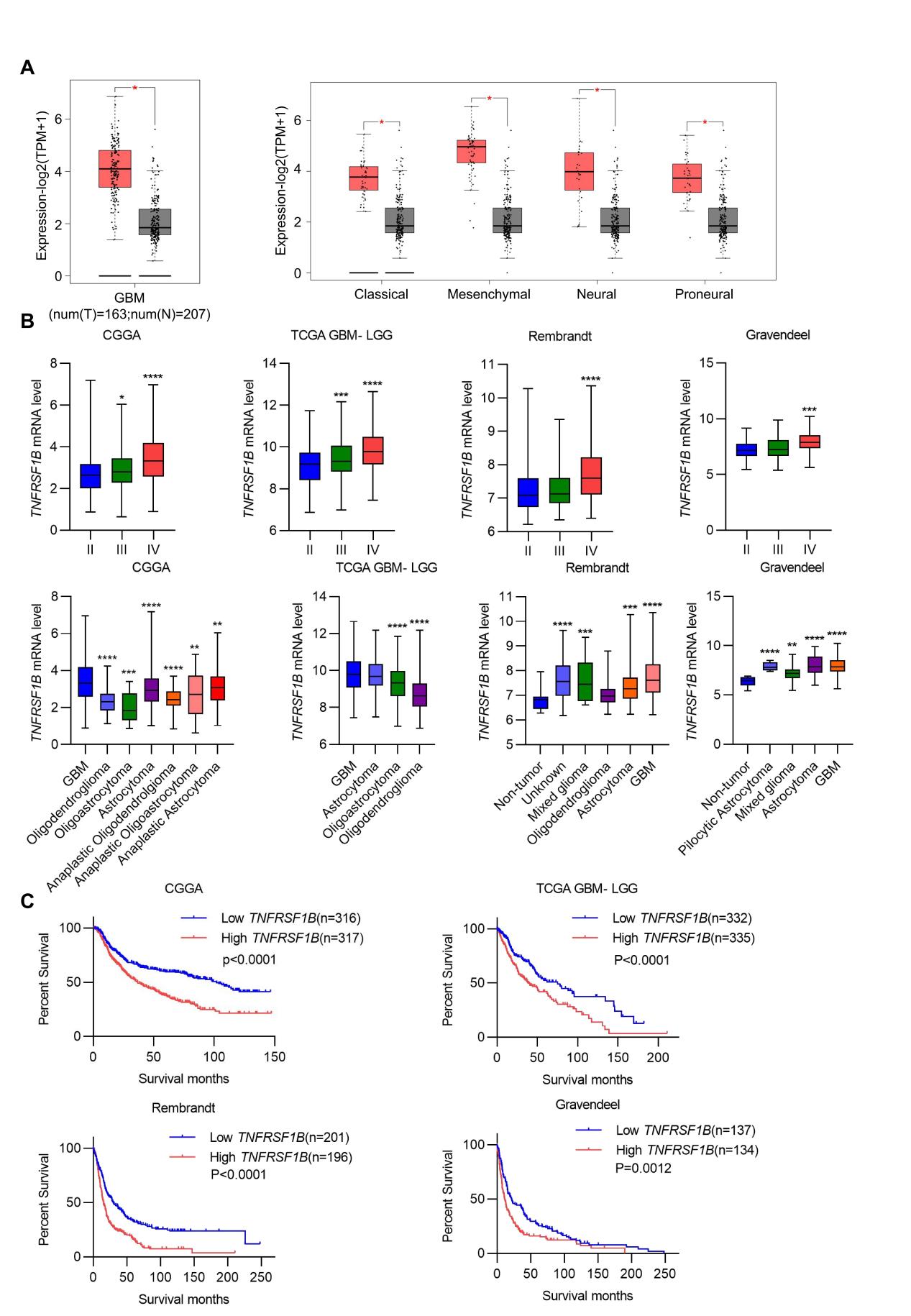





**Figure S2. TNFRSF1B is highly expressed in GBM and its expression correlates with tumorigenesis.**

**A.** Analysis of the TCGA database using GEPIA2 revealed significant differences in TNFRSF1B mRNA expression levels between glioblastoma samples (pink) and normal tissues (gray), with variations also observed across different GBM subtypes.

**B.** Multiple designated glioma datasets showed that TNFRSF1B is highly expressed across various glioma grades and GBM subtypes (two-tailed Student’s t-test, *p<0.05, **p<0.01, ***p<0.001, ****p<0.0001).

**C.** Overall survival analysis based on TNFRSF1B mRNA expression levels in a specified GBM dataset (Kaplan-Meier survival test).

**D.** Representative immunohistochemical staining images of TNFRSF1B in patient-derived normal brain (NB) and glioblastoma (GBM) tissues. Scale bar, 100 µm.

**E.** Compared with normal brain tissues, TNFRSF1B expression was significantly elevated in GBM tissues (**p<0.01, unpaired t-test).

**F.** Overall survival analysis based on TNFRSF1B expression levels in GBM patients (Kaplan-Meier survival analysis, Log-rank χ² = 7.936, p = 0.0048). Patients were grouped according to the median immunohistochemical reactivity score of TNFRSF1B in GBM tissues.


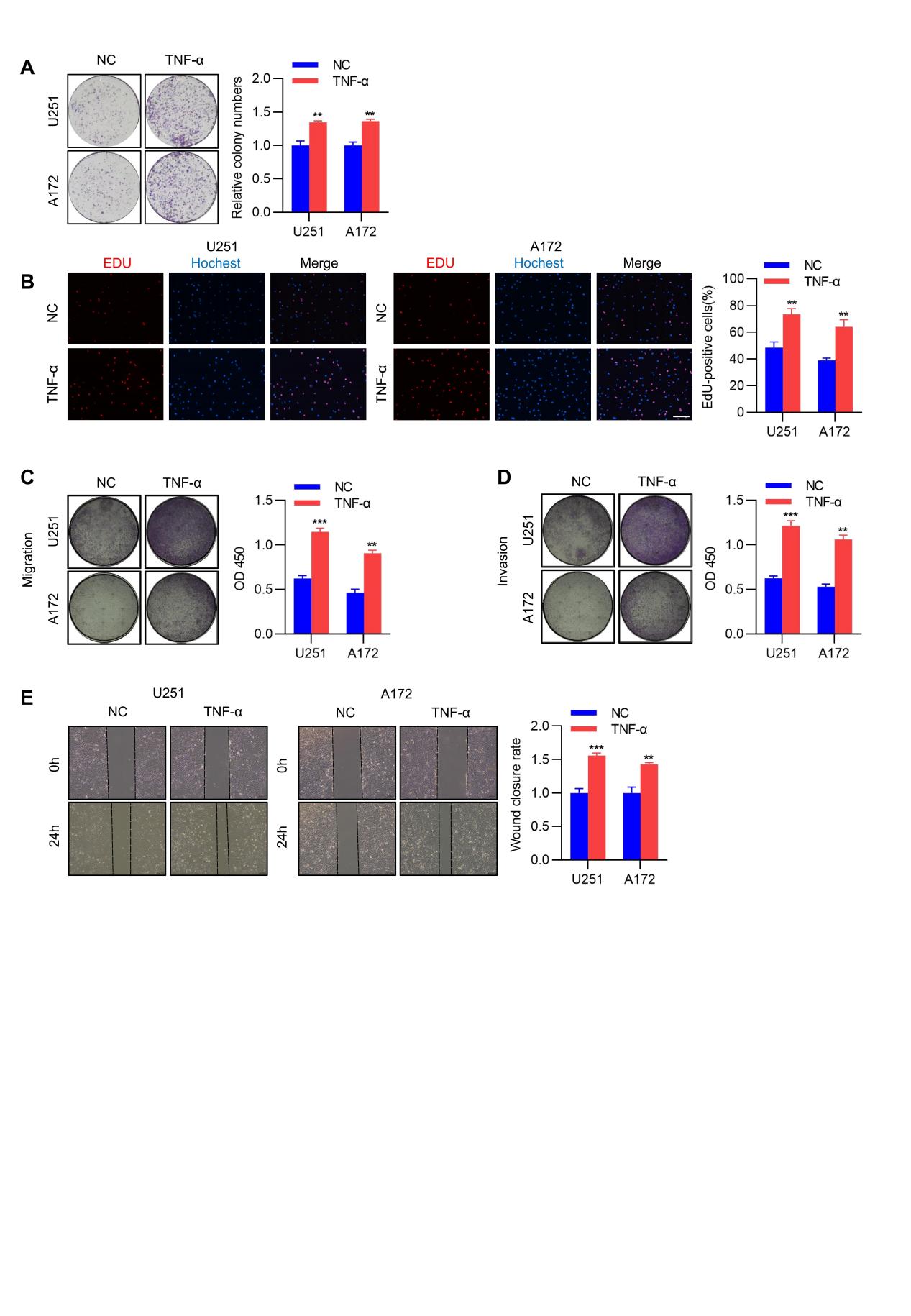


**Figure S3. Low-dose TNF-α promotes malignant progression of GBM.**

**A.** Colony formation assay was performed on U251 and A172 cells treated with TNF-α (10 ng/mL). Quantitative results are presented as a histogram (n=5, Student's t-test, **p<0.01).

**B.** Fluorescence images from the EdU assay of U251 and A172 cells after 24-hour treatment with TNF-α (10 ng/mL). Data are expressed as the percentage of EdU-positive cells (red) among total Hoechst-stained cells (blue). Scale bar, 200 µm. Quantitative results are presented as a histogram (n=5, Student's t-test, **p<0.01).

**C,D.** The effects of TNF-α (10 ng/mL) on the migration and invasion capabilities of U251 and A172 cells were assessed using Transwell assays. Quantitative results are presented as histograms (n=5, Student's t-test, **p<0.01, ***p<0.001).

**E.** The effect of TNF-α (10 ng/mL) on the motility of U251 and A172 cells was evaluated using the ibidi wound healing assay. Quantitative results are presented as a histogram (n=5, Student's t-test, **p<0.01, ***p<0.001).


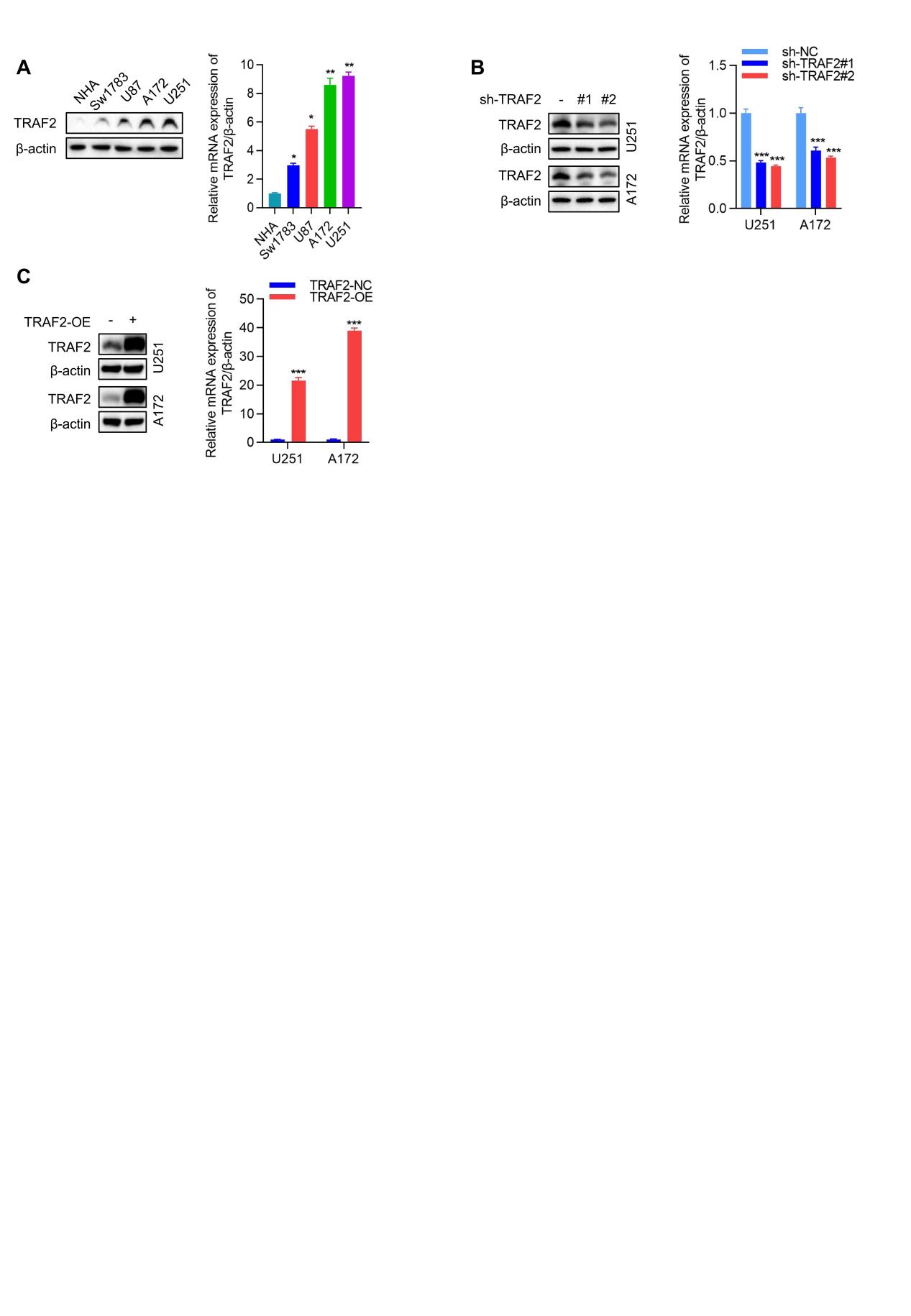


**Figure S4. TRAF2 is highly expressed in glioblastoma cells, as validated by knockdown and overexpression experiments..**
**A.** Western blot and RT-qPCR analyses showed that compared to the low-grade glioma (LGG) cell line Sw1783, TRAF2 expression was lower in normal human astrocytes (NHA), while it was significantly upregulated in GBM cell lines (U87, A172, U251), with the highest levels observed in A172 and U251 cells (n=5, Student's t-test, *p<0.05, **p<0.01).

**B,C.** Western blot and RT-qPCR were used to detect TRAF2 protein and mRNA expression levels in U251 and A172 cells after TRAF2 knockdown or overexpression, respectively(n=5, Student's t-test, ***p<0.001).


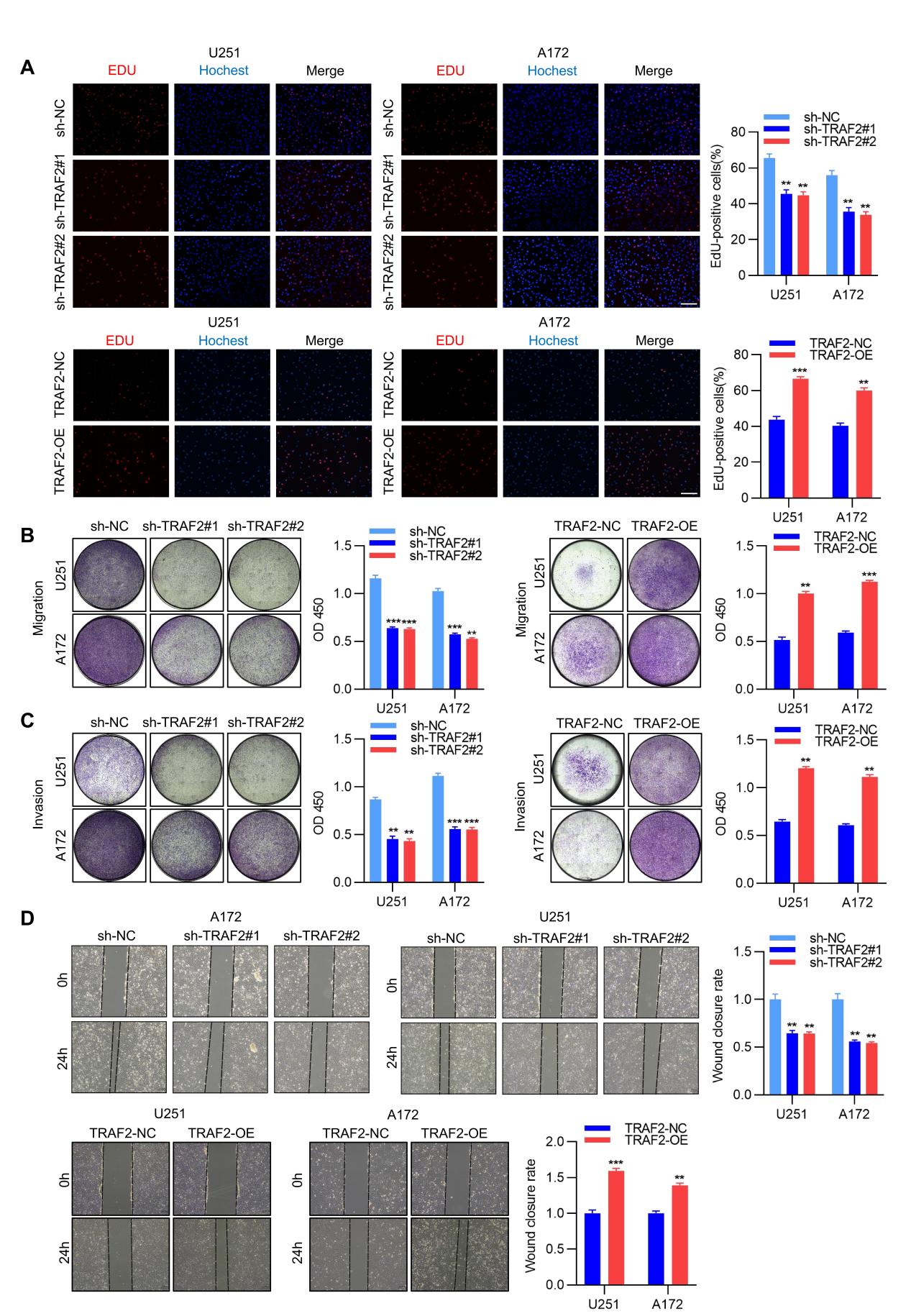


**Figure S5. TRAF2 is essential for the proliferation, migration and invasion of GBM cells.**

**A.** The effects of TRAF2 knockdown or overexpression on DNA replication and proliferation capacity in U251 and A172 cells were determined by EdU assay, respectively. Scale bar, 200 µm. Quantitative results are presented as histograms (n=5, Student's t-test, **p < 0.01, ***p < 0.001).

**B-C.** The effects of TRAF2 knockdown or overexpression on the migration and invasion abilities of U251 and A172 cells were evaluated using Transwell assays, respectively. Quantitative results are presented as histograms (n=5, Student's t-test, **p < 0.01, ***p < 0.001).

**D.** The effects of TRAF2 knockdown or overexpression on the wound closure capacity of U251 and A172 cells were assessed using ibidi wound healing assays, respectively. Quantitative results are presented as histograms (n=5, Student's t*-*test, **p < 0.01, ***p < 0.001).


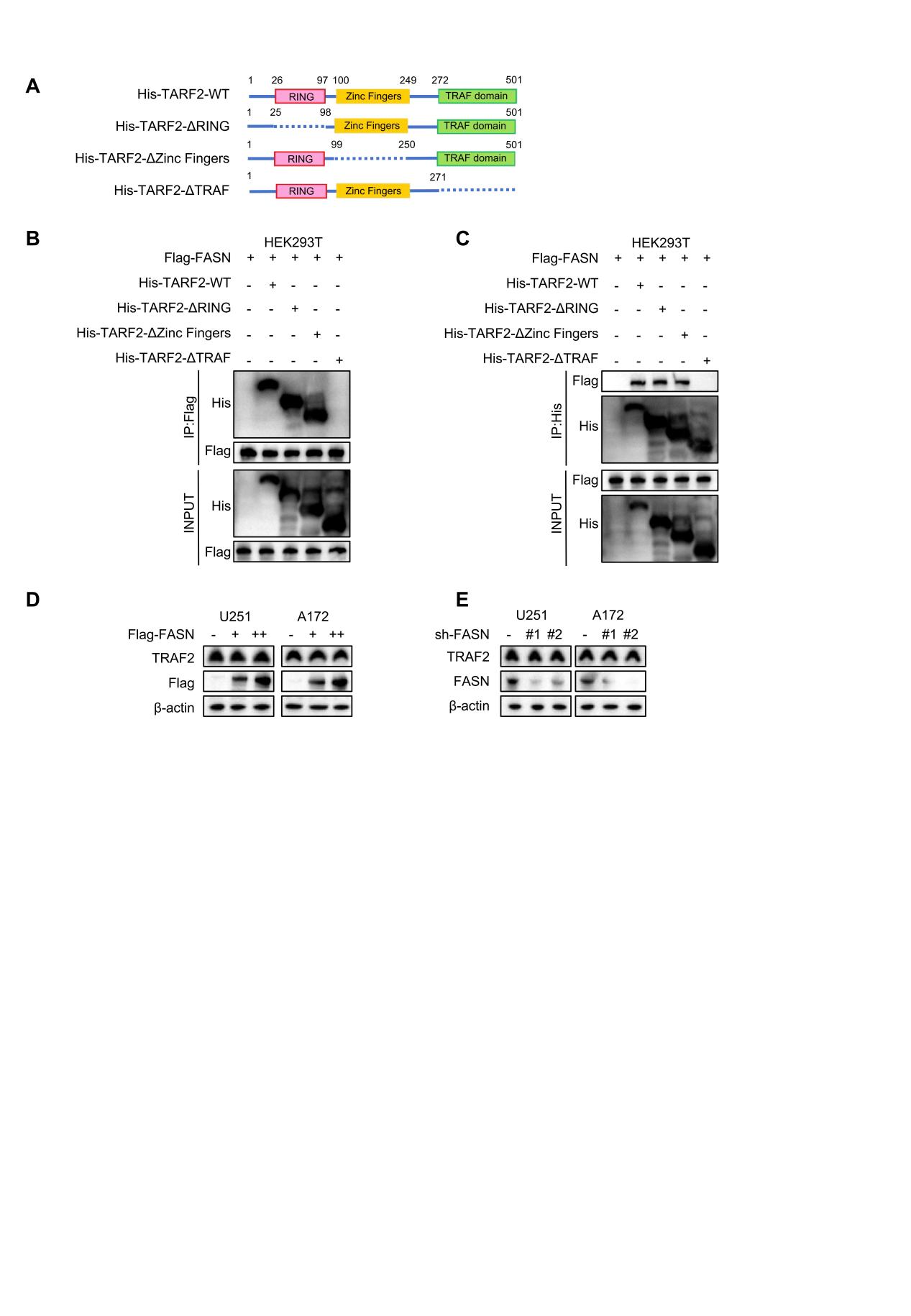


**Figure S6. TRAF2 interacts with FASN via its TRAF domain, and FASN does not regulate TRAF2 expression.**

**A.** Schematic representation of TRAF2 and its truncated variants. A series of His-tagged truncated proteins were constructed based on the functional domains of TRAF2 (RING, Zinc Fingers, and TRAF domain).

**B,C.** The TRAF domain mediates the interaction with FASN.

**D.** Western blot analysis was performed to detect TRAF2 protein expression in FASN-overexpressing U251 and A172 cells.

**E.** Western blot analysis was performed to detect TRAF2 protein expression in FASN-knockdown U251 and A172 cells.


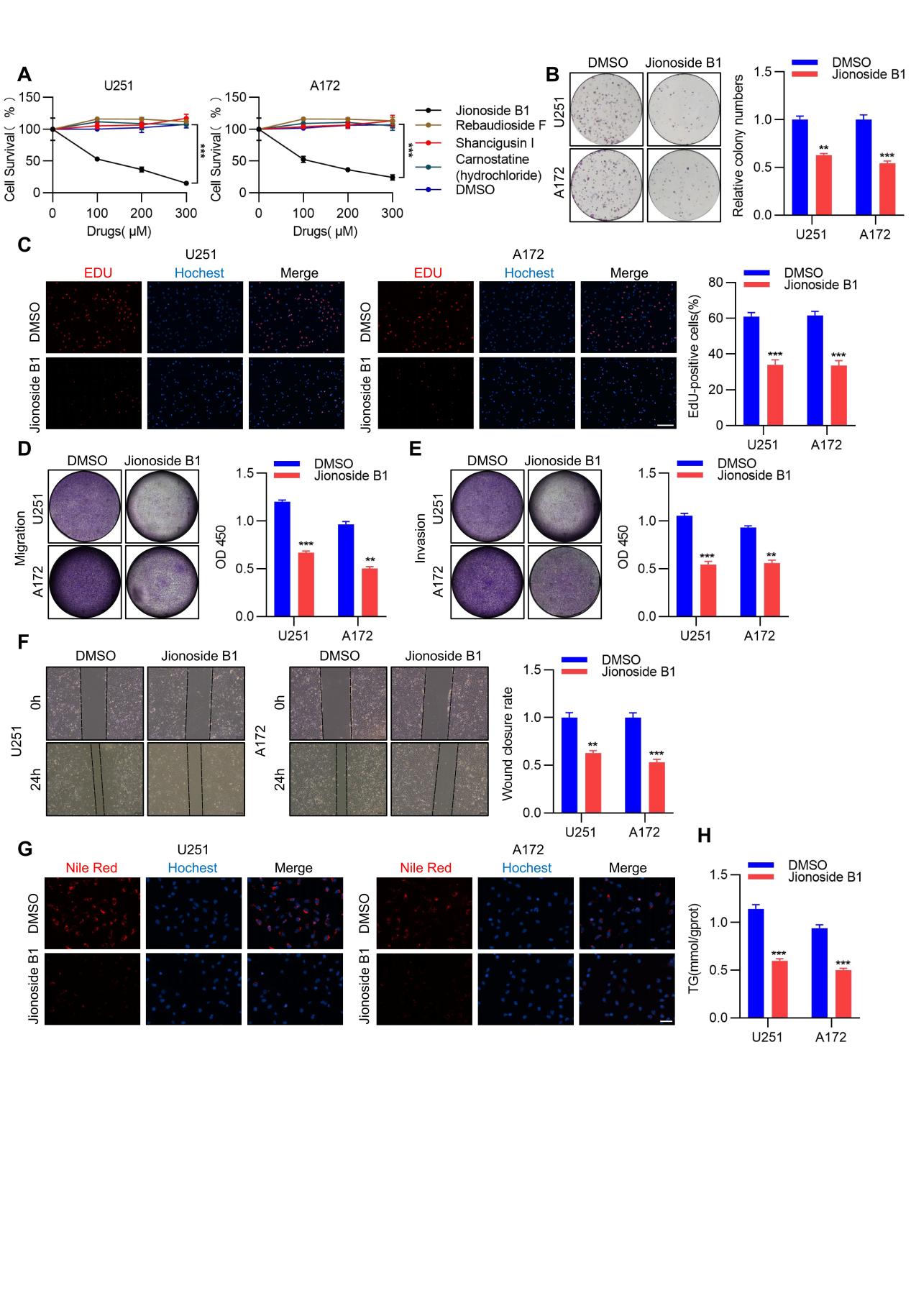


**Figure S7. Targeting TRAF2 suppresses malignant phenotypes and lipid metabolism in GBM.**

**A.** To evaluate the cytotoxicity of candidate compounds on U251 and A172 cells, both cell lines were treated with gradient concentrations of each candidate compound for 24 h, followed by cell viability measurement using the CCK-8 assay (n=5, Student's t-test, ***p < 0.001).

**B.** The effect of Jionoside B1 (100 μM) on the colony formation ability of U251 and A172 cells was determined by colony formation assay. Quantitative results are presented as histograms (n=5, Student's t-test, **p < 0.01, ***p < 0.001).

**C.** The effects of Jionoside B1 (100 μM) on DNA replication and proliferation capacity in U251 and A172 cells were determined by EdU assay. Data are expressed as the percentage of EdU-positive cells (red) relative to the total number of Hoechst-stained cells (blue). Scale bar, 200 µm. Quantitative results are presented as histograms (n=5, Student's t-test, ***p < 0.001).

**D,E.** The effects of Jionoside B1 (100 μM) on the migration and invasion abilities of U251 and A172 cells were evaluated using Transwell assays. Quantitative results are presented as histograms (n=5, Student's t-test, **p < 0.01, ***p < 0.001).

**F.** The effect of Jionoside B1 (100 μM) on the wound closure capacity of U251 and A172 cells was assessed using the ibidi wound healing assay. Quantitative results are presented as histograms (n=5, Student's t-test, **p < 0.01, ***p < 0.001).

**G.** Lipid droplet formation ability in U251 and A172 cells treated with Jionoside B1 (100 μM) for 24 h was evaluated by Nile Red staining (n=5). Scale bar, 50 μm.

**H.** Triglyceride (TG) synthesis capacity in U251 and A172 cells treated with Jionoside B1 (100 μM) for 24 h was assessed using a biochemical kit. Quantitative results are presented as a histogram (n=5, Student's t-test, ***p < 0.001).
