# Supplementary material for: Low-dose TNF-α drives malignant progression and lipid metabolism in glioblastoma through the TRAF2-FASN axis
Source: Cell Death Discov. 2026 Apr 9;12:242. doi: 10.1038/s41420-026-03087-x (PMC13187350; doi:10.1038/s41420-026-03087-x)
Supplement: Supplementary file 2 — Supplemental Data 1-4 [file 41420_2026_3087_MOESM2_ESM.docx]

**Supplemental Data 1. Association between TNFRSF1A expression and information of patients with GBM.**

| Status | | TNFRSF1A expression^#^, n | | Total | *p* value* |
| --- | --- | --- | --- | --- | --- |
|  |  | LOW | HIGH | n |  |
| Gender | Male | 12 | 14 | 26 | 0.900 |
|  | Female | 12 | 15 | 27 |  |
| Age | <55 yrs | 9 | 5 | 14 | 0.096 |
|  | ≥55 yrs | 15 | 24 | 39 |  |

**p* values were analyzed by Chi-square test.

# According to the immunoreactive scores (IRS) from IHC of GBM tissue array: the cutoff between LOW and HIGH was set at the median IRS of GBM tissues.

**Supplemental Data 2. Association between TNFRSF1B expression and information of patients with GBM.**

| Status | | TNFRSF1B expression^#^, n | | Total | *p* value* |
| --- | --- | --- | --- | --- | --- |
|  |  | LOW | HIGH | n |  |
| Gender | Male | 12 | 14 | 26 | 0.884 |
|  | Female | 13 | 14 | 27 |  |
| Age | <55 yrs | 8 | 6 | 14 | 0.384 |
|  | ≥55 yrs | 17 | 22 | 39 |  |

**p* values were analyzed by Chi-square test.

# According to the immunoreactive scores (IRS) from IHC of GBM tissue array: the cutoff between LOW and HIGH was set at the median IRS of GBM tissues.

**Supplemental Data 3.** **Association between TRAF2 expression and information of patients with GBM.**

| Status | | TRAF2 expression^#^, n | | Total | *p* value* |
| --- | --- | --- | --- | --- | --- |
|  |  | LOW | HIGH | n |  |
| Gender | Male | 10 | 16 | 26 | 0.130 |
|  | Female | 16 | 11 | 27 |  |
| Age | <55 yrs | 7 | 7 | 14 | 0.934 |
|  | ≥55 yrs | 19 | 20 | 39 |  |

**p* values were analyzed by Chi-square test.

# According to the immunoreactive scores (IRS) from IHC of GBM tissue array: the cutoff between LOW and HIGH was set at the median IRS of GBM tissues.

**Supplemental Data 4. Association between FASN expression and information of patients with GBM.**

| Status | | FASN expression^#^, n | | Total | *p* value* |
| --- | --- | --- | --- | --- | --- |
|  |  | LOW | HIGH | n |  |
| Gender | Male | 11 | 15 | 26 | 0.335 |
|  | Female | 15 | 12 | 27 |  |
| Age | <55 yrs | 8 | 6 | 14 | 0.481 |
|  | ≥55 yrs | 18 | 21 | 39 |  |

**p* values were analyzed by Chi-square test.

# According to the immunoreactive scores (IRS) from IHC of GBM tissue array: the cutoff between LOW and HIGH was set at the median IRS of GBM tissues.
