# Supplementary material for: Low-dose TNF-α drives malignant progression and lipid metabolism in glioblastoma through the TRAF2-FASN axis
Source: Cell Death Discov. 2026 Apr 9;12:242. doi: 10.1038/s41420-026-03087-x (PMC13187350; doi:10.1038/s41420-026-03087-x)
Supplement: Supplementary file 3 — Supplementary Table 1 [file 41420_2026_3087_MOESM3_ESM.docx]

**Supplementary Table 1. Clinical pathological information of Gliomas.**

| **No.** | **Age** | **Gender** | **Disease specific survival in months** | **Overall survival (1=death, 0=alive)** | **IRS of TNFRSF1A** | **IRS of TNFRSF1B** | **IRS of TRAF2** | **IRS of FASN** |
| --- | --- | --- | --- | --- | --- | --- | --- | --- |
| 1 | 69 | Male | 3 | 1 | 12 | 8 | 12 | 12 |
| 2 | 73 | Female | 4 | 0 | 9 | 12 | 12 | 12 |
| 3 | 75 | Female | 6 | 1 | 9 | 9 | 9 | 9 |
| 4 | 36 | Male | 10 | 1 | 3 | 9 | 3 | 4 |
| 5 | 48 | Male | 1 | 1 | 9 | 12 | 9 | 12 |
| 6 | 71 | Female | 5 | 1 | 9 | 6 | 12 | 12 |
| 7 | 70 | Male | 7 | 1 | 9 | 8 | 12 | 9 |
| 8 | 66 | Female | 14 | 1 | 9 | 8 | 4 | 8 |
| 9 | 75 | Female | 12 | 1 | 9 | 6 | 9 | 9 |
| 10 | 75 | Female | 7 | 1 | 1 | 1 | 4 | 4 |
| 11 | 51 | Male | 62 | 1 | 1 | 2 | 3 | 3 |
| 12 | 71 | Male | 9 | 1 | 9 | 9 | 9 | 4 |
| 13 | 57 | Female | 4 | 1 | 4 | 4 | 6 | 6 |
| 14 | 73 | Female | 13 | 1 | 6 | 9 | 4 | 3 |
| 15 | 54 | Female | 2 | 1 | 4 | 1 | 3 | 8 |
| 16 | 69 | Male | 48 | 1 | 4 | 6 | 9 | 9 |
| 17 | 65 | Male | 8 | 1 | 3 | 12 | 4 | 4 |
| 18 | 44 | Female | 21 | 1 | 6 | 9 | 6 | 4 |
| 19 | 55 | Male | 6 | 0 | 12 | 9 | 9 | 12 |
| 20 | 53 | Male | 15 | 1 | 6 | 6 | 4 | 4 |
| 21 | 62 | Male | 12 | 1 | 8 | 6 | 6 | 8 |
| 22 | 60 | Female | 7 | 1 | 9 | 8 | 3 | 8 |
| 23 | 70 | Female | 5 | 1 | 8 | 6 | 9 | 9 |
| 24 | 70 | Male | 2 | 1 | 12 | 9 | 9 | 9 |
| 25 | 43 | Female | 11 | 1 | 1 | 2 | 12 | 4 |
| 26 | 33 | Female | 18 | 1 | 2 | 6 | 3 | 4 |
| 27 | 61 | Male | 16 | 1 | 9 | 12 | 4 | 9 |
| 28 | 52 | Male | 2 | 1 | 6 | 8 | 9 | 8 |
| 29 | 73 | Female | 16 | 1 | 8 | 9 | 9 | 3 |
| 30 | 57 | Male | 56 | 0 | 4 | 2 | 12 | 9 |
| 31 | 65 | Male | 23 | 1 | 2 | 3 | 2 | 3 |
| 32 | 72 | Female | 18 | 1 | 4 | 8 | 4 | 4 |
| 33 | 61 | Female | 12 | 1 | 12 | 9 | 9 | 9 |
| 34 | 76 | Male | 4 | 1 | 6 | 8 | 9 | 9 |
| 35 | 73 | Male | 14 | 1 | 9 | 8 | 6 | 6 |
| 36 | 65 | Male | 12 | 1 | 8 | 6 | 9 | 8 |
| 37 | 69 | Male | 5 | 1 | 9 | 12 | 12 | 12 |
| 38 | 42 | Female | 4 | 1 | 9 | 9 | 9 | 12 |
| 39 | 54 | Female | 2 | 1 | 12 | 12 | 12 | 9 |
| 40 | 53 | Female | 109 | 0 | 4 | 1 | 1 | 2 |
| 41 | 55 | Female | 14 | 1 | 8 | 4 | 6 | 4 |
| 42 | 32 | Male | 6 | 0 | 9 | 4 | 9 | 8 |
| 43 | 74 | Male | 9 | 1 | 12 | 9 | 12 | 12 |
| 44 | 65 | Male | 7 | 1 | 3 | 4 | 12 | 4 |
| 45 | 55 | Female | 12 | 1 | 4 | 1 | 2 | 6 |
| 46 | 67 | Male | 60 | 0 | 1 | 2 | 2 | 2 |
| 47 | 62 | Female | 15 | 1 | 8 | 9 | 4 | 4 |
| 48 | 67 | Male | 15 | 1 | 1 | 3 | 4 | 3 |
| 49 | 67 | Female | 60 | 0 | 2 | 3 | 2 | 6 |
| 50 | 63 | Female | 17 | 1 | 9 | 9 | 3 | 6 |
| 51 | 56 | Female | 2 | 1 | 9 | 8 | 8 | 9 |
| 52 | 71 | Female | 1 | 1 | 3 | 2 | 6 | 6 |
| 53 | 52 | Male | 26 | 1 | 8 | 1 | 8 | 6 |

**Inclusion Criteria:** This study consecutively enrolled patients aged between 18 and 75 years, with all specimens pathologically confirmed. All patients underwent their first surgical resection without prior radiotherapy or chemotherapy and were able to provide complete clinical data and informed consent.

**Exclusion Criteria:** Patients were excluded if they met any of the following conditions: diagnosed with recurrent or metastatic brain tumors; had a history of other malignant tumors; suffered from severe systemic diseases; had incomplete clinical data or were lost to follow-up; or if their tissue samples were of inadequate quality for analysis.
