# Supplementary material for: Low-dose TNF-α drives malignant progression and lipid metabolism in glioblastoma through the TRAF2-FASN axis
Source: Cell Death Discov. 2026 Apr 9;12:242. doi: 10.1038/s41420-026-03087-x (PMC13187350; doi:10.1038/s41420-026-03087-x)
Supplement: Supplementary file 4 — Supplementary Table 2 [file 41420_2026_3087_MOESM4_ESM.docx]

**Supplementary Table 2. The antibodies and dilution used to detect the indicated proteins.**

| **Antigen** | **Primary Antibody** | **Dilution** |
| --- | --- | --- |
| TRAF2 | Cell Signaling Technology; 4724; rabbit monoclonal | 1:1000 for WB; 1:100 for IP |
| TRAF2 | Anfitty;AF5382;rabbit monoclonal | 1:100 for IF/IHC |
| FASN | Cell Signaling Technology; 4724; rabbit monoclonal | 1:1000 for WB; 1:100 for IP;1:50 for IF |
| FASN | Proteintech; 66591-1-Ig;mouse monoclonal | 1:400 for IF/IHC |
| β-actin | Proteintech; 66009-1-Ig;mouse monoclonal | 1:5000 for WB |
| HA-Tag | Abcam; ab9110; rabbit polyclonal | 1:5000 for WB; 1:200 for IP |
| Flag-Tag | Abmart; M20008; mouse monoclonal | 1:5000 for WB; 1:200 for IP |
| His-Tag | Proteintech; 66005-1-Ig; mouse monoclonal | 1:5000 for WB; 1:200 for IP;1:400 for IF |
| Ubiquitin | Cell Signaling Technology; 3639; mouse monoclonal | 1:1000 for WB |
| K48 | Cell Signaling Technology; 8081; rabbit monoclonal | 1:1000 for WB |
| K63 | Cell Signaling Technology; 5621; rabbit monoclonal | 1:1000 for WB |
| Normal Rabbit IgG | Abcam; ab172730; rabbit monoclonal | 1:100 for IP |
| TNFRSF1A | Proteintech; 21574-1-AP; rabbit polyclonal | 1:1000 for IHC |
| TNFRSF1B | Proteintech; 28746-1-AP; rabbit polyclonal | 1:50 for IHC |
| TNF alpha | Affinity;AF7014;rabbit polyclonal | 1:50 for IHC |
