# Supplementary material for: Low-dose TNF-α drives malignant progression and lipid metabolism in glioblastoma through the TRAF2-FASN axis
Source: Cell Death Discov. 2026 Apr 9;12:242. doi: 10.1038/s41420-026-03087-x (PMC13187350; doi:10.1038/s41420-026-03087-x)
Supplement: Supplementary file 5 — Supplementary Table 3 [file 41420_2026_3087_MOESM5_ESM.docx]

**Supplementary Table 3. Lists of target sequences of shRNA used in this study.**

| **shRNA** | **Vector** | **Target sequences** |
| --- | --- | --- |
| *sh-TRAF2#1* | PRRLSIN-CPPT-U6-ShRNA-SFFV-EGFP-SV40-puromycin | CCCTTGCAGATTCCACGCCAT |
| *sh-TRAF2#2* | PRRLSIN-CPPT-U6-ShRNA-SFFV-EGFP-SV40-puromycin | CTGGACCAAGACAAGATTGAA |
| *sh-FASN#1* | hU6-MCS-CBh-gcGFP-IRES-puromycin | CATGGAGCGTATCTGTGAGAA |
| *sh-FASN#2* | hU6-MCS-CBh-gcGFP-IRES-puromycin | CCTACTGGATGCGTTCTTCAA |
