# Supplementary material for: Low-dose TNF-α drives malignant progression and lipid metabolism in glioblastoma through the TRAF2-FASN axis
Source: Cell Death Discov. 2026 Apr 9;12:242. doi: 10.1038/s41420-026-03087-x (PMC13187350; doi:10.1038/s41420-026-03087-x)
Supplement: Supplementary file 6 — Supplementary Table 4 [file 41420_2026_3087_MOESM6_ESM.docx]

**Supplementary Table4. The sequences of the primers used in the study.**

| **Primer** | **Forward(5’-3’)** | **Reverse(5’-3’)** |
| --- | --- | --- |
| *TRAF2* | GCCCTTCAACCAGAAGGTGAC | CCAACCCCCAGACACCAGTA |
| *FASN* | CCTGGCTGCCTACTACATCG | CACATTTCAAAGGCCACGCA |
| *β-actin* | CATGTACGTTGCTATCCAGGC | CTCCTTAATGTCACGCACGAT |
