# Supplementary material for: Low-dose TNF-α drives malignant progression and lipid metabolism in glioblastoma through the TRAF2-FASN axis
Source: Cell Death Discov. 2026 Apr 9;12:242. doi: 10.1038/s41420-026-03087-x (PMC13187350; doi:10.1038/s41420-026-03087-x)

Original western blots

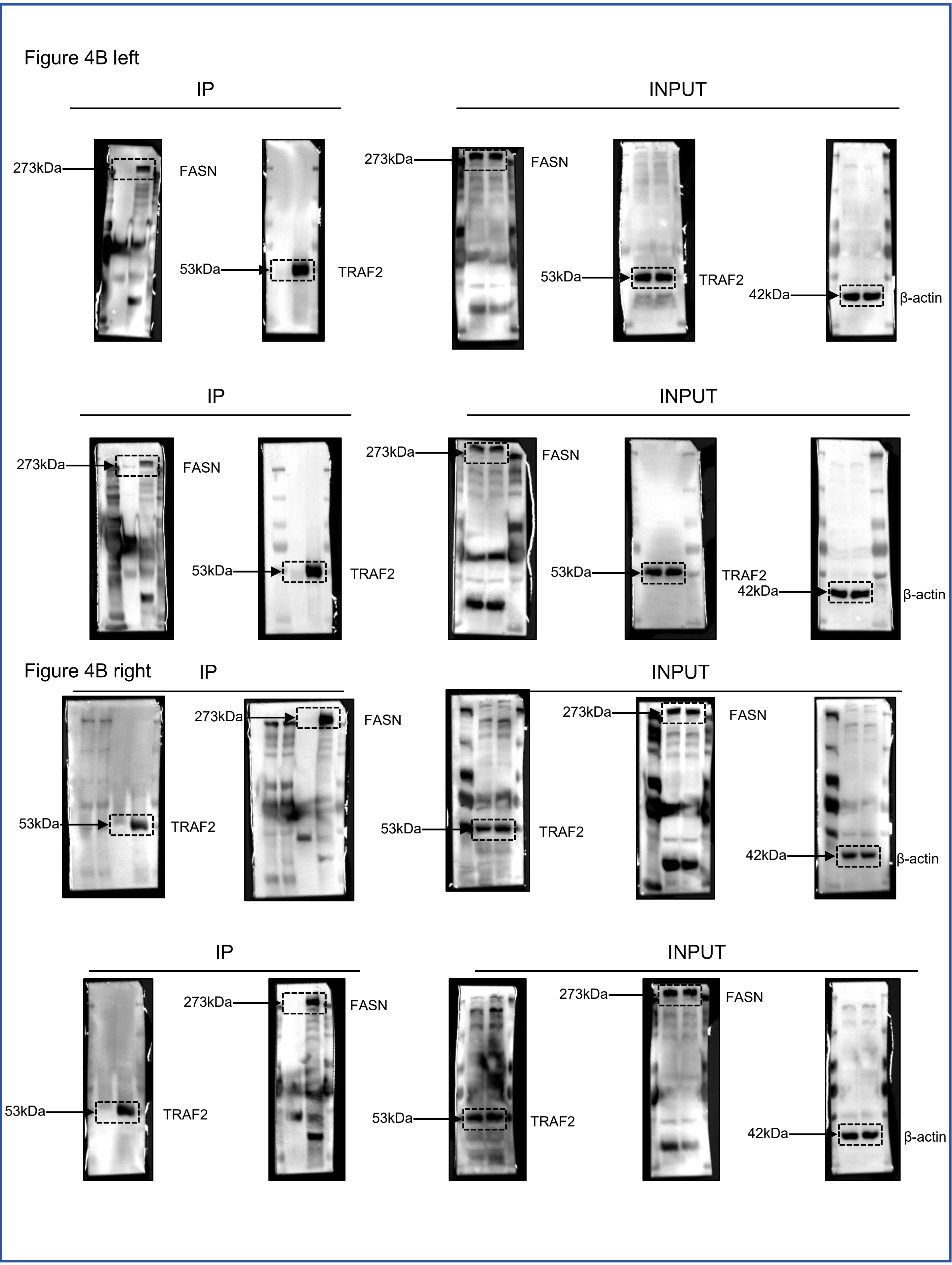

Figure 4C left

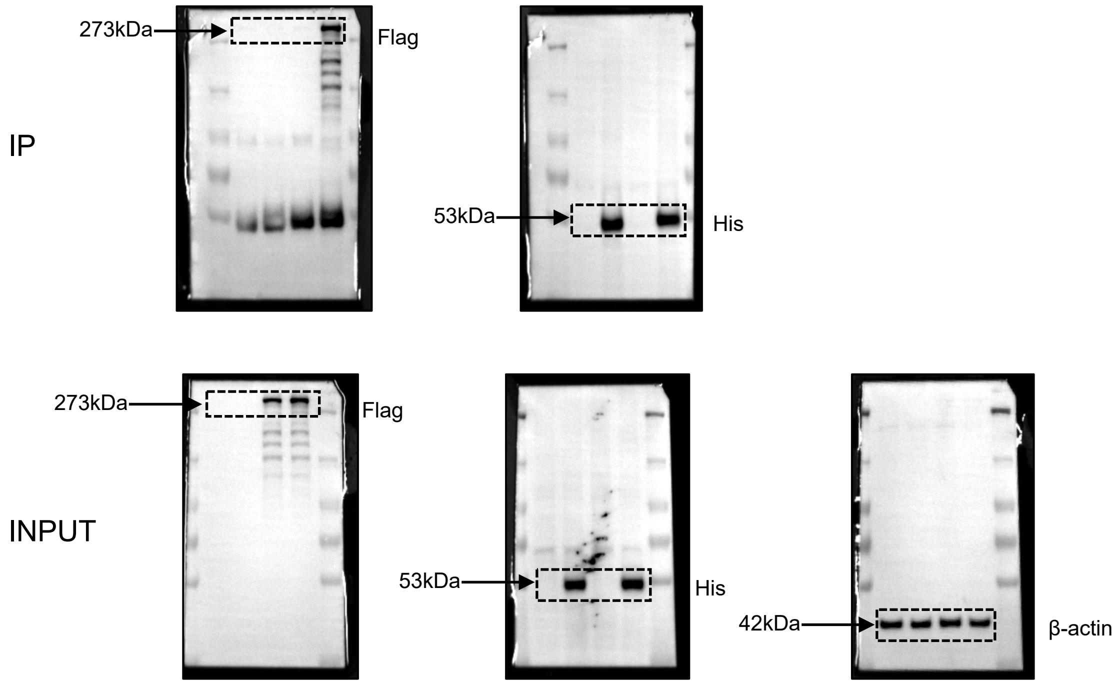

Figure 4C right

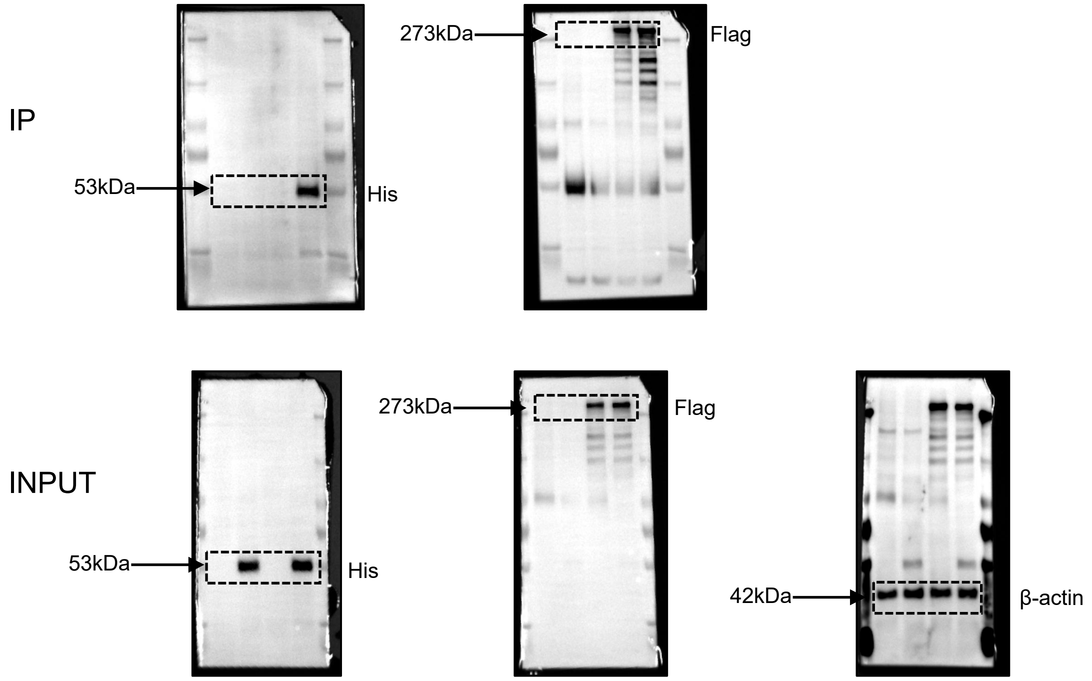

Figure 4E U251

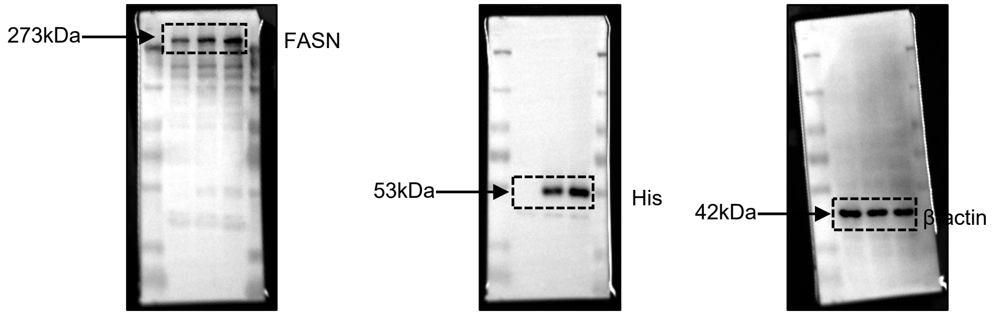

Figure 4E A172

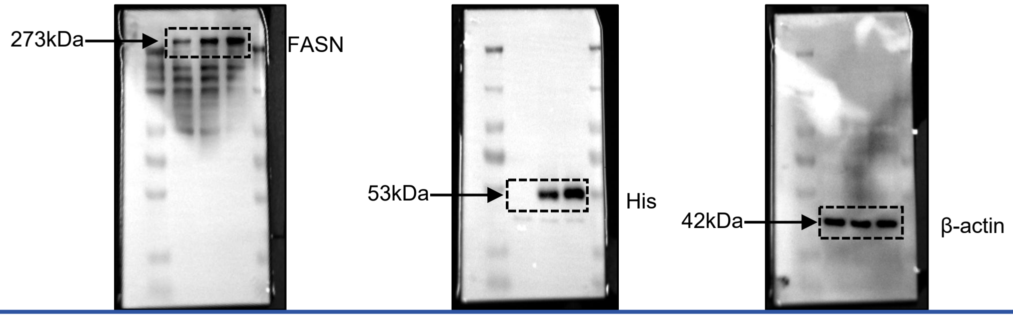

Figure 4F U251

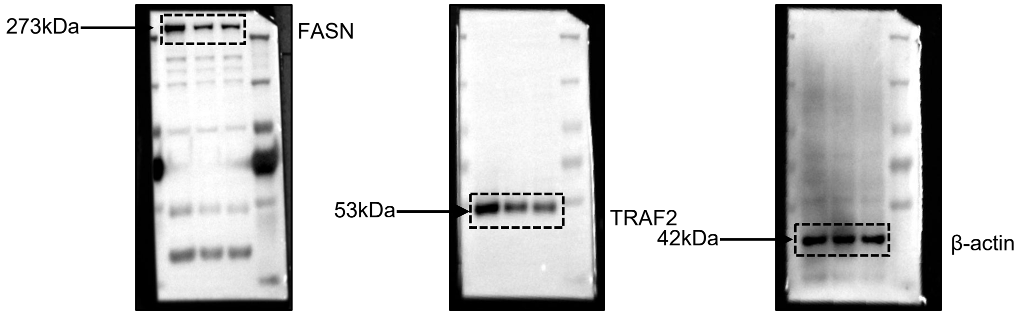

Figure 4F A172

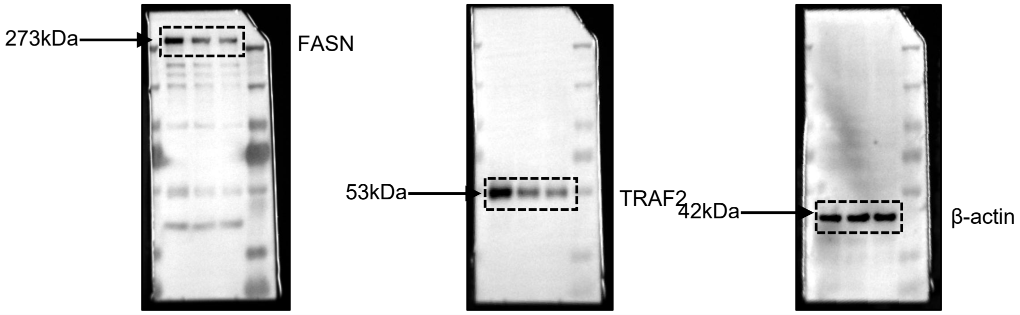

Figure 4G

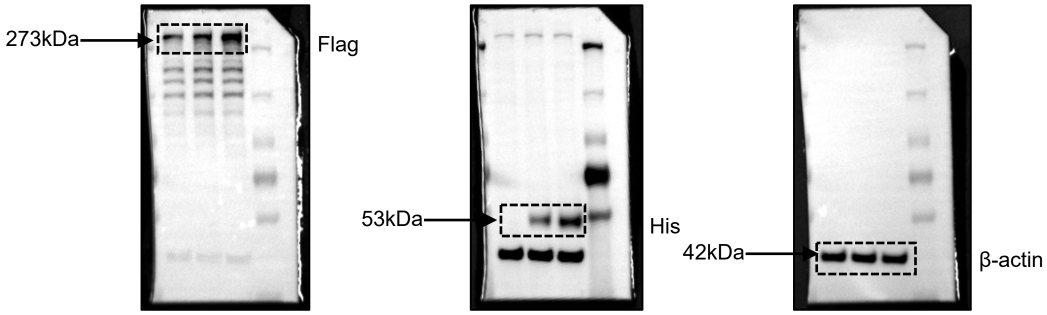

Figure 4I U251

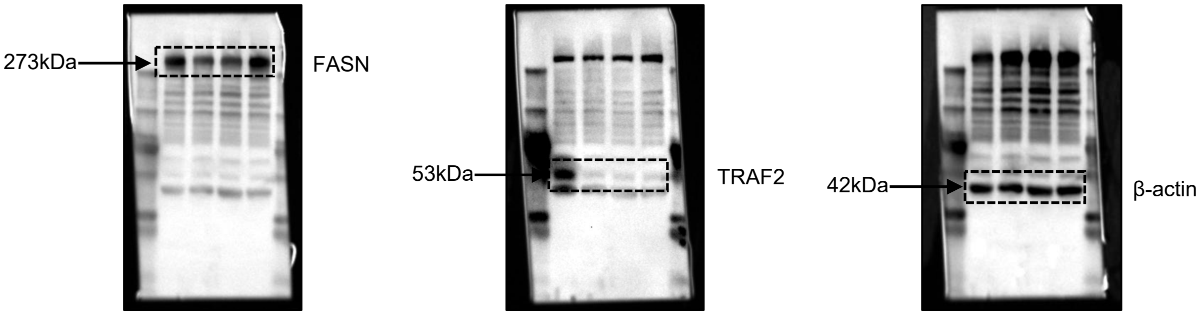

Figure 4I A172

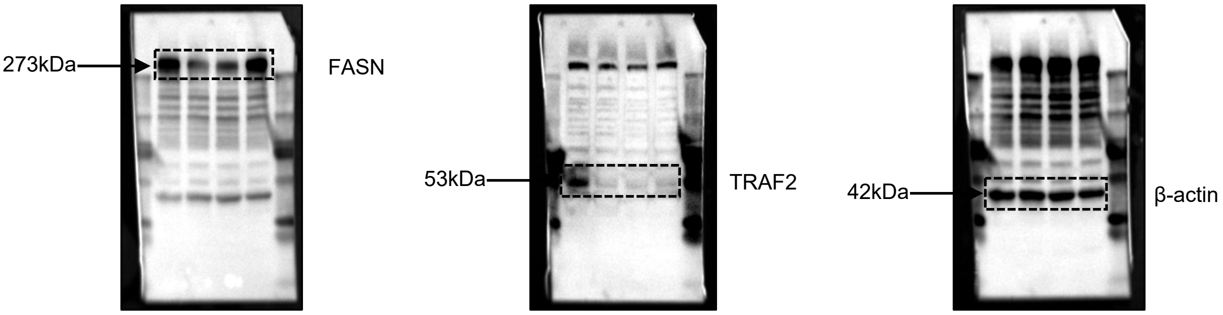

Figure 4K U251

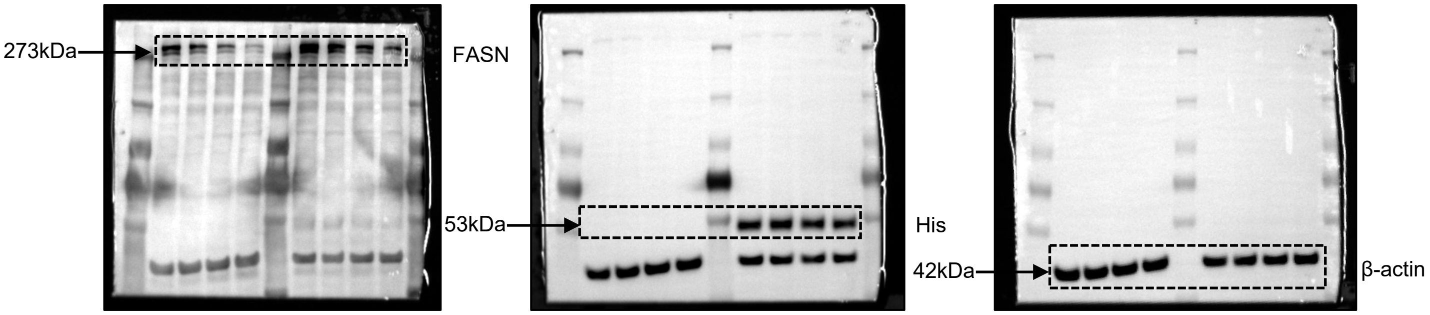

Figure 4K A172

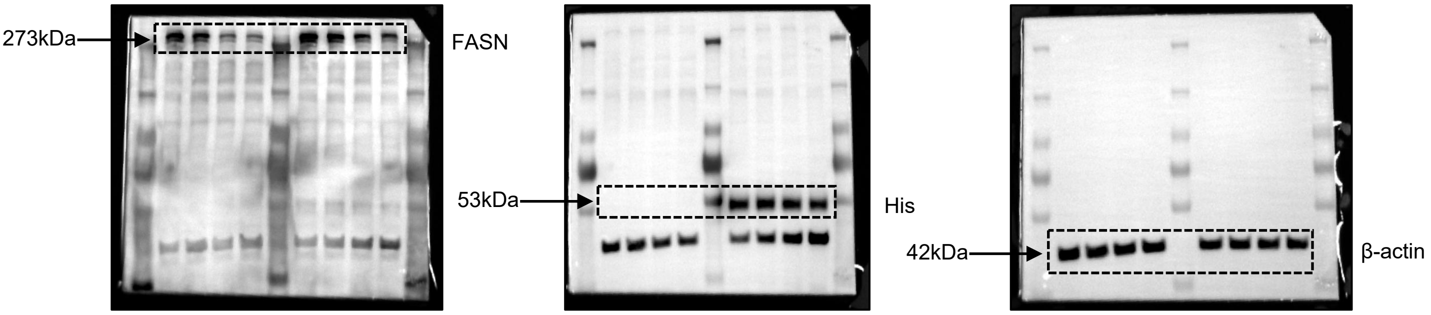

Figure 4L U251

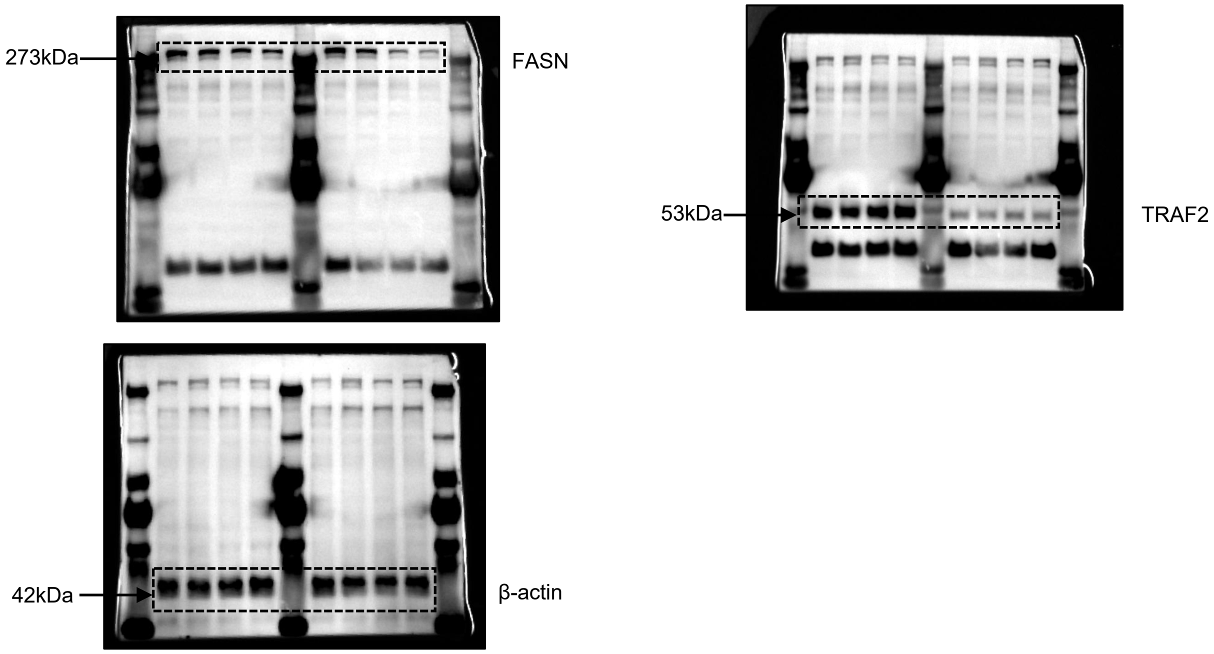

Figure 4L A172

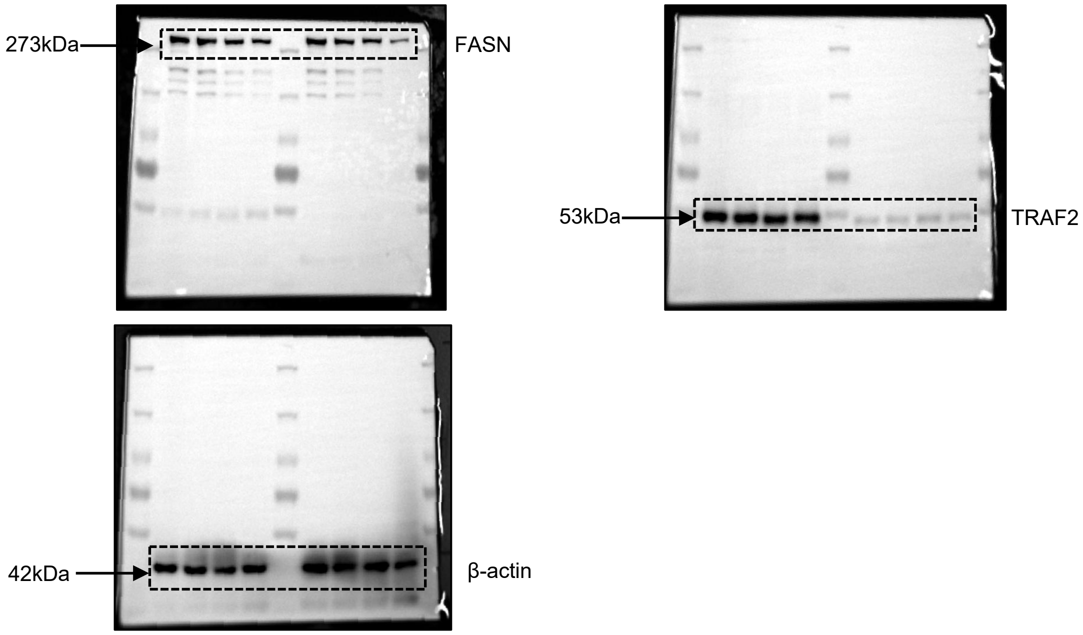

Figure 5A left

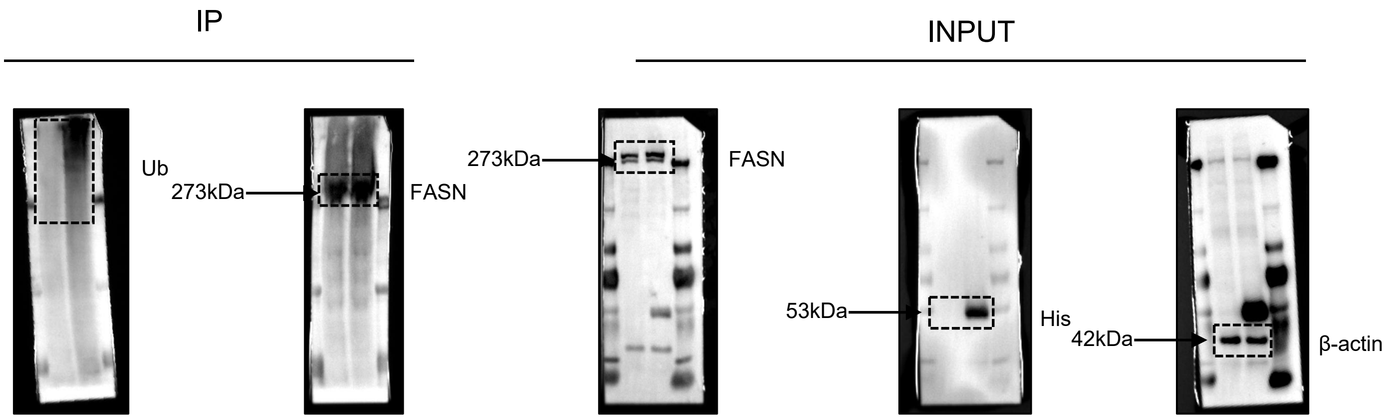

Figure 5A right

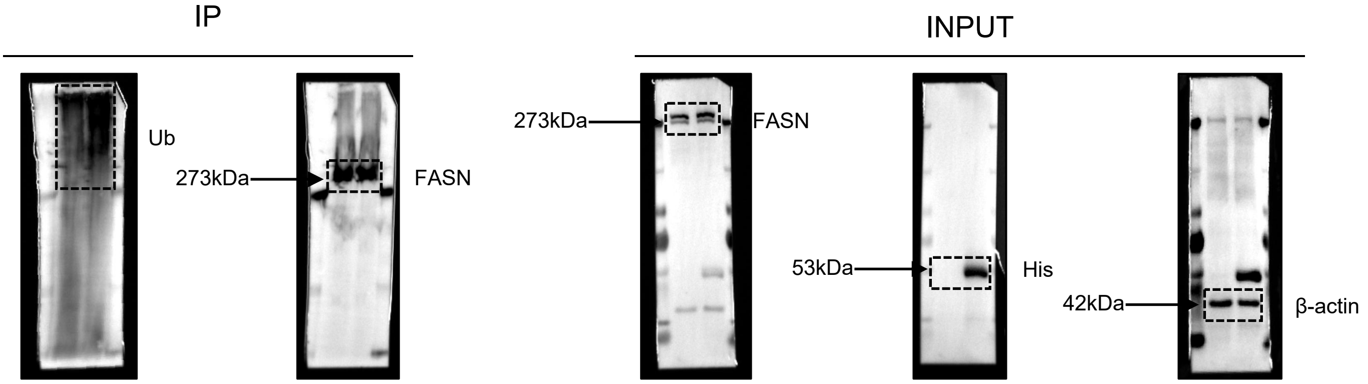

Figure 5B left

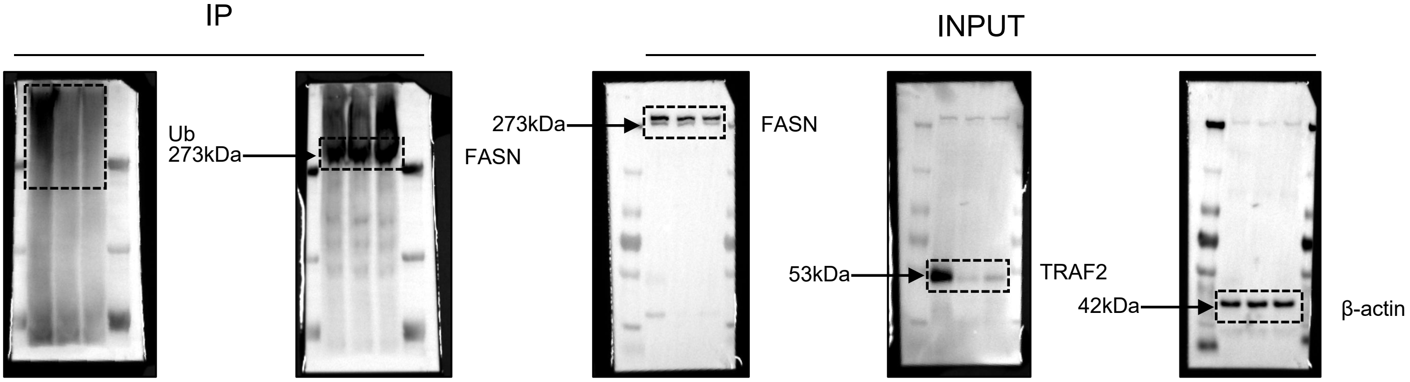

Figure 5B right

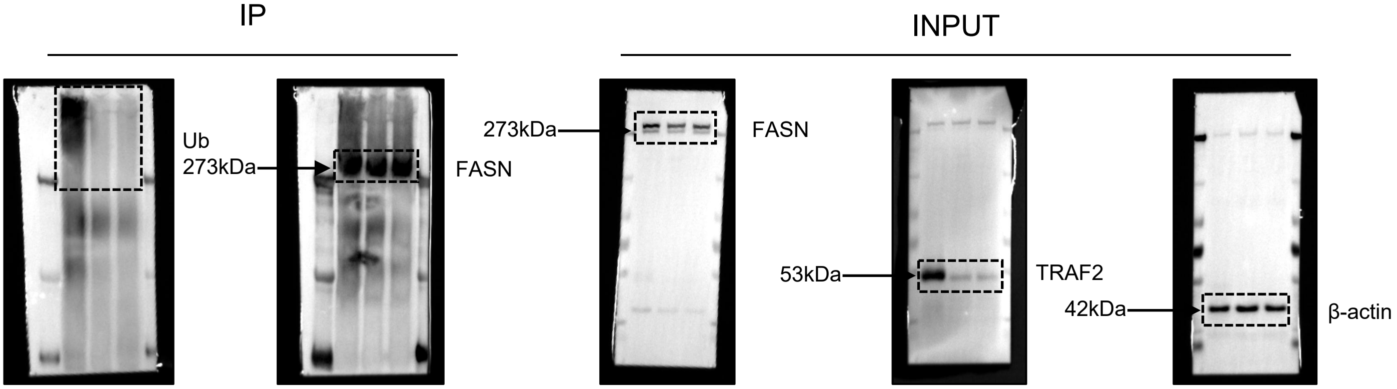

Figure 5C

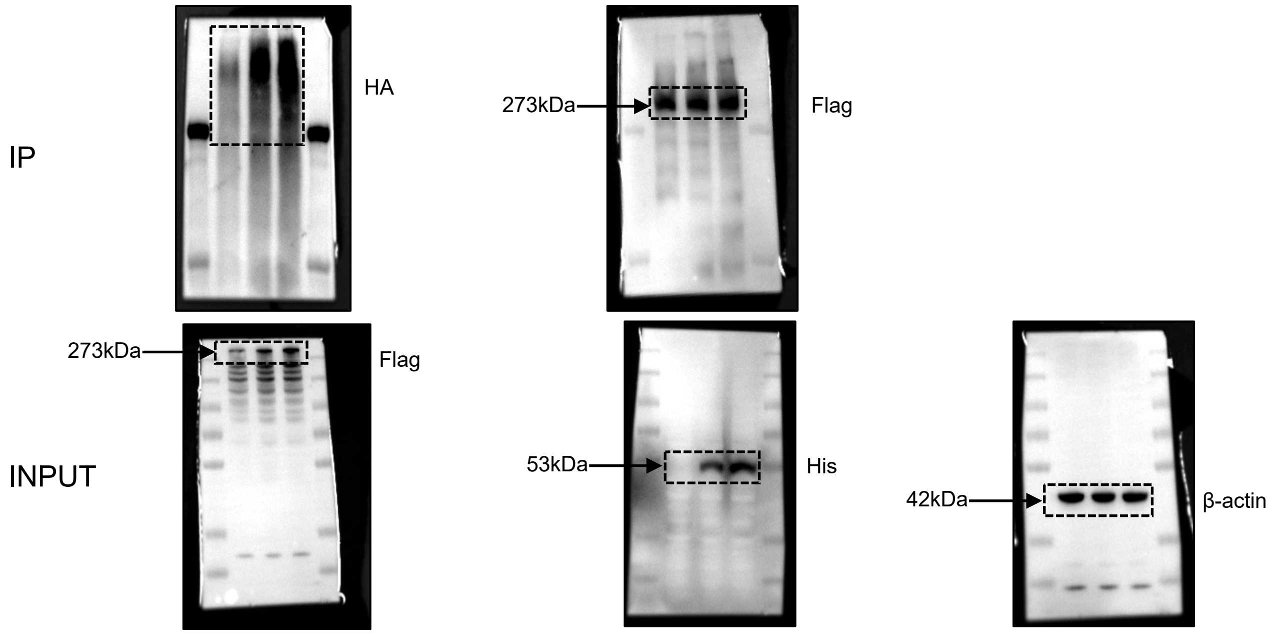

Figure 5D left

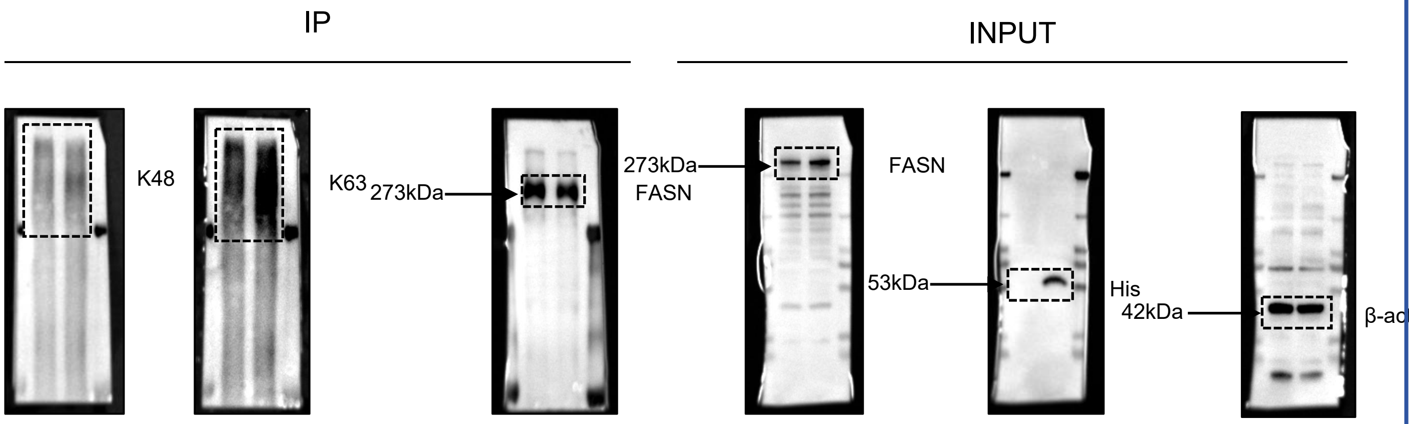

Figure 5D right

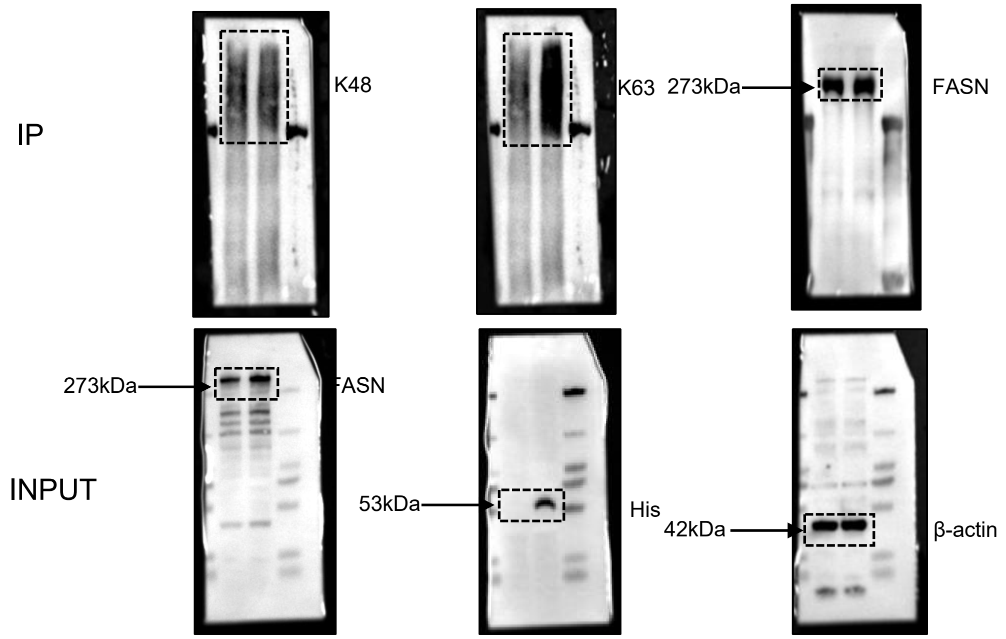

Figure 5E left

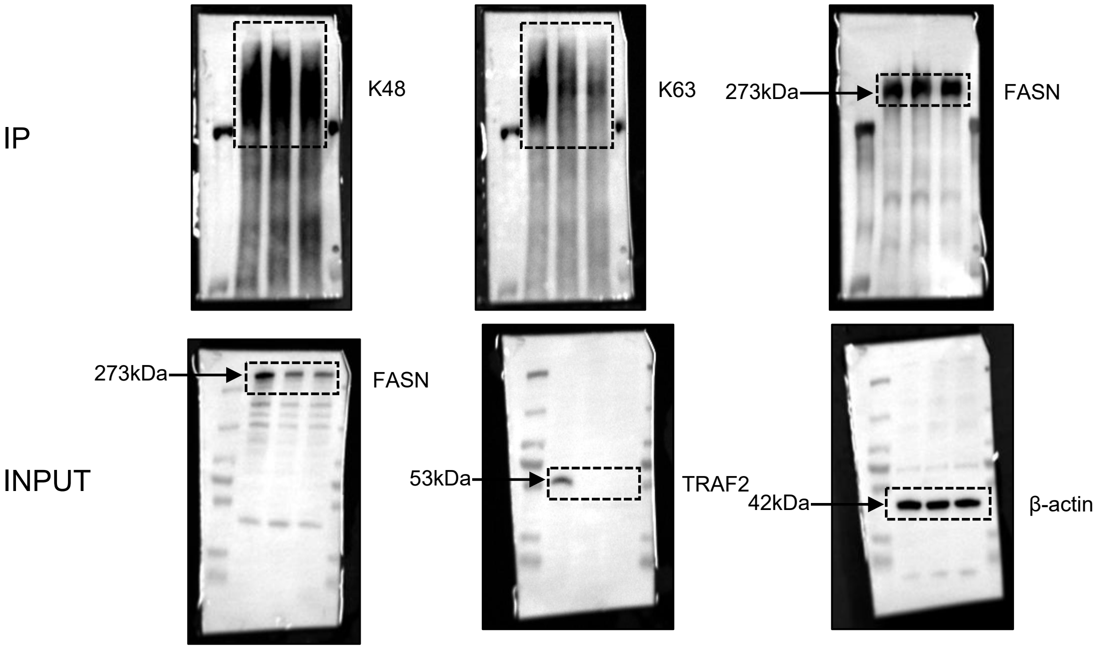

Figure 5E right

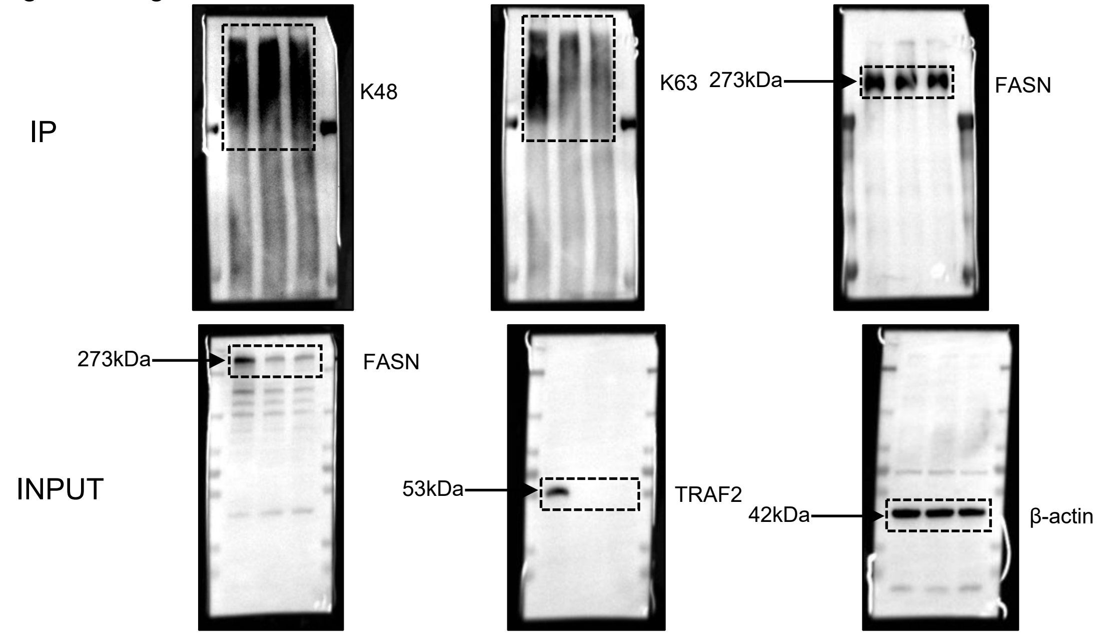

Figure 5F

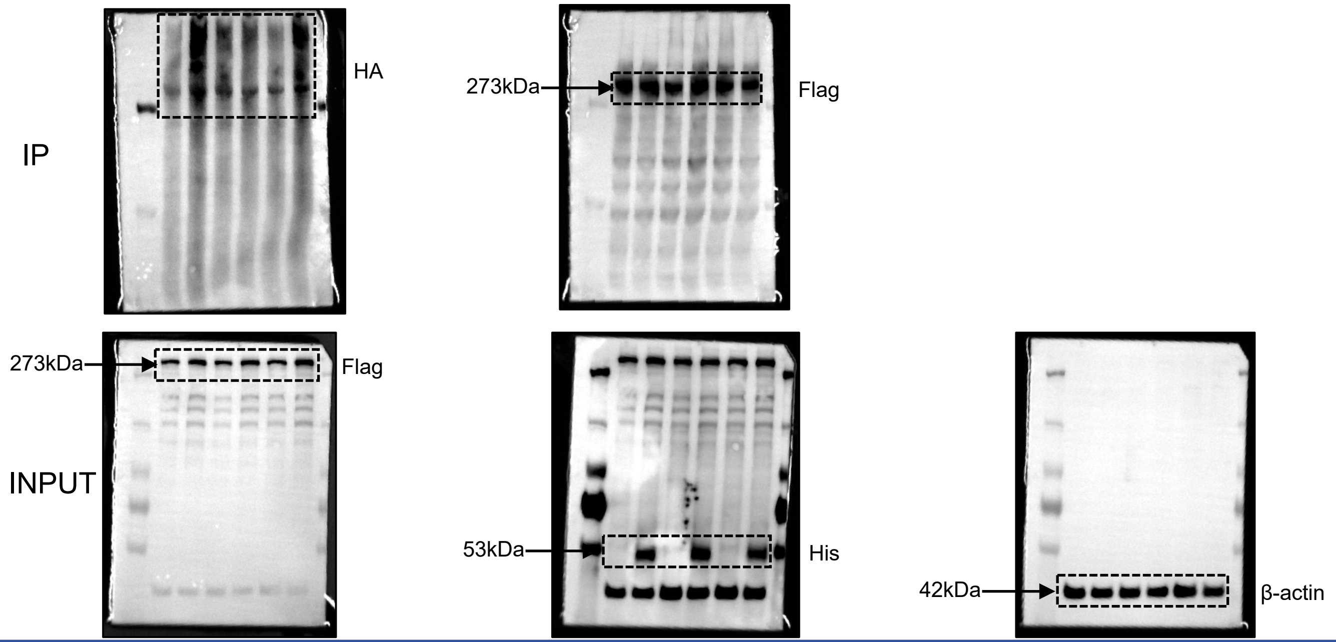

Figure 5G

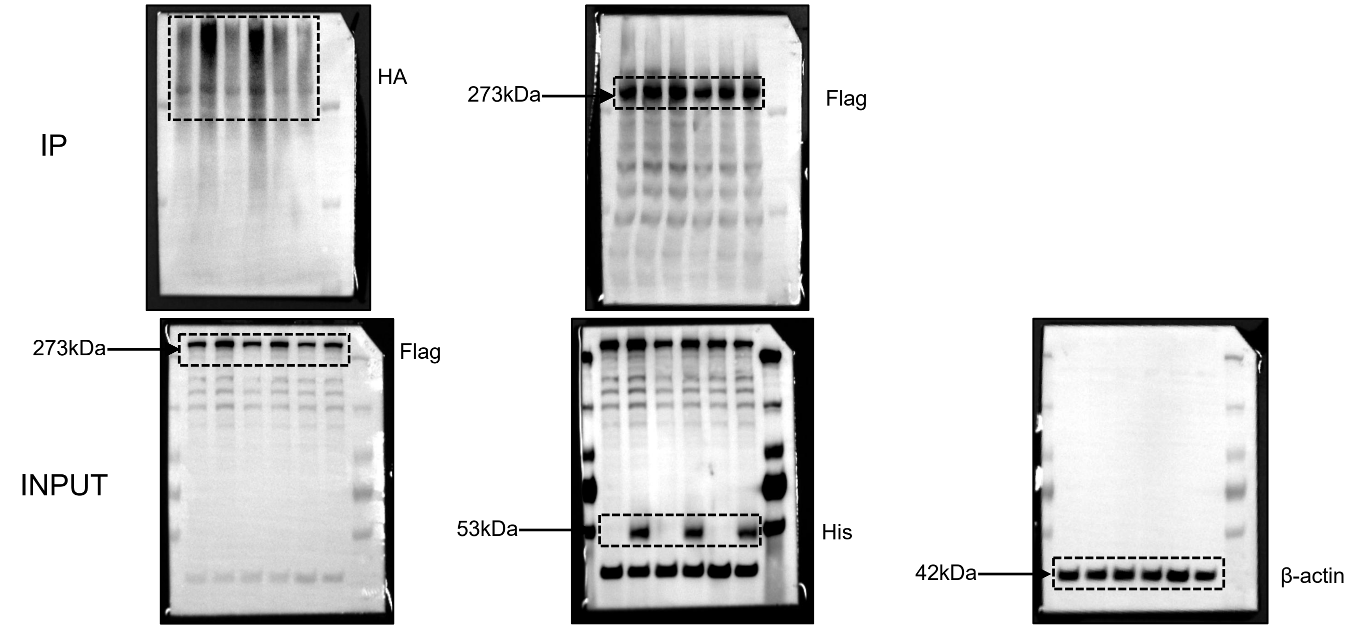

Figure 5H

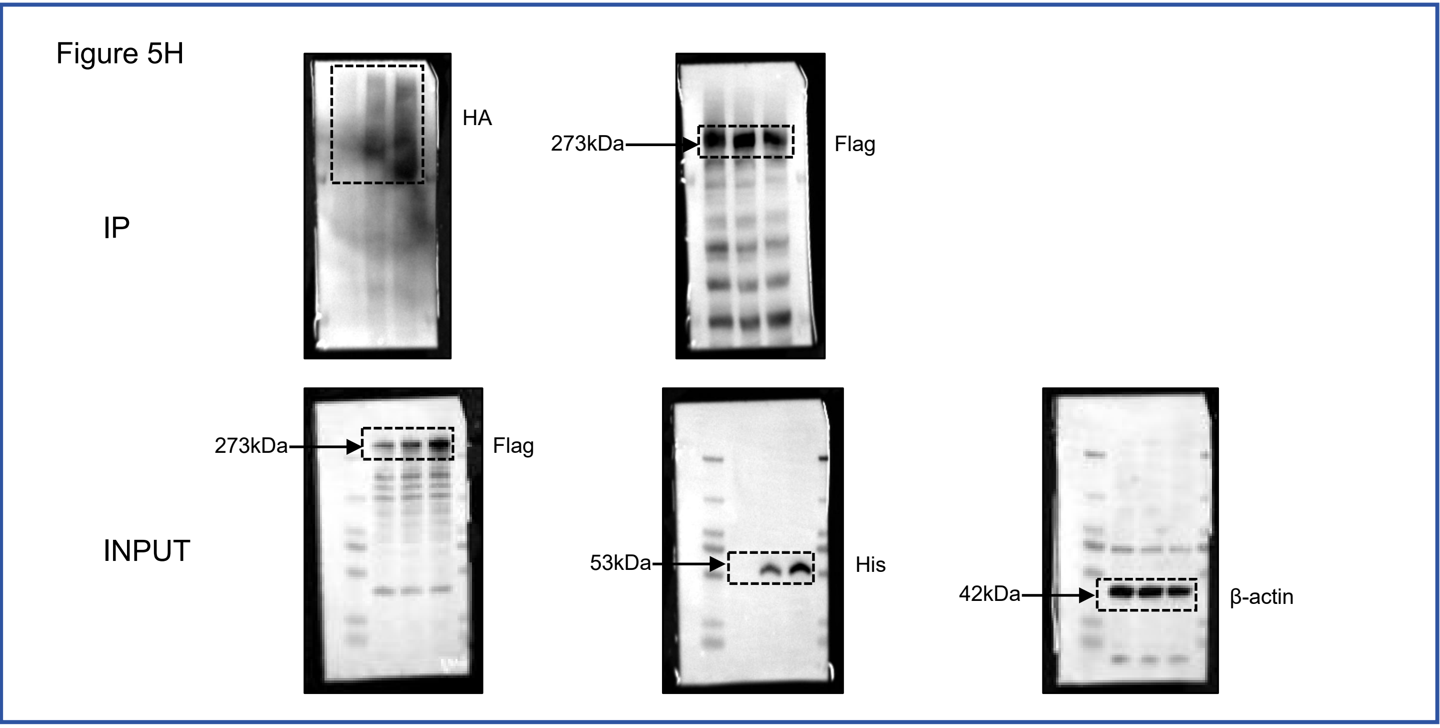

Figure 5I

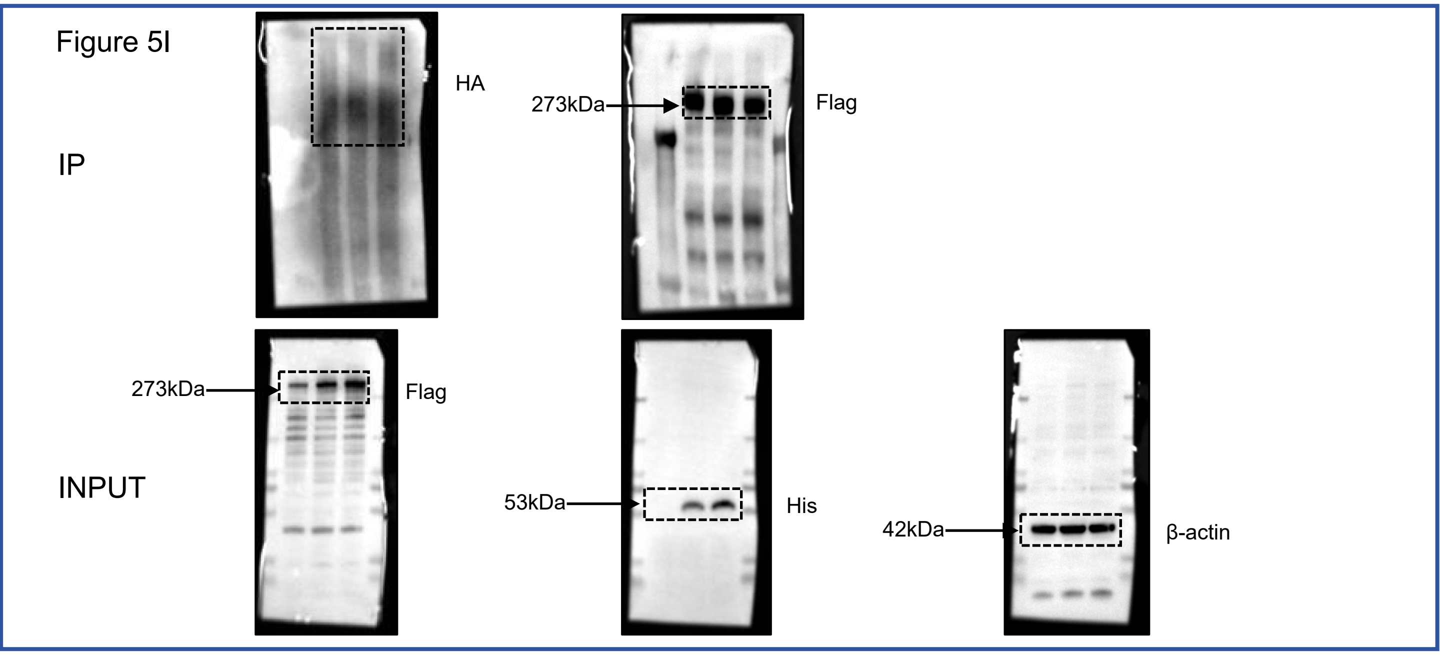

Figure 5J left

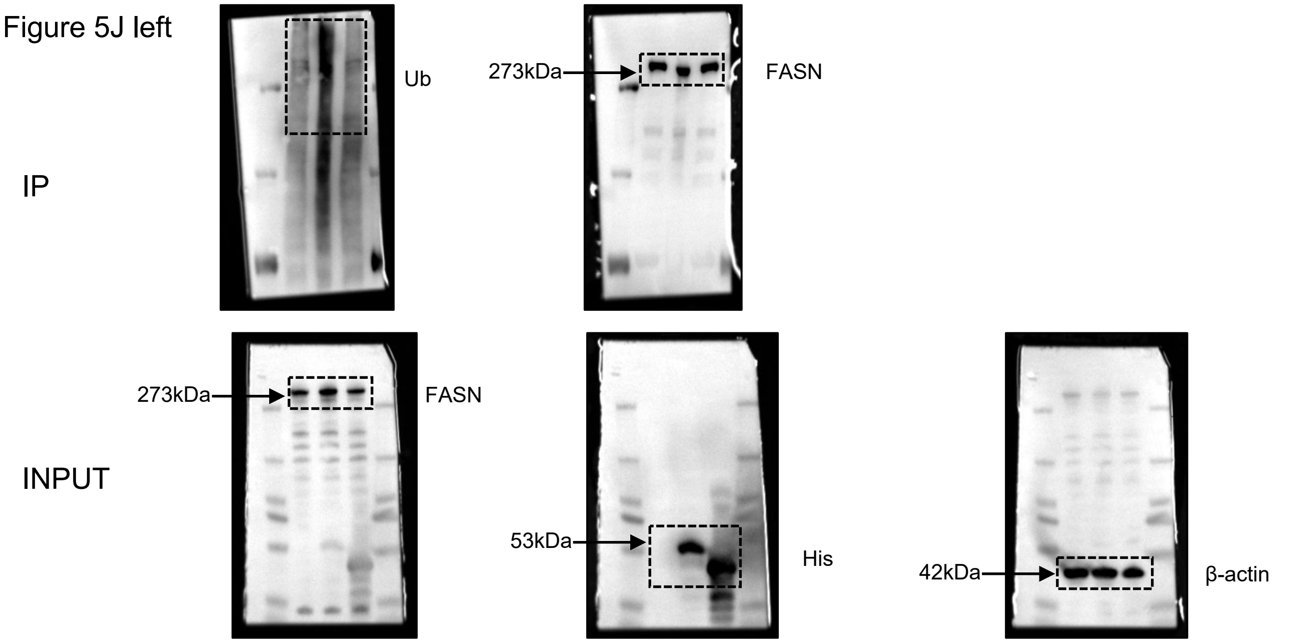

Figure 5J right

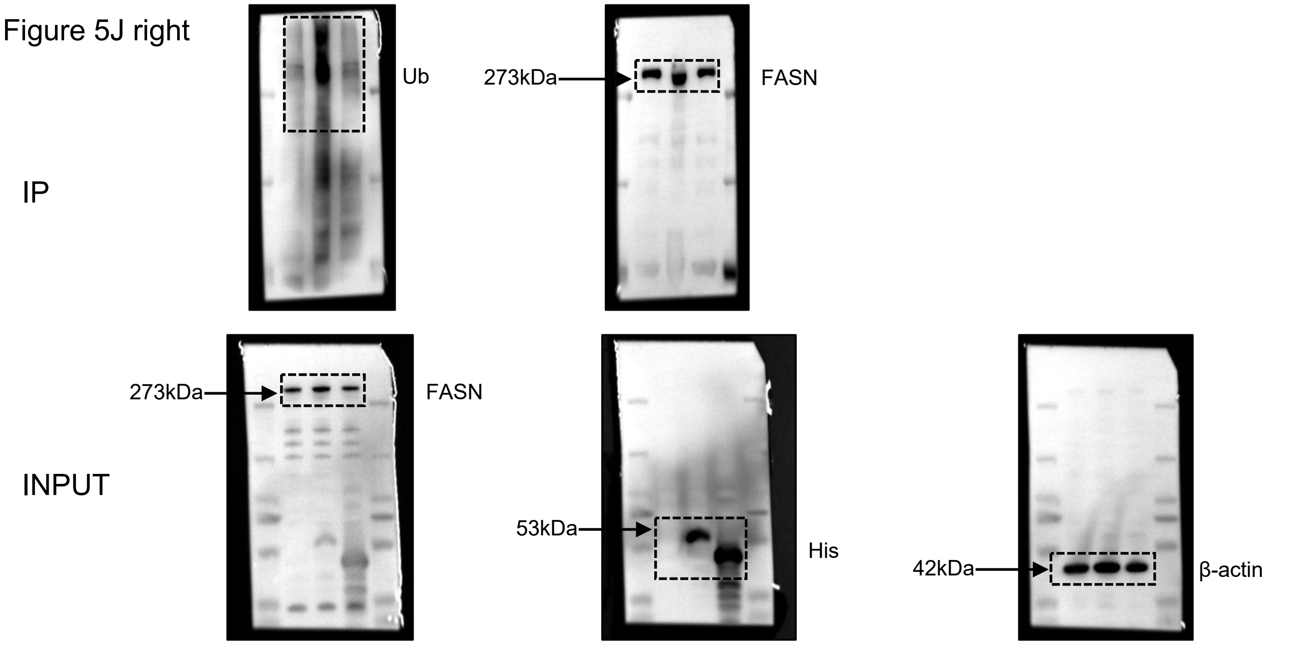

Figure 5K

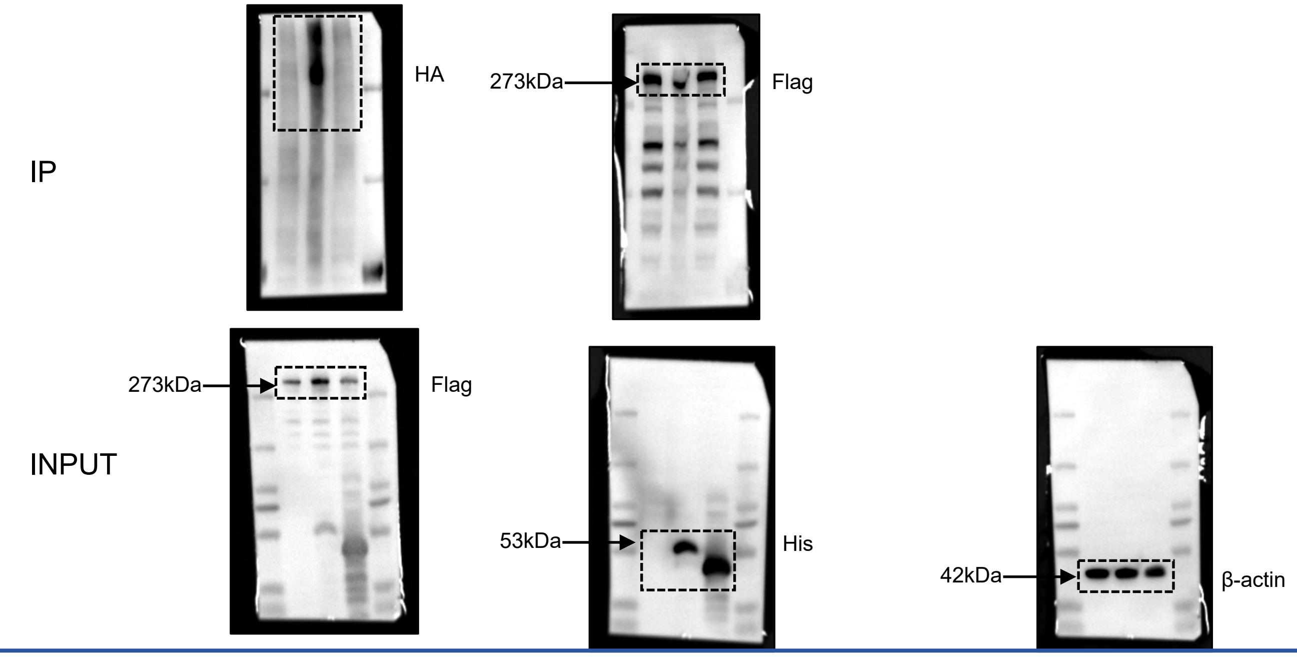

Figure 6A U251

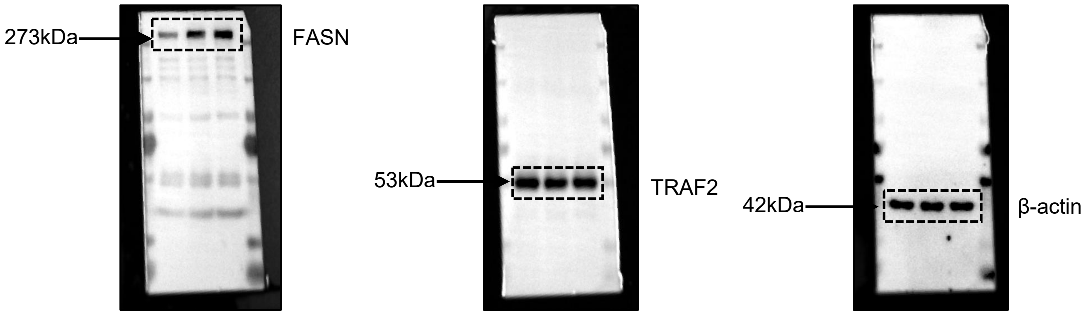

Figure 6A A172

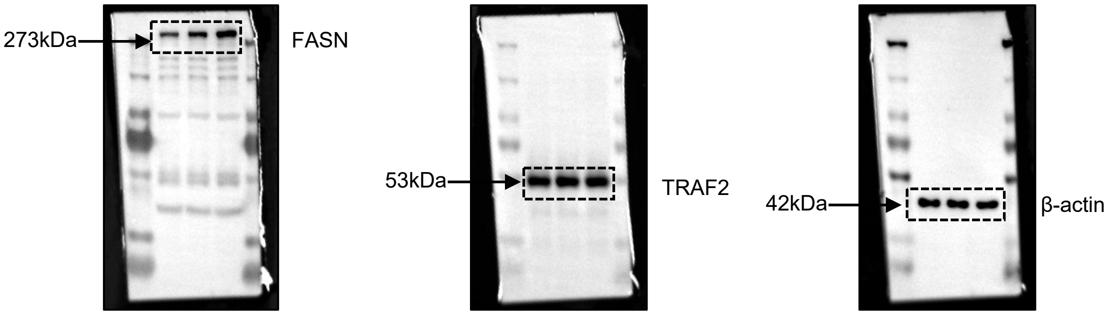

Figure 6B U251

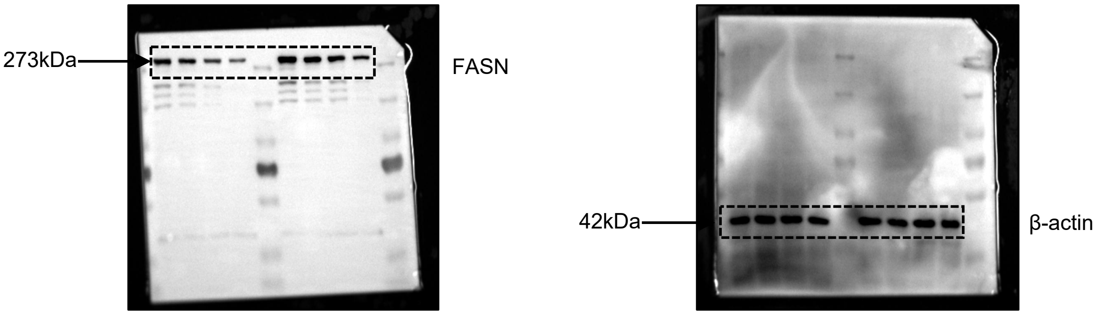

Figure 6B A172

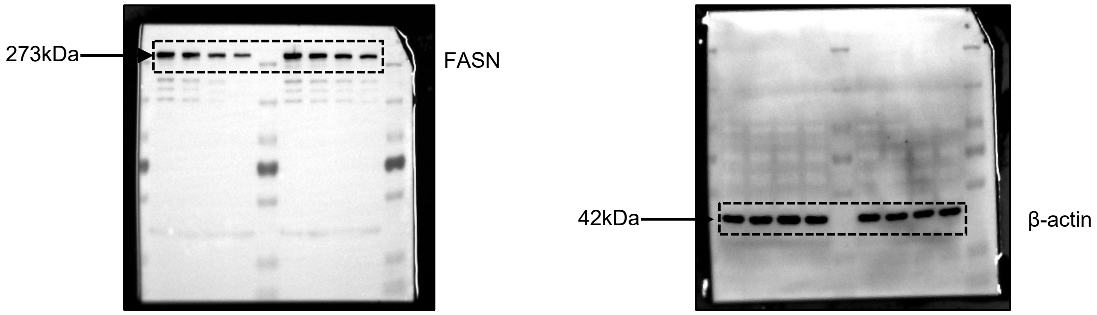

Figure 6C U251

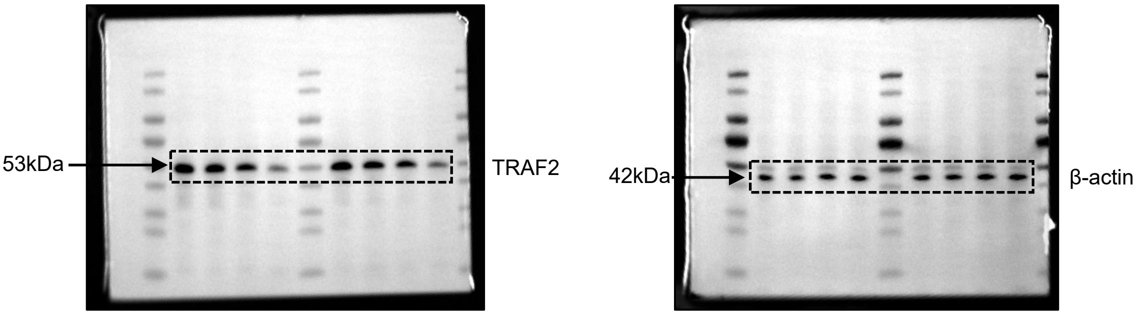

Figure 6C A172

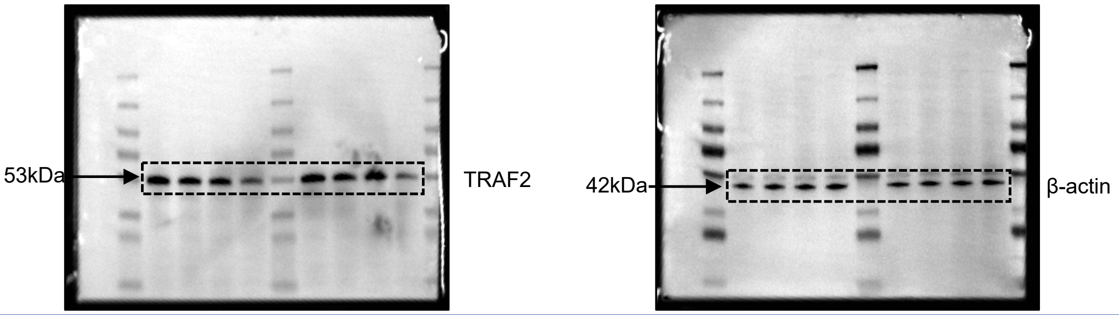

Figure 6E U251

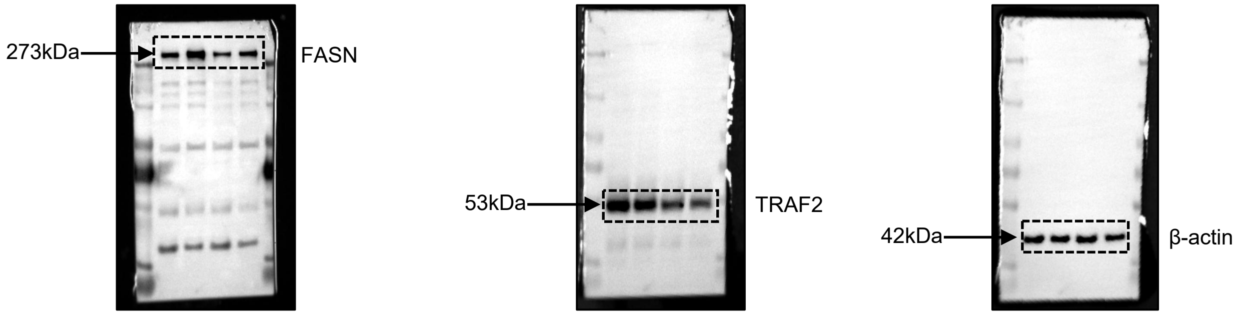

Figure 6E A172

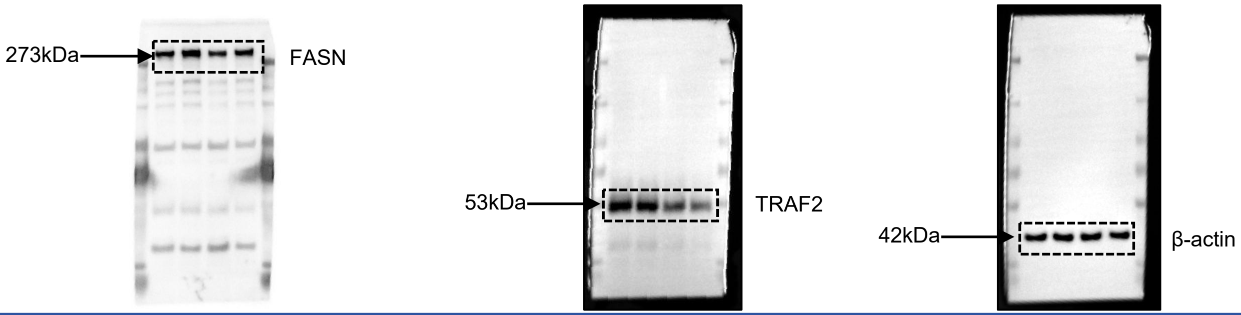

Figure 6F left

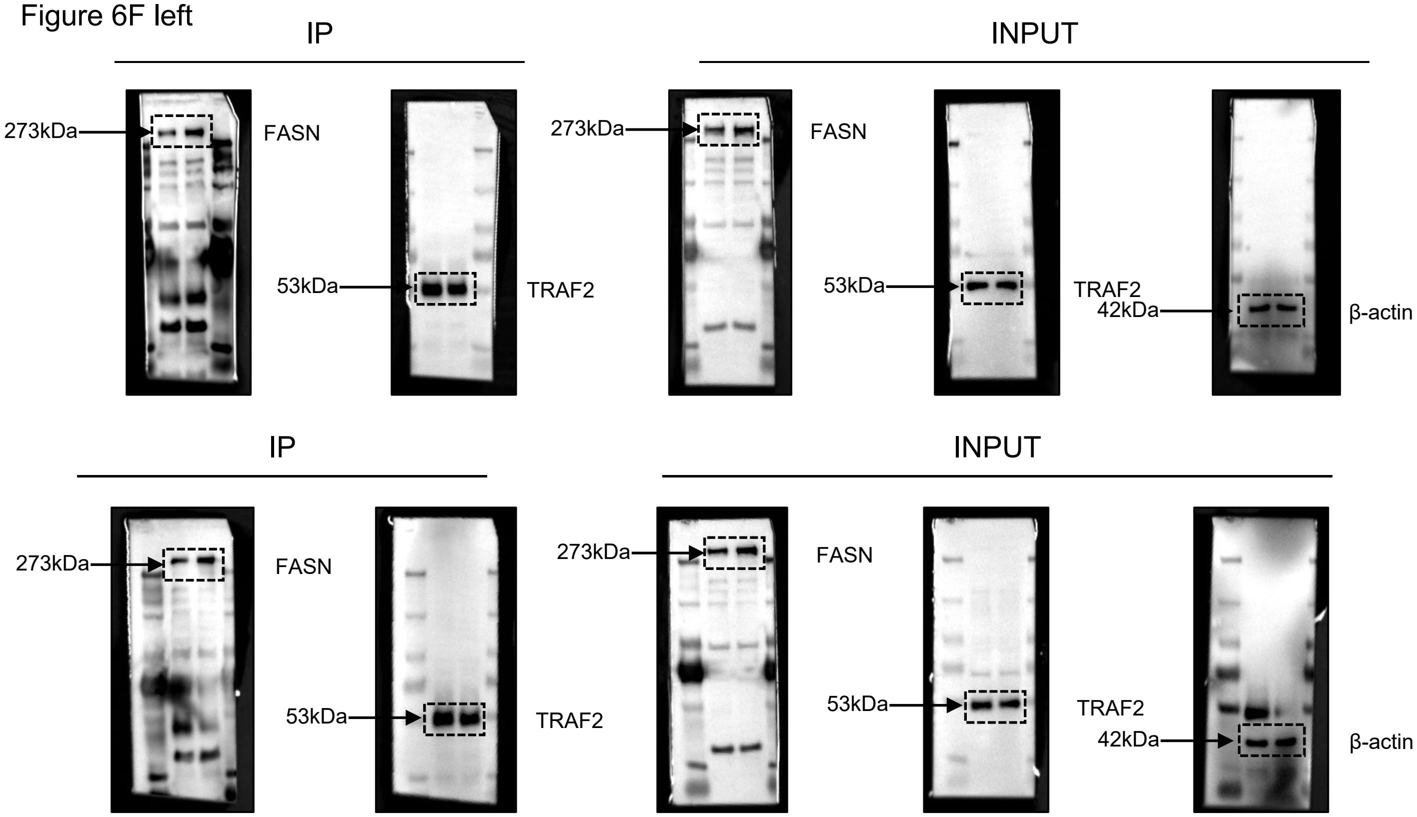

Figure 6F right

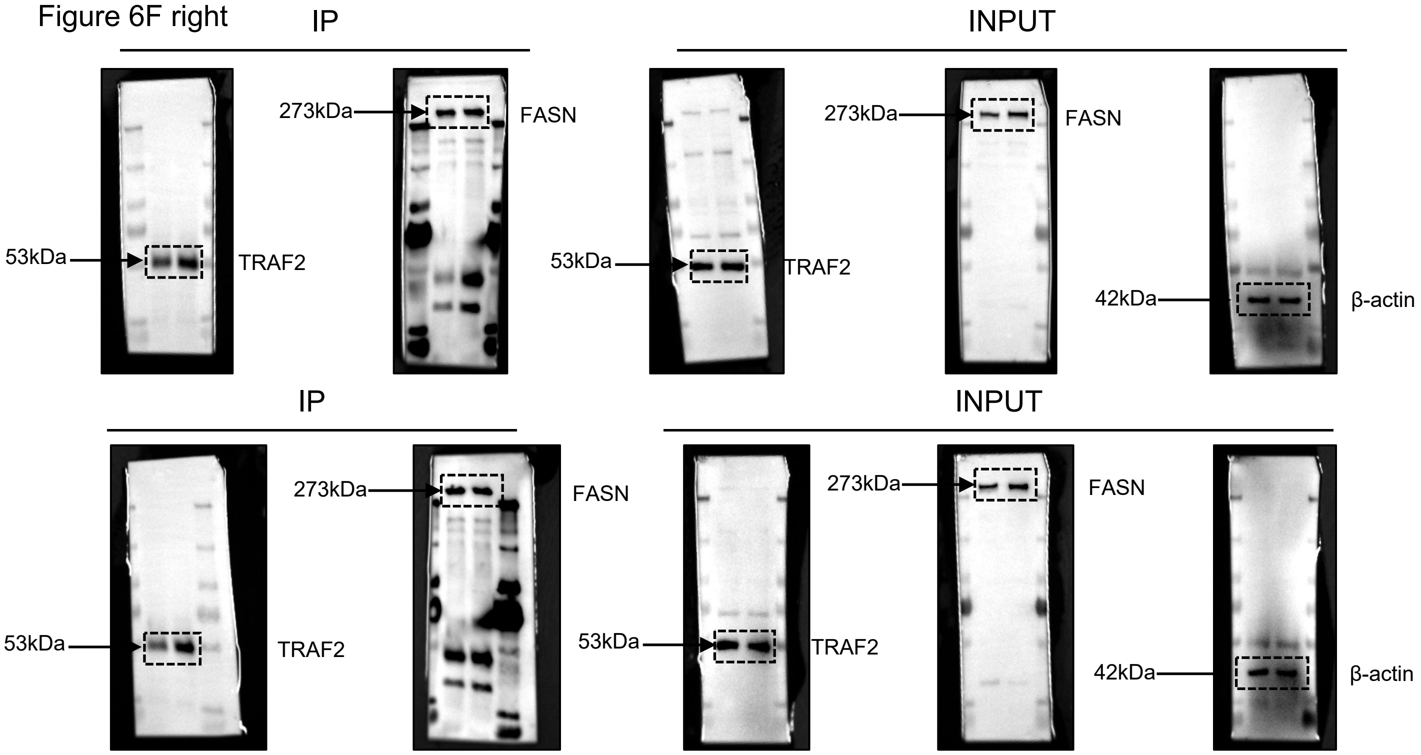

Figure 6G

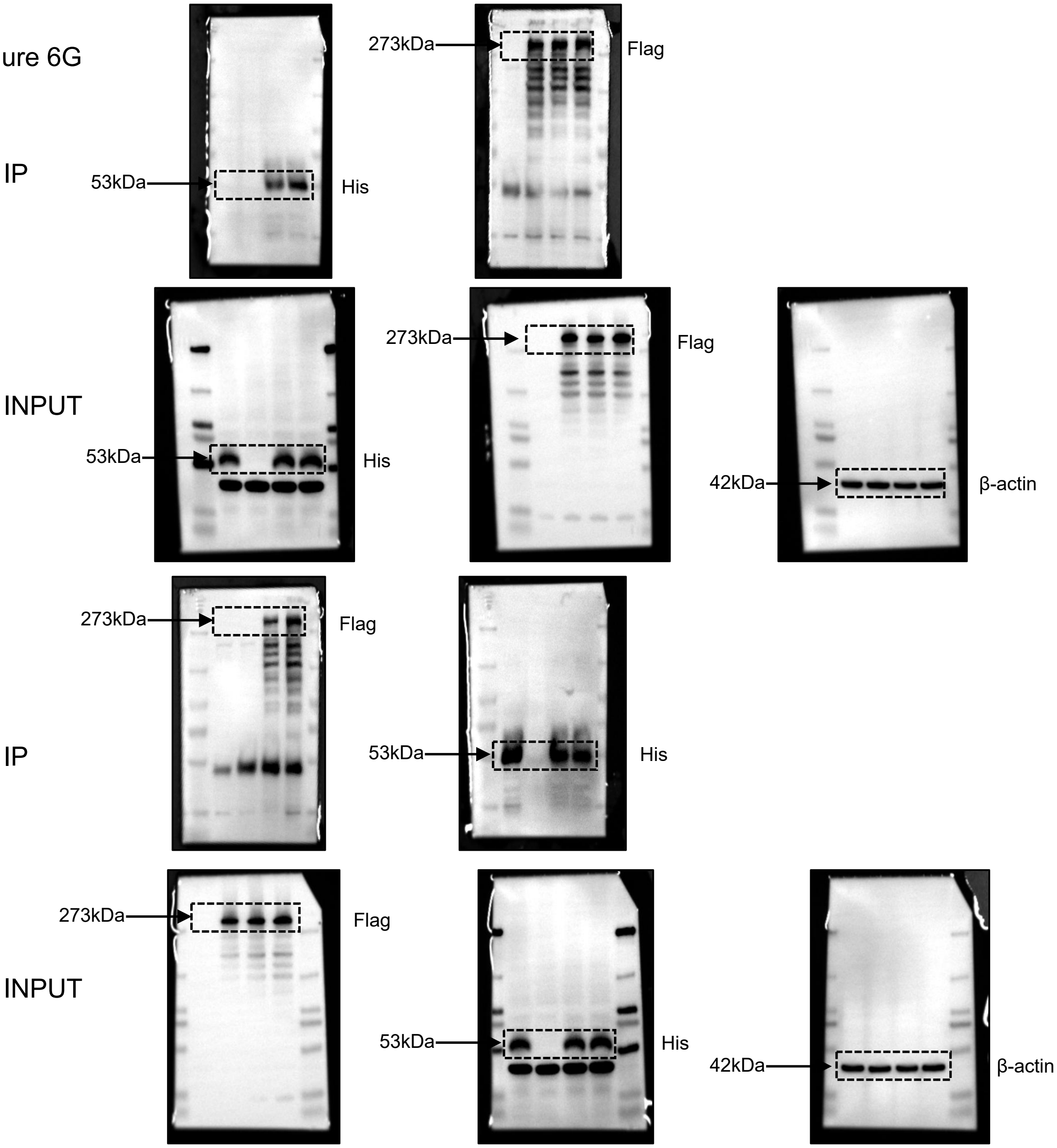

Figure 6H left

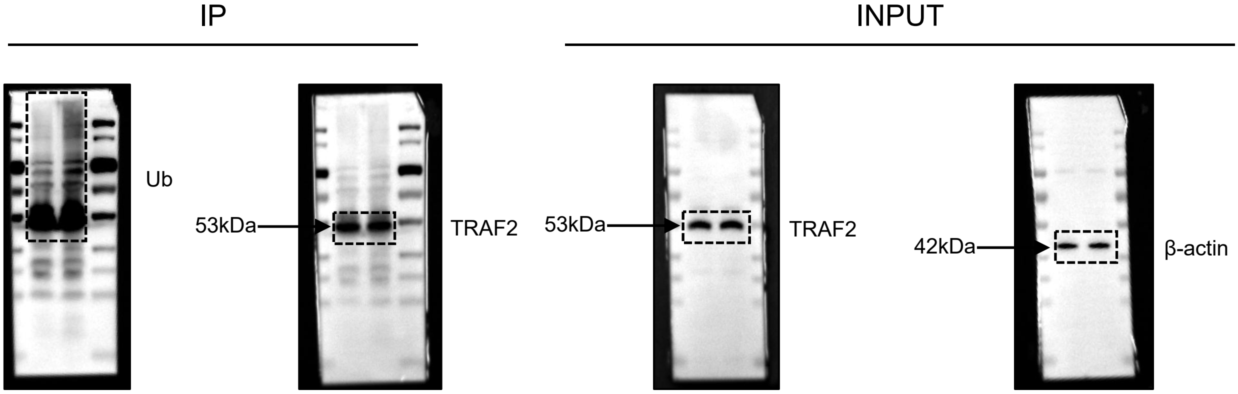

Figure 6H right

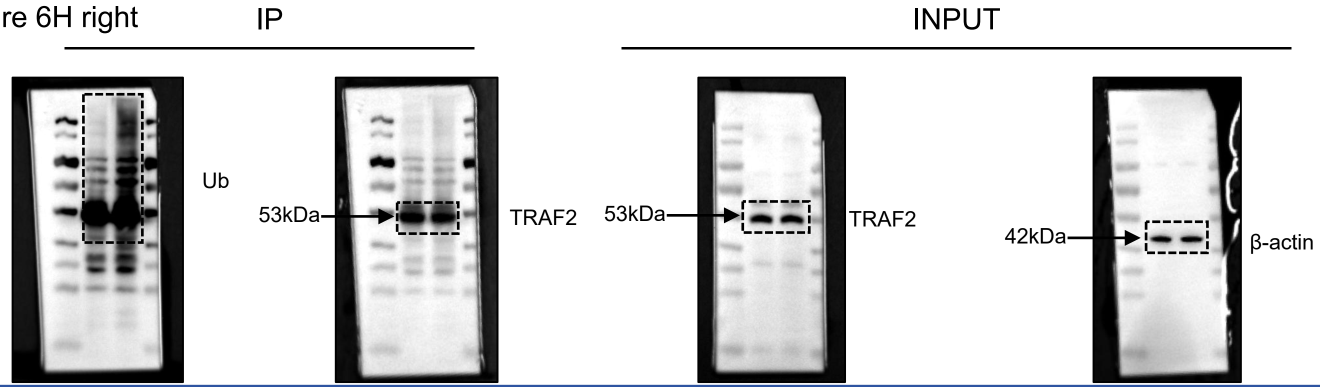

Figure 6I left

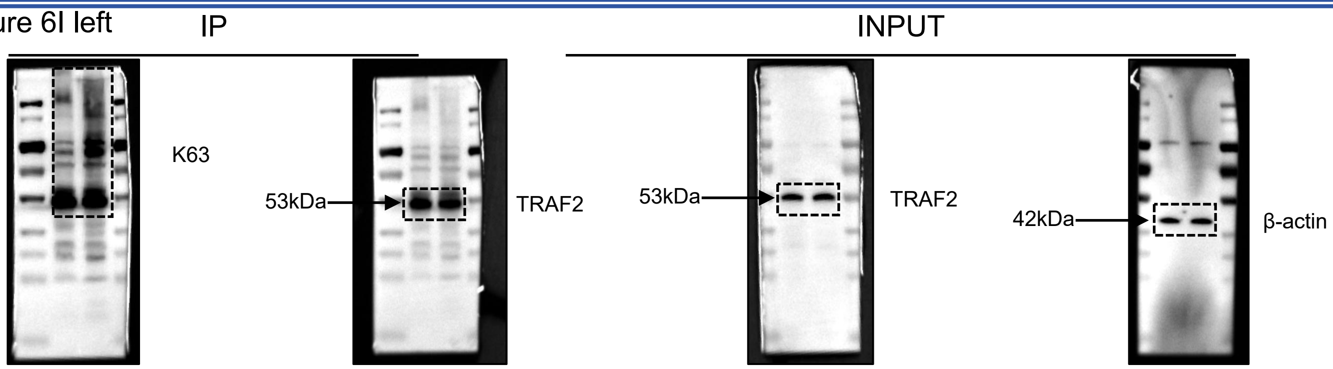

Figure 6I right

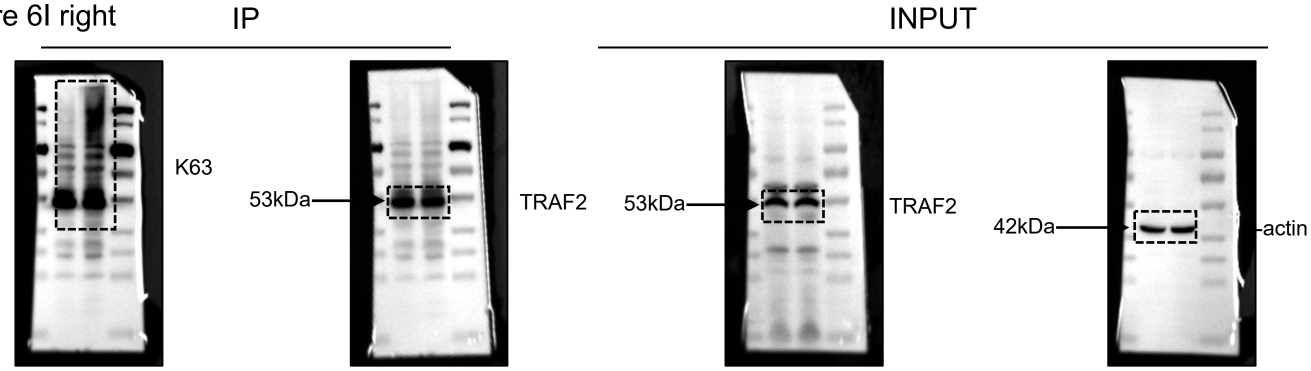

Figure 6J

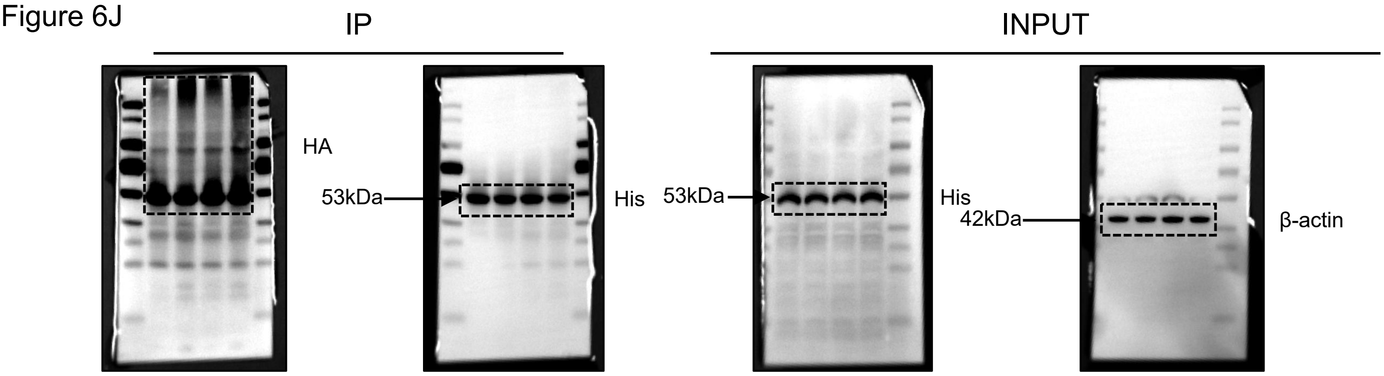

Figure 6K left

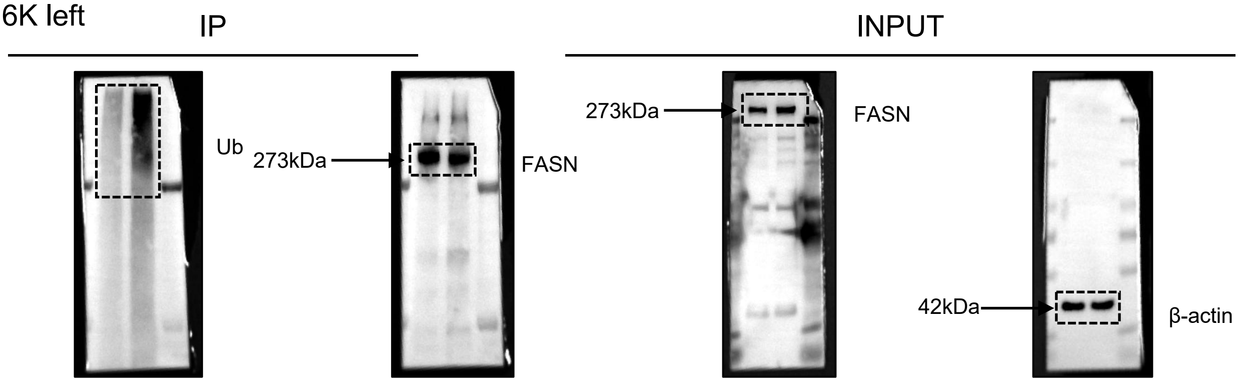

Figure 6K right

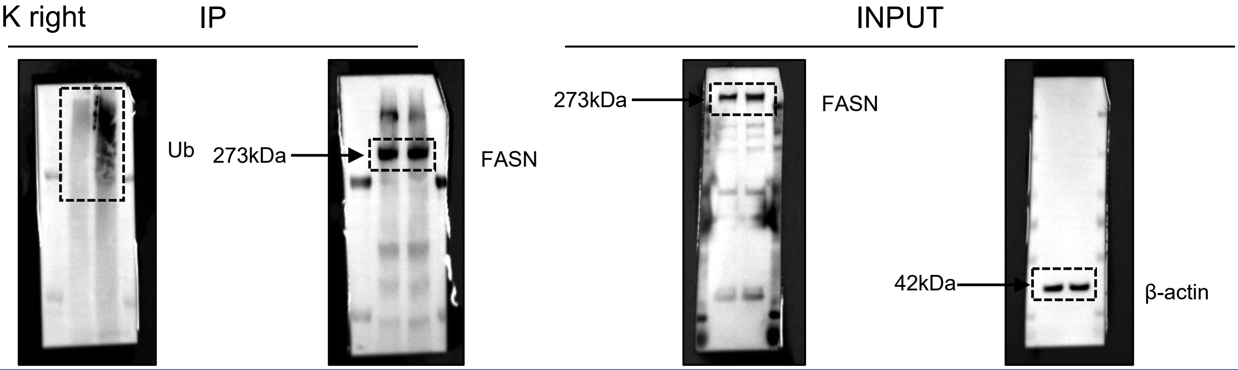

Figure 6L left

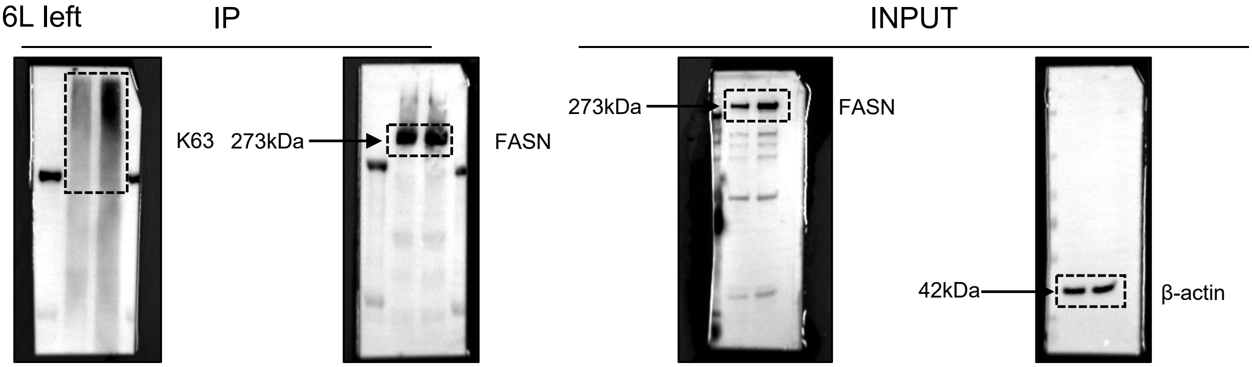

Figure 6L right

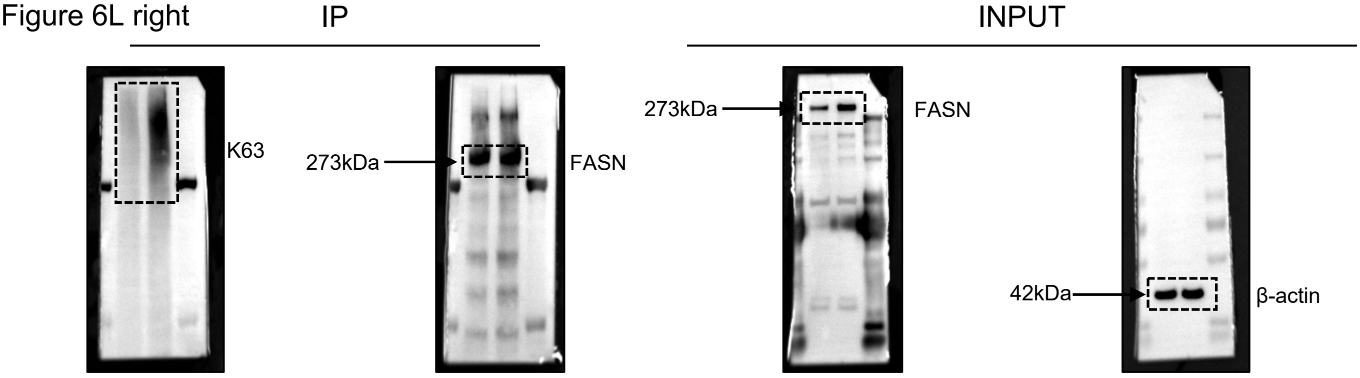

Figure 6M

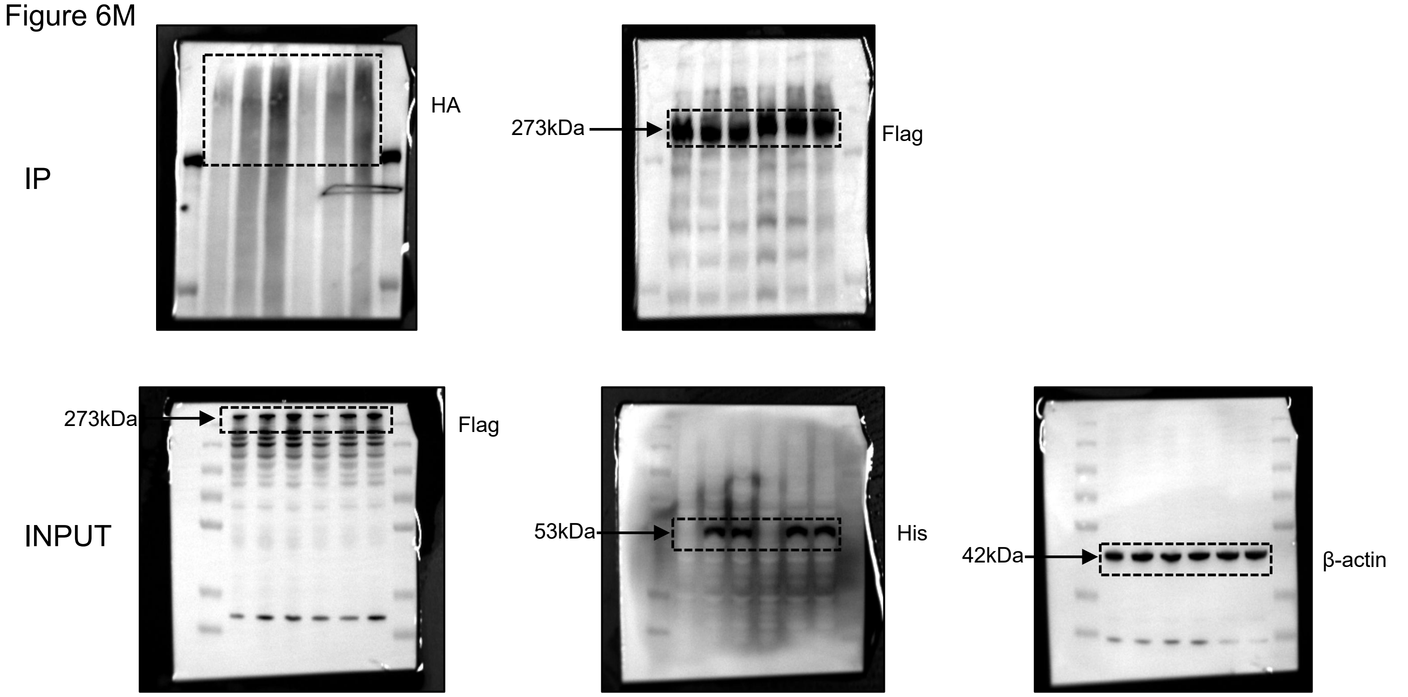

Figure 6N U251

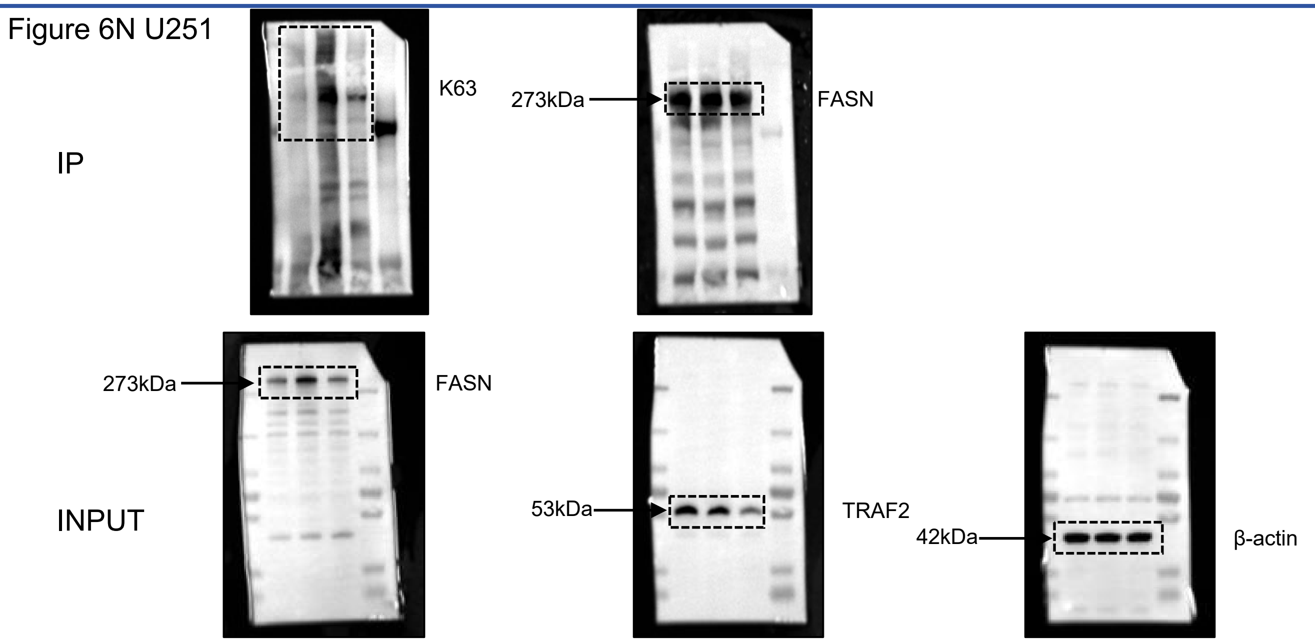

Figure 6N A172

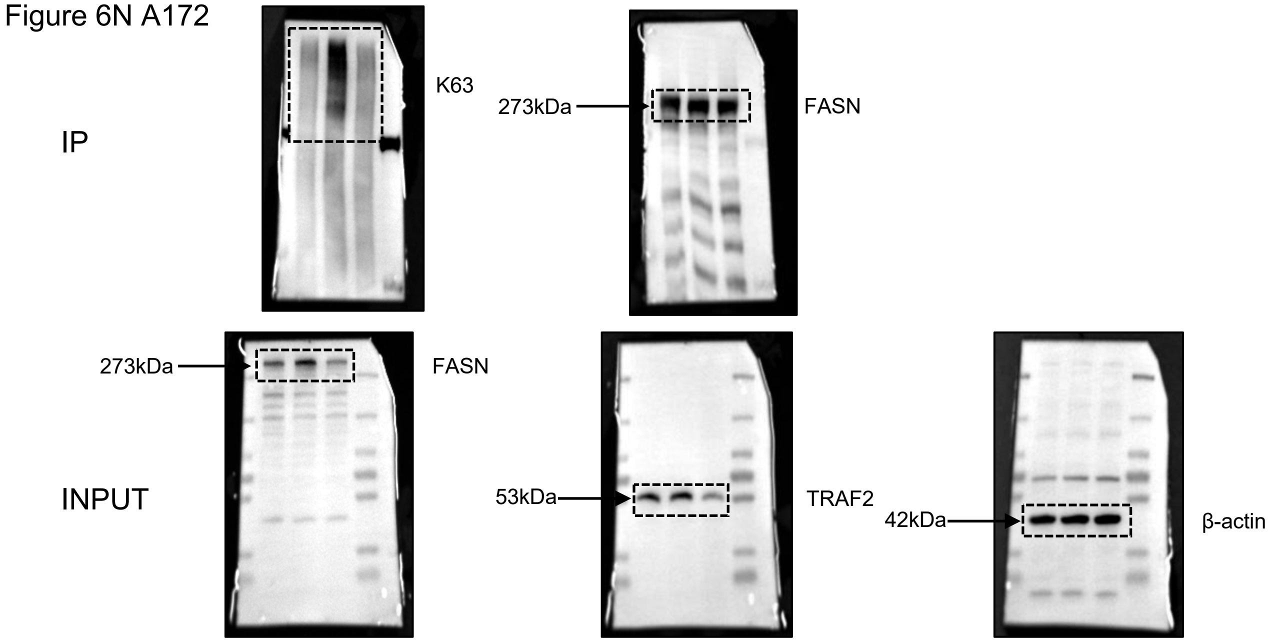

Figure 8B U251

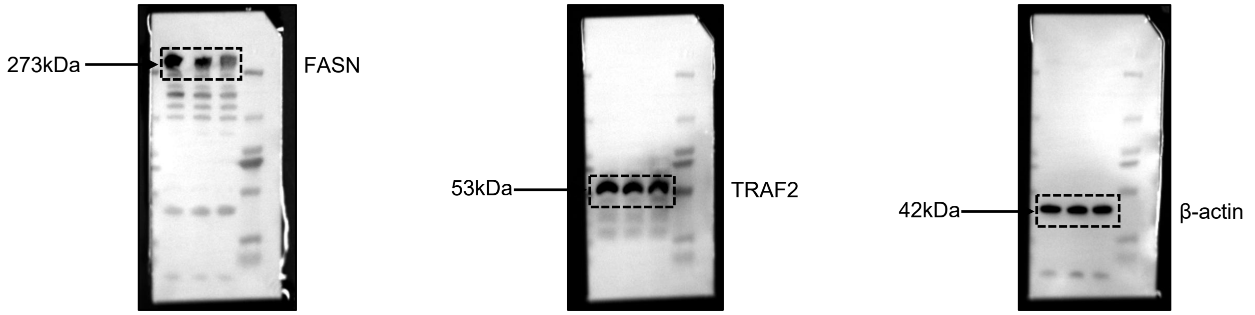

Figure 8B A172

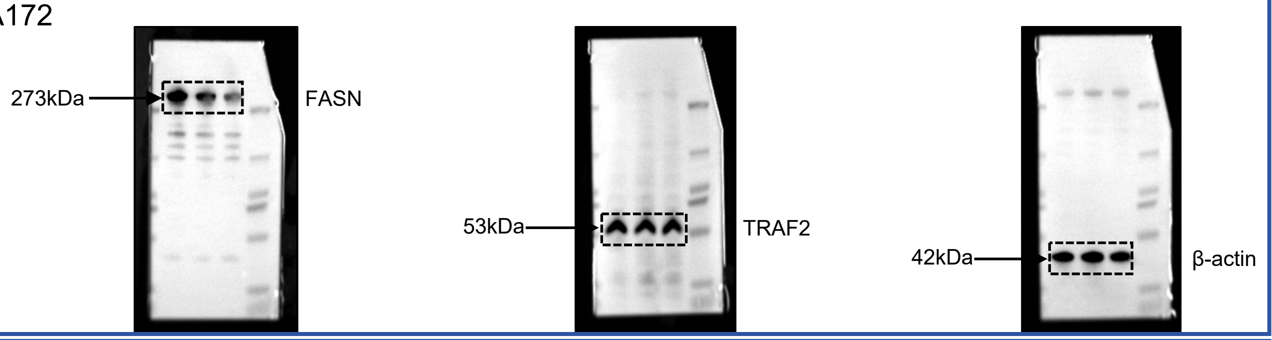

Figure 8C U251

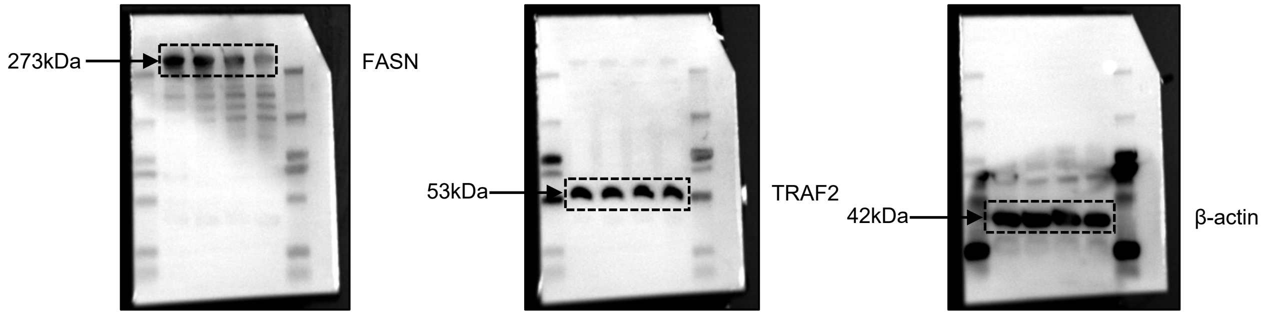

Figure 8C A172

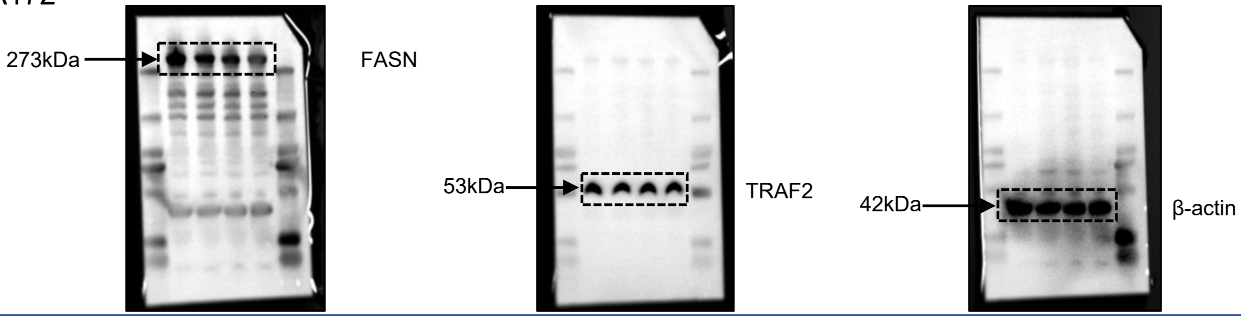

Figure 8D U251

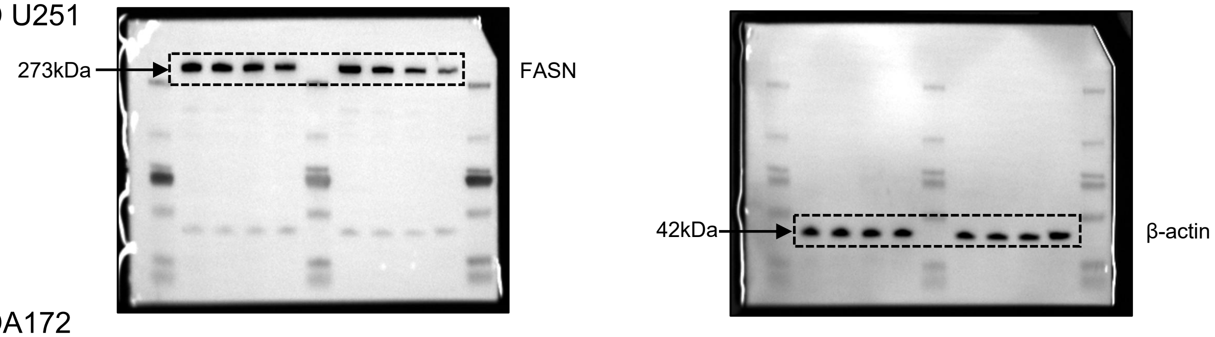

Figure 8DA172

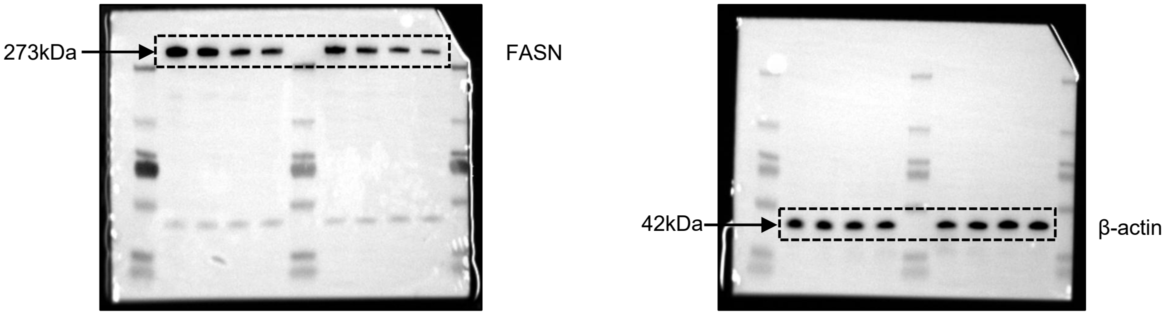

Figure 8E U251

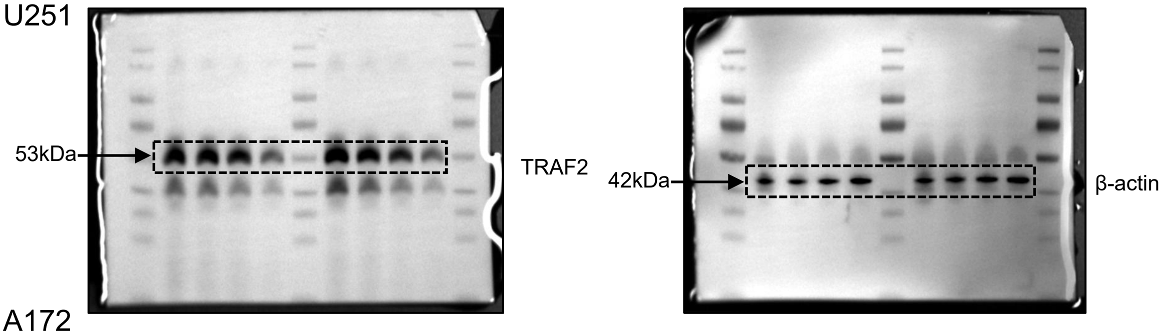

Figure 8E A172

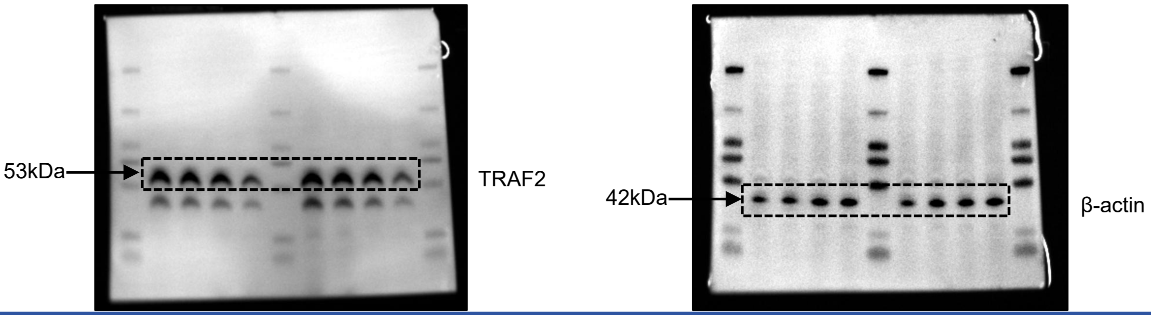

Figure 8F U251

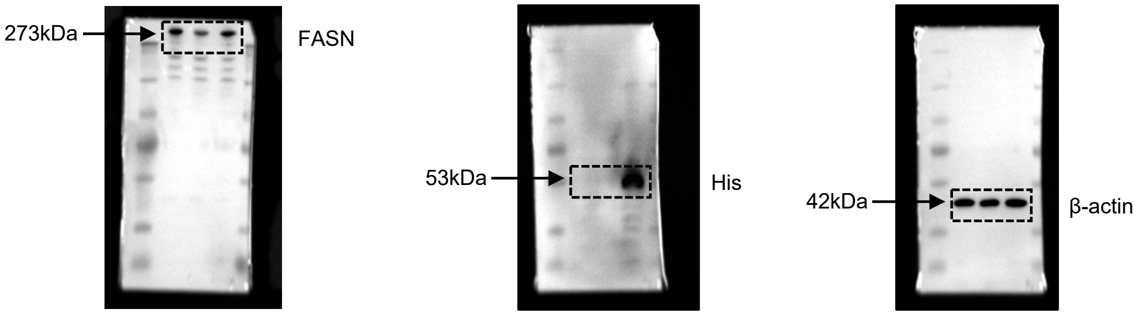

Figure 8F A172

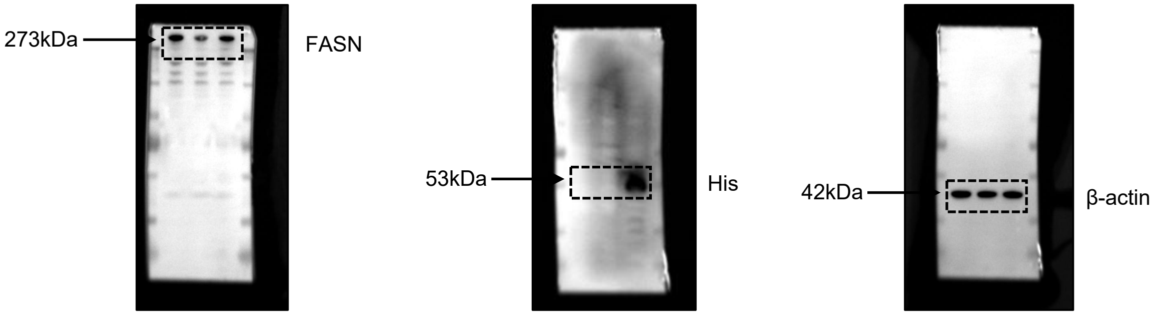

Figure 8G U251

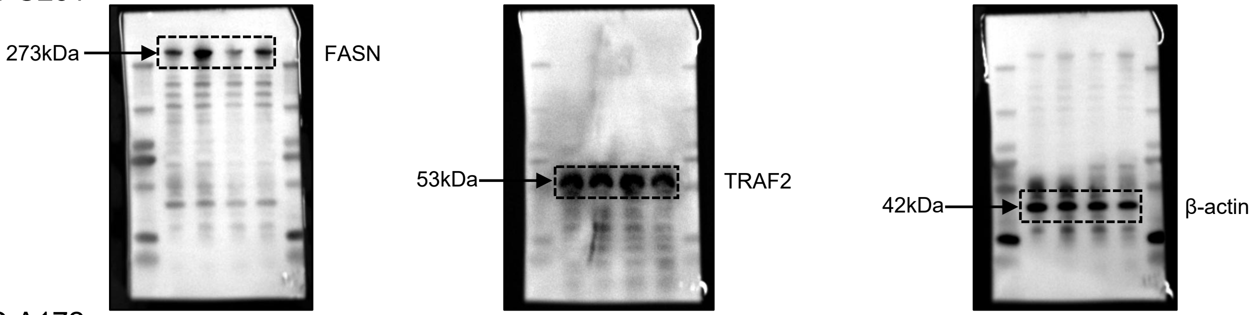

Figure 8G A172

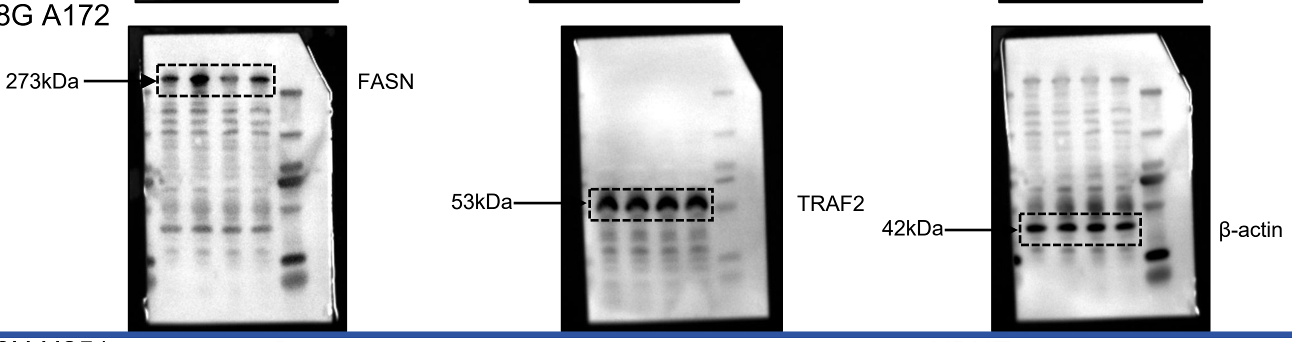

Figure 8H U251

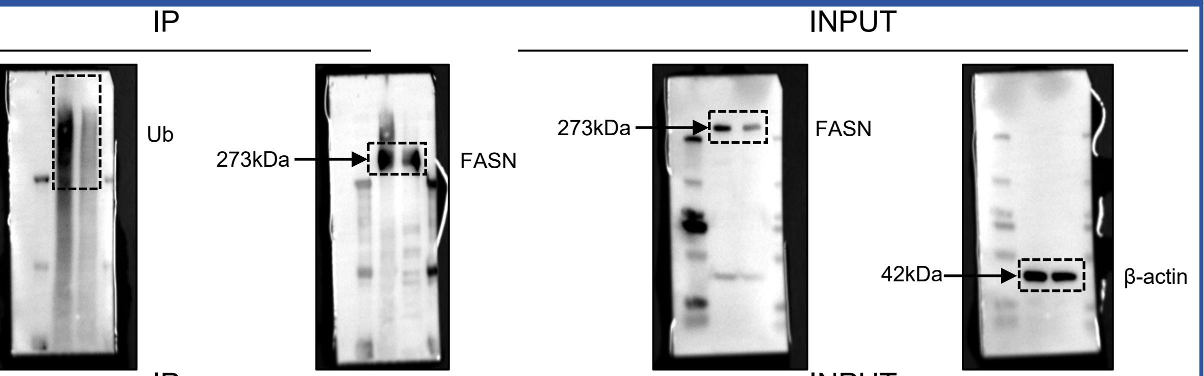

Figure 8H A172

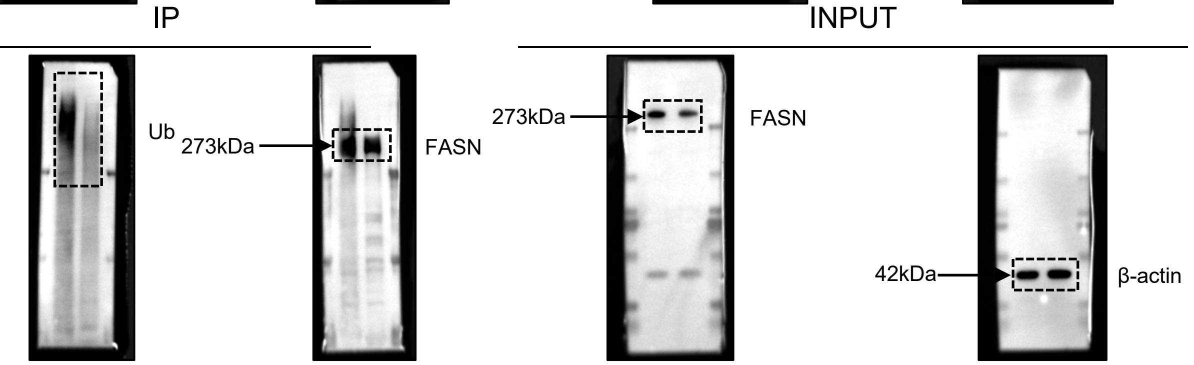

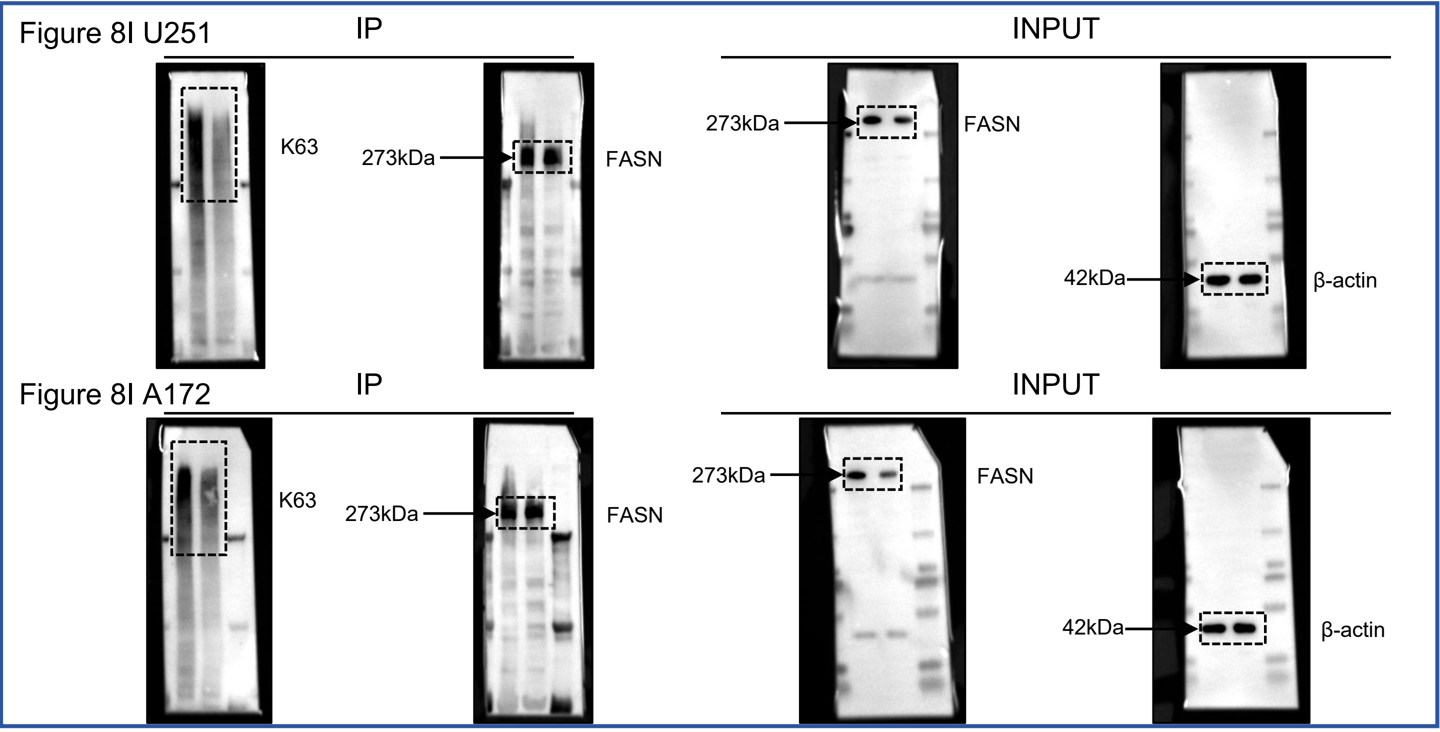
Figure 8I U251
IP
INPUT
K63
273kDa
FASN
273kDa
FASN
42kDa
β-actin
Figure 8I A172
IP
INPUT
K63
273kDa
FASN
273kDa
FASN
42kDa
β-actin

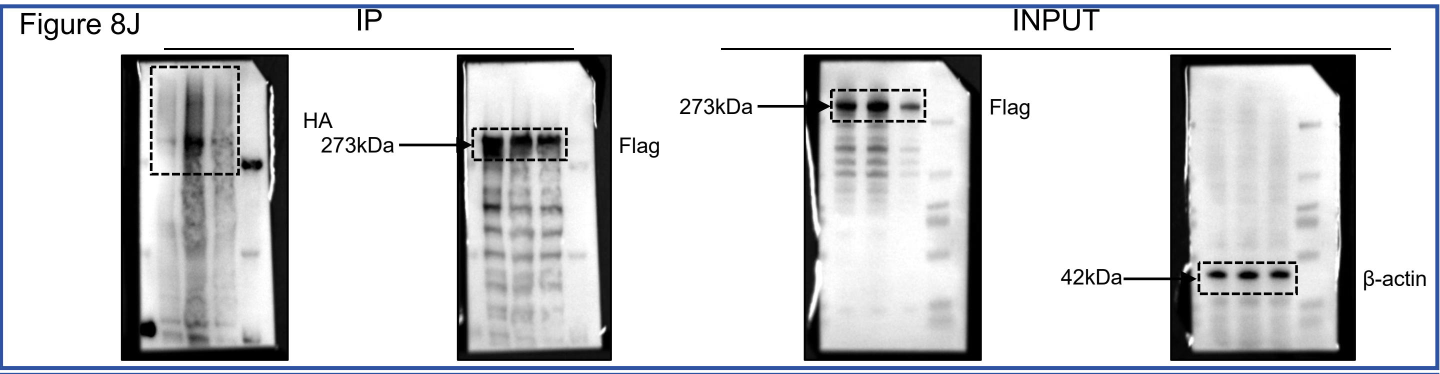
Figure 8J
IP
INPUT
HA
273kDa
Flag
273kDa
Flag
42kDa
β-actin

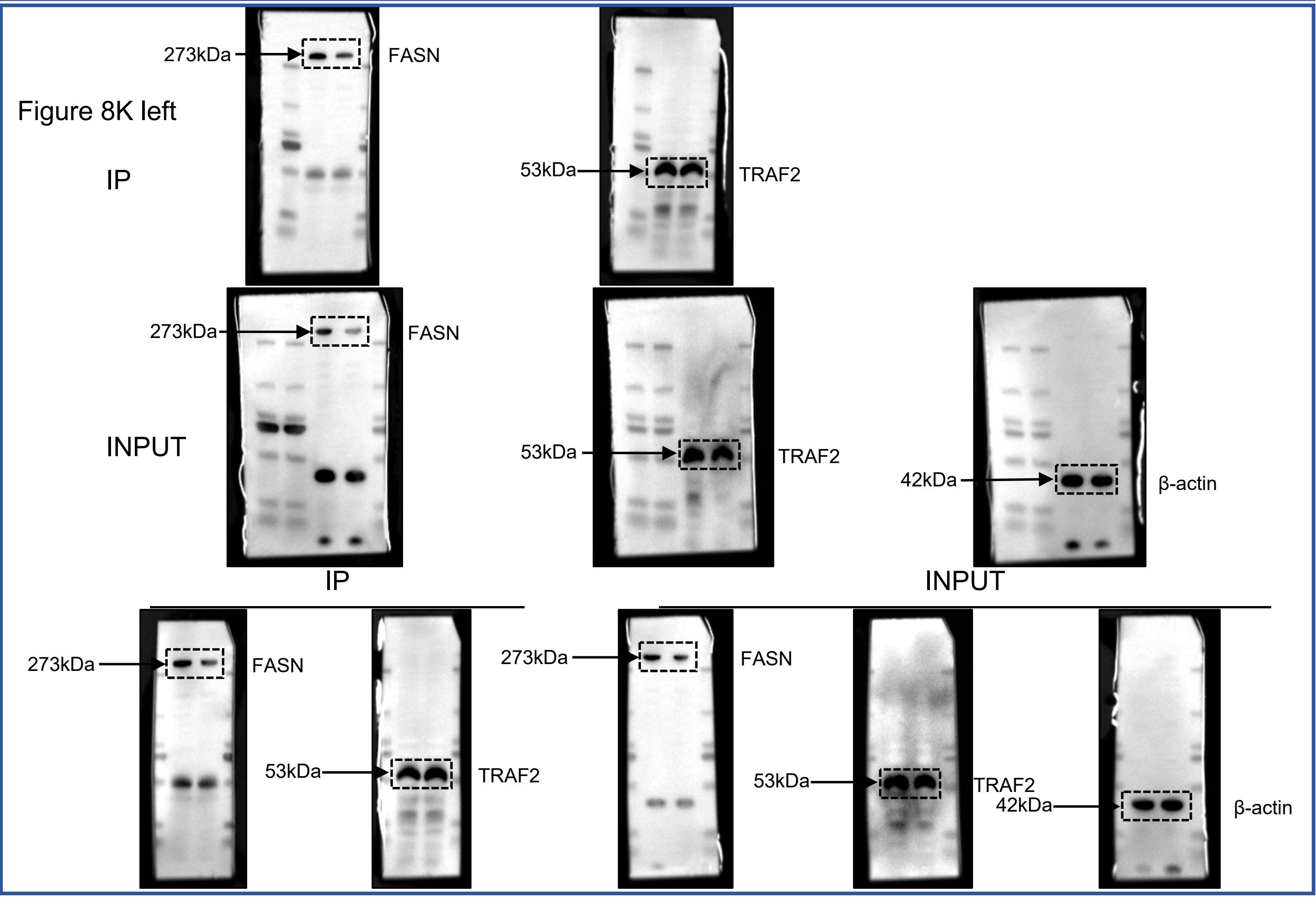
Figure 8K left
IP
273kDa
FASN
53kDa
TRAF2
INPUT
273kDa
FASN
53kDa
TRAF2
42kDa
β-actin
IP
INPUT
273kDa
FASN
53kDa
TRAF2
273kDa
FASN
53kDa
TRAF2
42kDa
β-actin

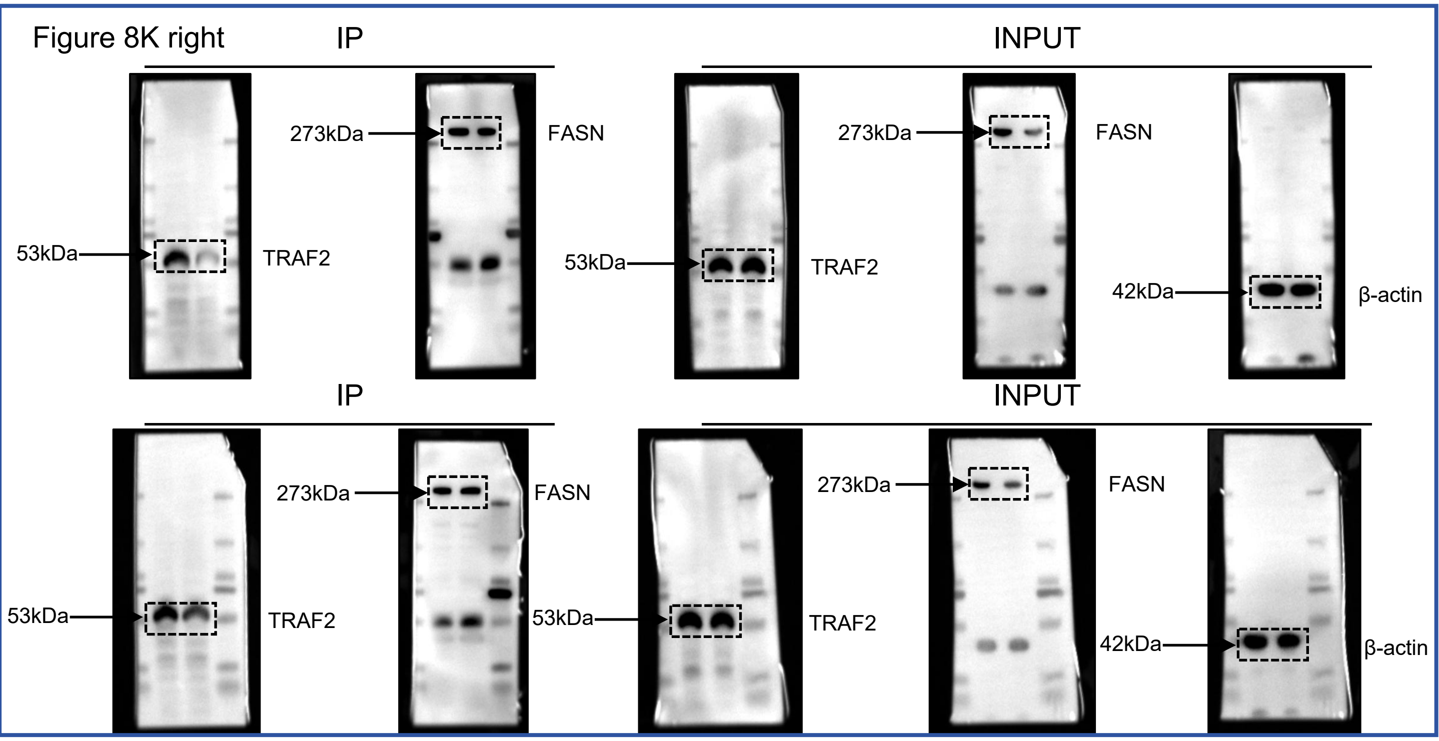
Figure 8K right
IP
INPUT
273kDa
FASN
53kDa
TRAF2
273kDa
FASN
53kDa
TRAF2
42kDa
β-actin
IP
INPUT
273kDa
FASN
53kDa
TRAF2
273kDa
FASN
53kDa
TRAF2
42kDa
β-actin

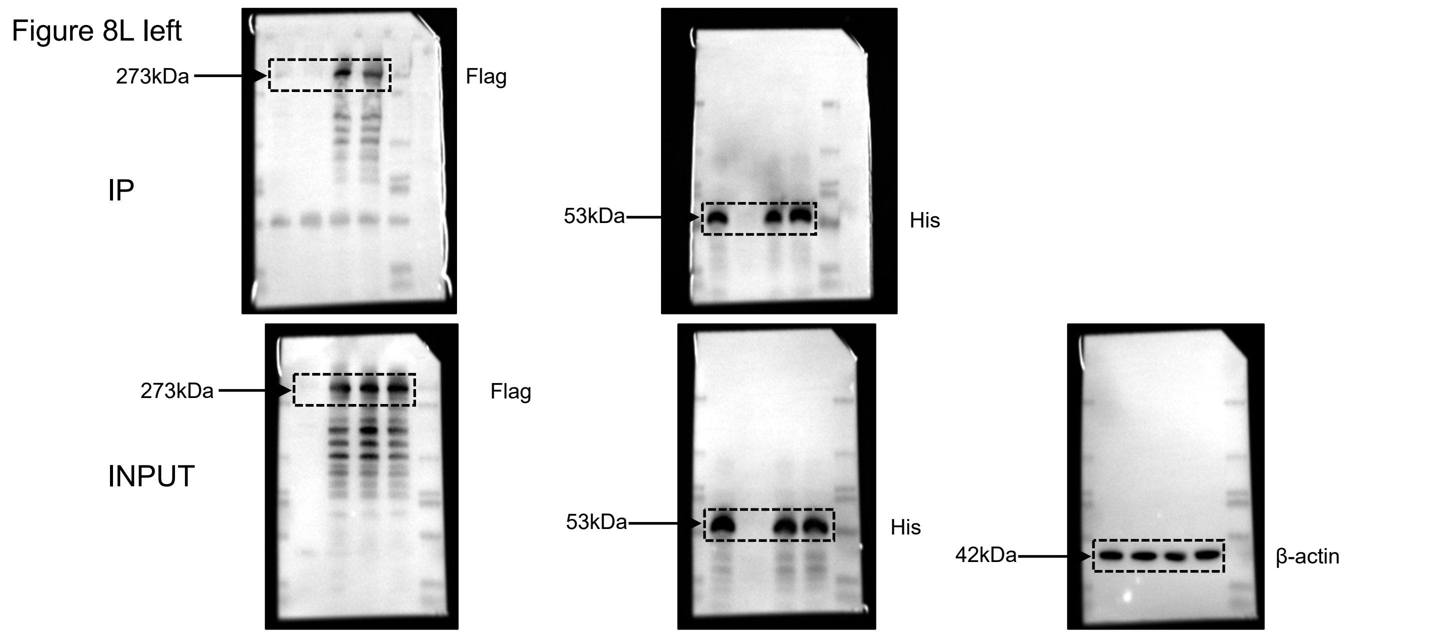
Figure 8L left
IP
273kDa
Flag
53kDa
His
INPUT
273kDa
Flag
53kDa
His
42kDa
β-actin

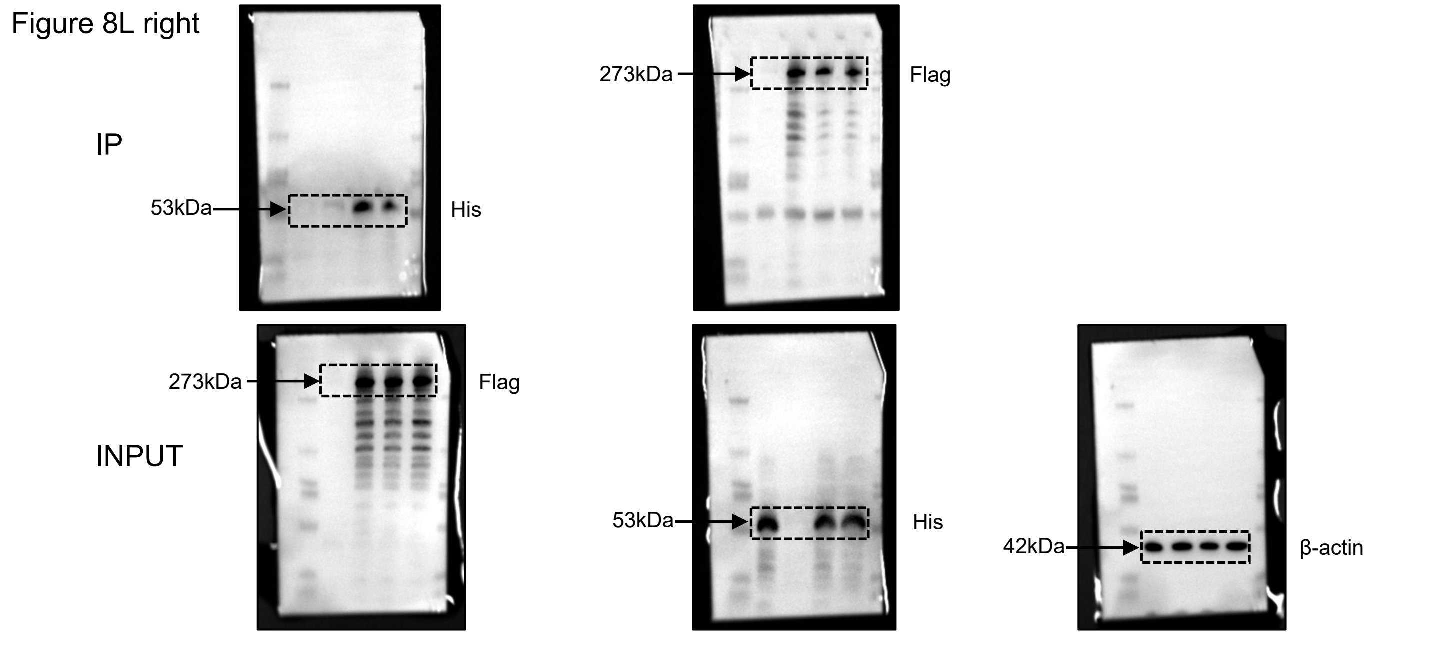
Figure 8L right
IP
53kDa
His
273kDa
Flag
INPUT
273kDa
Flag
53kDa
His
42kDa
β-actin

Supplementary Figure4

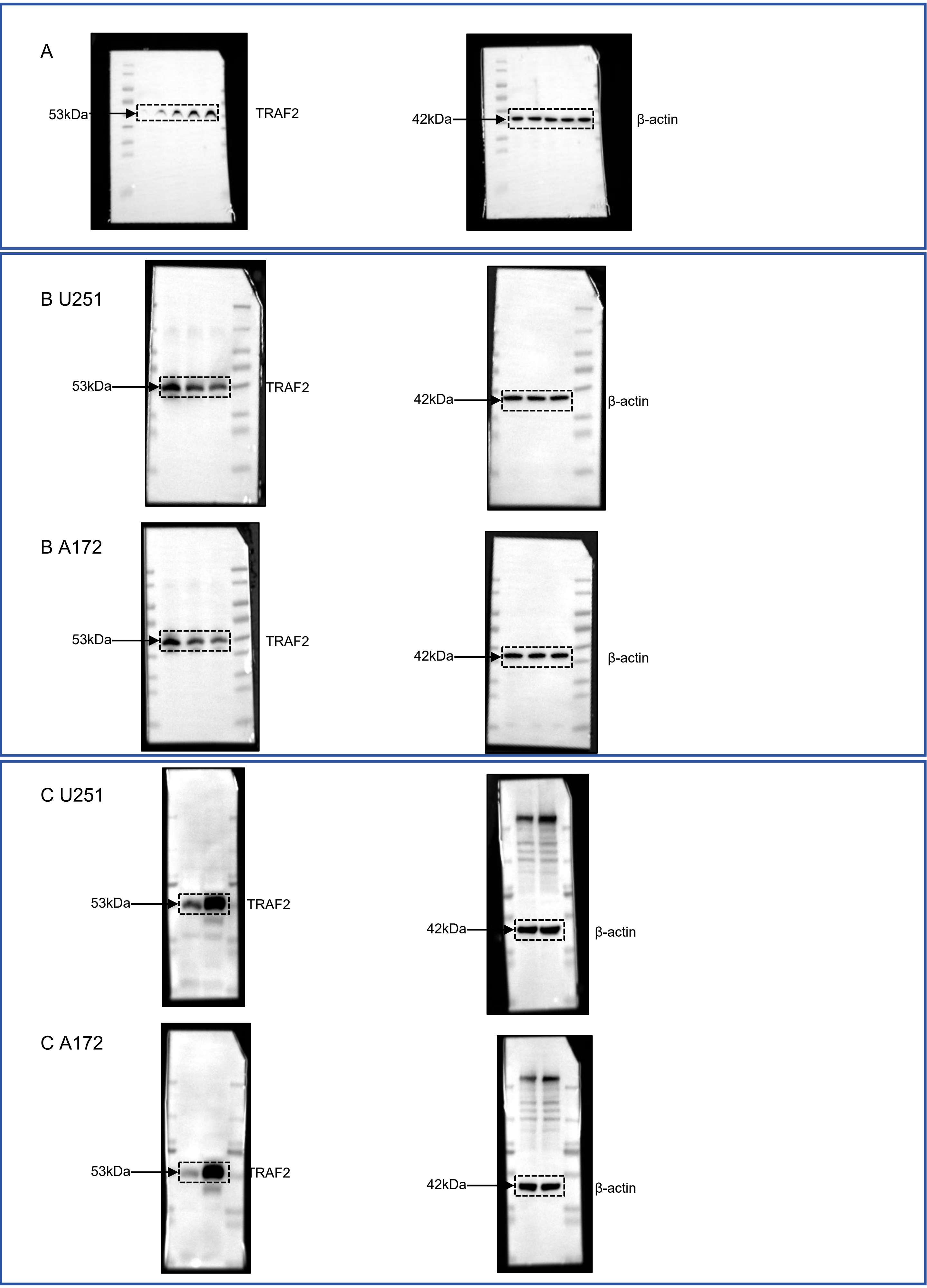

Supplementary Figure 6

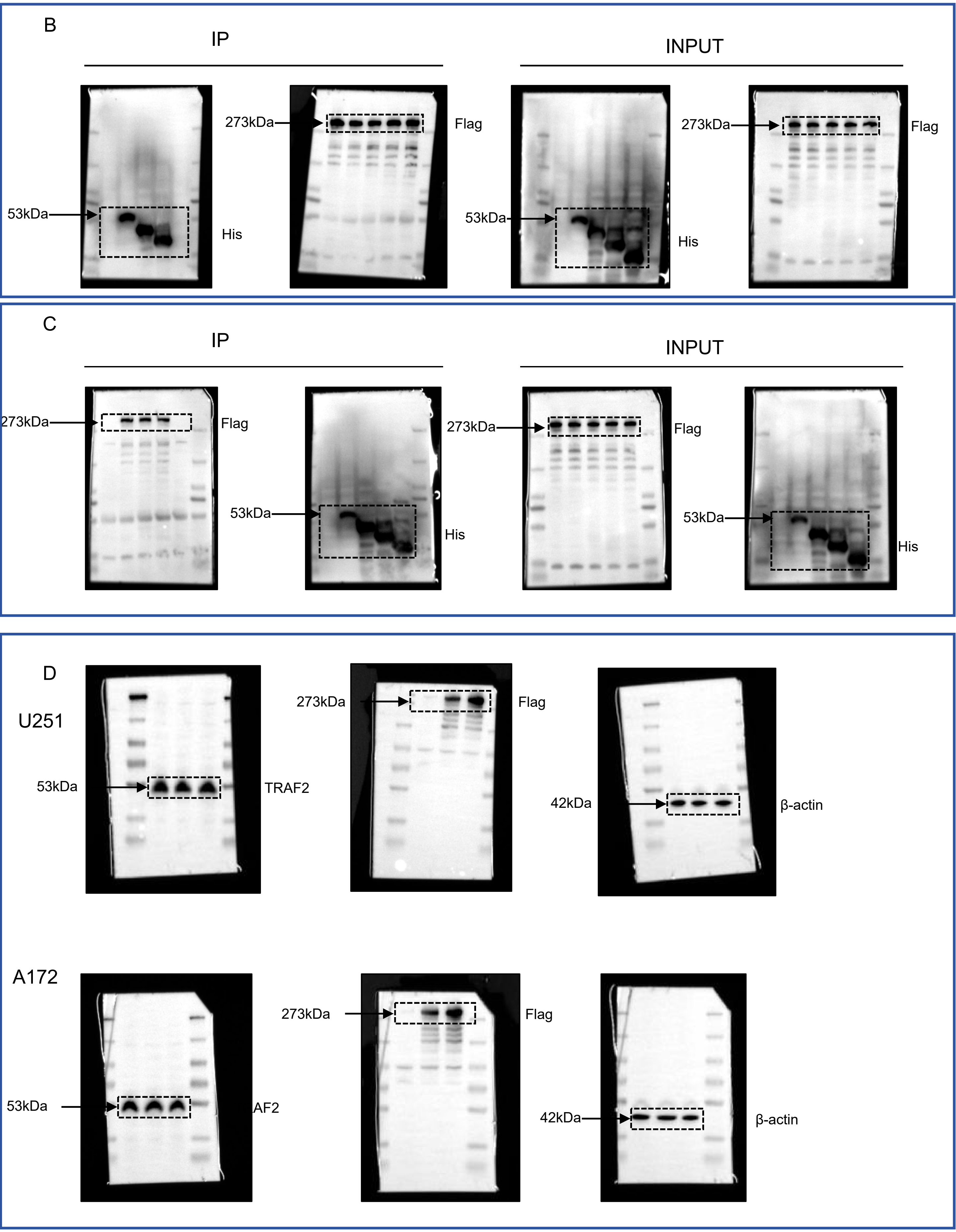

Supplementary Figure 6

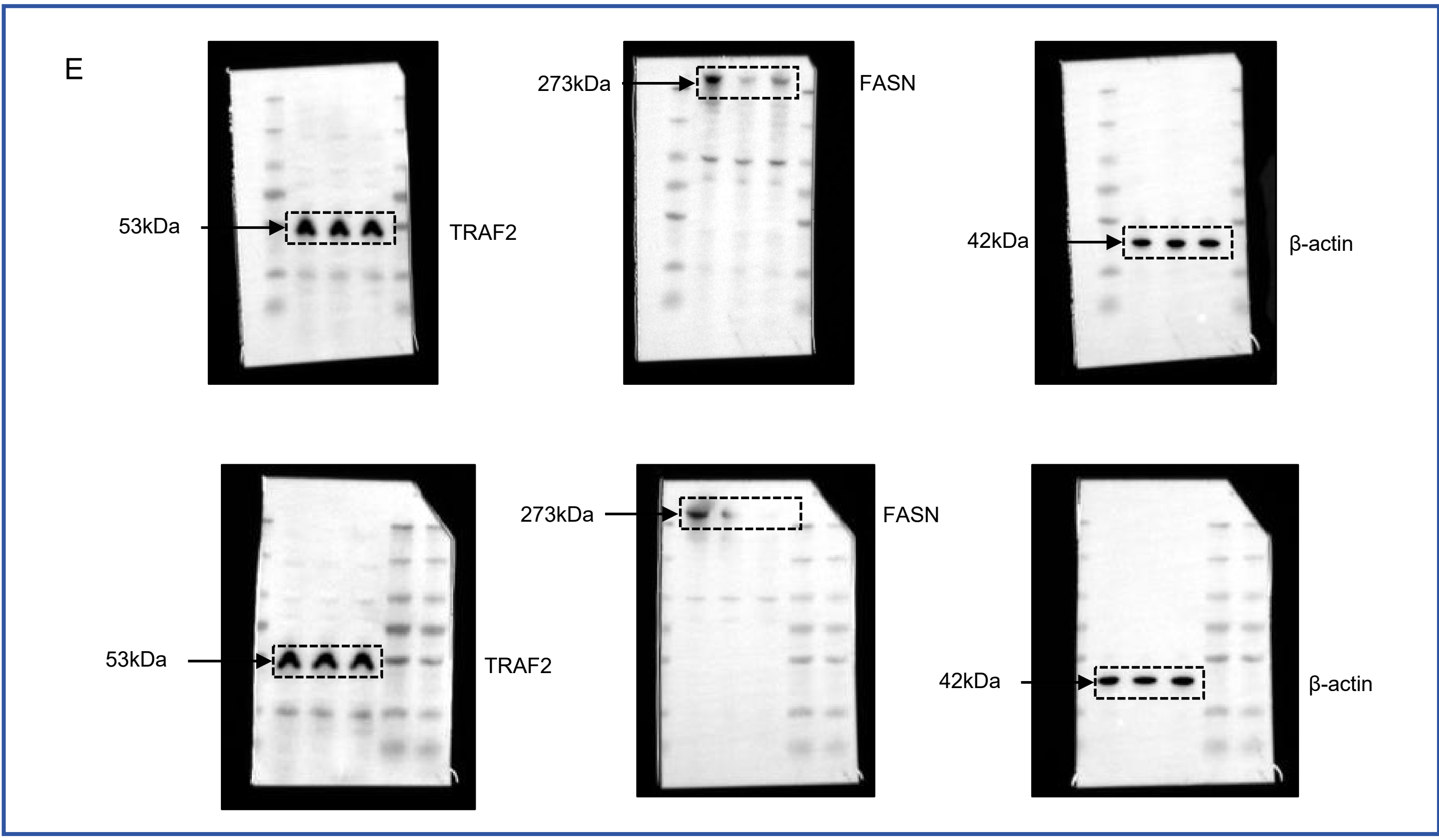

Supplement: Supplementary file 9 — Original Western blots [file 41420_2026_3087_MOESM9_ESM.pdf]
